# microBiomeGSM: the identification of taxonomic biomarkers from metagenomic data using grouping, scoring and modeling (G-S-M) approach

**DOI:** 10.3389/fmicb.2023.1264941

**Published:** 2023-11-22

**Authors:** Burcu Bakir-Gungor, Mustafa Temiz, Amhar Jabeer, Di Wu, Malik Yousef

**Affiliations:** ^1^Department of Computer Engineering, Faculty of Engineering, Abdullah Gul University, Kayseri, Türkiye; ^2^Department of Electrical and Computer Engineering, Faculty of Engineering, Abdullah Gul University, Kayseri, Türkiye; ^3^Department of Biostatistics, University of North Carolina at Chapel Hill, Chapel Hill, NC, United States; ^4^Division of Oral and Craniofacial Health Sciences, Adams School of Dentistry, University of North Carolina at Chapel Hill, Chapel Hill, NC, United States; ^5^Department of Information Systems, Zefat Academic College, Zefat, Israel; ^6^Galilee Digital Health Research Center (GDH), Zefat Academic College, Zefat, Israel

**Keywords:** gut microbiome, metagenomics, type 2 diabetes, inflammatory bowel disease, colorectal cancer, machine learning, classification, feature selection

## Abstract

Numerous biological environments have been characterized with the advent of metagenomic sequencing using next generation sequencing which lays out the relative abundance values of microbial taxa. Modeling the human microbiome using machine learning models has the potential to identify microbial biomarkers and aid in the diagnosis of a variety of diseases such as inflammatory bowel disease, diabetes, colorectal cancer, and many others. The goal of this study is to develop an effective classification model for the analysis of metagenomic datasets associated with different diseases. In this way, we aim to identify taxonomic biomarkers associated with these diseases and facilitate disease diagnosis. The microBiomeGSM tool presented in this work incorporates the pre-existing taxonomy information into a machine learning approach and challenges to solve the classification problem in metagenomics disease-associated datasets. Based on the G-S-M (Grouping-Scoring-Modeling) approach, species level information is used as features and classified by relating their taxonomic features at different levels, including genus, family, and order. Using four different disease associated metagenomics datasets, the performance of microBiomeGSM is comparatively evaluated with other feature selection methods such as Fast Correlation Based Filter (FCBF), Select K Best (SKB), Extreme Gradient Boosting (XGB), Conditional Mutual Information Maximization (CMIM), Maximum Likelihood and Minimum Redundancy (MRMR) and Information Gain (IG), also with other classifiers such as AdaBoost, Decision Tree, LogitBoost and Random Forest. microBiomeGSM achieved the highest results with an Area under the curve (AUC) value of 0.98% at the order taxonomic level for IBDMD dataset. Another significant output of microBiomeGSM is the list of taxonomic groups that are identified as important for the disease under study and the names of the species within these groups. The association between the detected species and the disease under investigation is confirmed by previous studies in the literature. The microBiomeGSM tool and other supplementary files are publicly available at: https://github.com/malikyousef/microBiomeGSM.

## 1 Introduction

A diverse community of trillions of microorganisms, including bacteria, archaea, viruses, as well as microbial eukaryotes like fungus, protozoa, and helminths, comprise the human microbiome. Human microbiome has an impact on overall human health and on homeostasis by influencing immunological function and by actively contributing to human metabolism (Marcos-Zambrano et al., 2021). Several disease-related conditions have been connected to a rupture in the stable interaction between gut epithelial cells and the gut microbiota (Petersen and Round, 2014). The number of microbiome-related studies has significantly risen in the last 10 years, and large population studies such as the American Gut Project (McDonald et al., 2018), the metagenomics of the Human Intestinal Tract (Qin et al., 2010), and the Human Microbiome Project (The Human Microbiome Project Consortium, 2012) have greatly expanded the amount of information currently accessible on the content and function of the human gut microbiome. The information from these studies is crucial for further research on host-microbiome linkages and how they relate to the commencement and evolution of many complicated diseases.

The community of microbes performs a variety of tasks for the host, including facilitating the uptake of nutrients (Martin et al., 2019), preserving homeostasis (Ohland and Jobin, 2015), fending off pathogens (Pickard et al., 2017), regulating immunological response (Mendes et al., 2019), among many others. Understanding these tasks and revealing the dialog between the bacterium and the host may help in developing plans for preserving the health status, treating diseases. In the last few decades, there has been an increased interest in researching microbial communities (and their associations) that live in various habitats, from the gut to the biosphere. Technological advancements lead to lower costs for 16S and metagenomic sequencing, greater sequencing resolution and depth (Levy and Myers, 2016). Synchronous development of brand-new techniques for high throughput characterization of different -omic data types, such as lipidomics, metabolomics, metagenomics, metatranscriptomics and metaproteomics (Muller, 2019) made this possible. However, it is a difficult task to experimentally detect the inter species microbe host associations due to several other difficulties relating to scale, scope, feasibility, and availability of samples for concurrent -omic readouts (Fritz et al., 2013). Computational approaches can circumvent some of these constraints, improving our knowledge of microbial associations (Dix et al., 2016).

The interactions between the host and the microbiome are critical factors affecting human health and disease. Therefore, recently there has been an exponential increase in microbiome studies. Many research efforts have been devoted to predicting disease based on taxonomic profiles derived from metagenomic sequencing data. In these studies, machine learning methods are used to predict the microbiome interactions associated with diseases. Beyond simply assessing their predictive capabilities using machine learning, these studies also highlight the importance of specific microbiomes as potential biomarkers for disease. In literature, there are numerous articles investigating microbiomes associated with three specific diseases: Colorectal Cancer (CRC), Type 2 Diabetes (T2D) and Inflammatory Bowel Disease (IBD). In particular, several studies aiming to uncover microbiomes related to T2D are summarized in Gao et al. (2018), Gurung et al. (2020), Cena et al. (2023), and Li R. et al. (2023). Microbiomes associated with CRC are reviewed in Huybrechts et al. (2020), Tabowei et al. (2022), Negrut et al. (2023), and Zwezerijnen-Jiwa et al. (2023). The studies of Soueidan and Nikolski (2016), LaPierre et al. (2019), Marcos-Zambrano et al. (2021), Lim et al. (2022), Hsu et al. (2023), and Mah et al. (2023) reviews the microbiomes associated with IBD.

More specifically, Deschênes et al. (2023) employed machine learning techniques to predict diseases by representing microbiomes using gene-based representations and taxonomic profiles. Through the creation of taxonomic profiles from shotgun metagenomic data, they identified significant taxa using their proposed methodology. They conducted experiments for five different diseases, namely type 2 diabetes, obesity, liver cirrhosis, colorectal cancer, and inflammatory bowel disease. For both IBD and CRC disease, the datasets used in Deschênes et al. (2023) are the same datasets used by the proposed approach in this study. In their study, they assessed the performance of nine distinct classifiers, including random forest, decision tree, two support vector machines with a linear kernel, random set coverage machine (rSCM), two logistic regressions, SVM with a radial basis function kernel (SVMrbf), and an ensemble algorithm derived from SCM (set coverage machine). For each dataset, they applied embedded feature selection techniques, such as random forest and ranking features based on resulting models, followed by machine learning model application. They reported improved classification performance for certain diseases by employing taxonomic profiling. The most effective results in taxonomic profiling were achieved using the random forest algorithm for liver cirrhosis, yielding an AUC of 88%. Their study demonstrated the effective use of converting microbiome data into taxonomic representation data for disease prediction. They reported that Lachnospiraceae microbiome is found as associated with T2D and it can be considered as a biomarker for this disease.

Sharma et al. (2020) predicted disease states using machine learning methods by examining related Operational Taxonomic Units (OTUs) at the same phylum taxonomic level, exploiting the connections among OTUs at this taxonomic rank. Their investigation focused on the relationship between disease and the microbiome, utilizing shotgun datasets for two distinct diseases, T2D and Cirrhosis. The dataset they chose for T2D analysis is the same as the dataset used by our proposed tool. They applied their proposed method, which they called “TaxoNN,” to a dataset with 174 cases and 170 controls for T2D (Qin et al., 2012) and a dataset with 118 cases and 114 controls for cirrhosis (Qin et al., 2014). TaxoNN is a Deep Learning based multi-layered approach to group OTU information based on phylum clusters. It trains clusters containing OTUs that share the same phylum separately using Convolutional Neural Networks (CNNs). It combines features from each cluster to enhance prediction accuracy via an ensemble learning technique. Their proposed method was evaluated using six different classifiers, including Random Forest, Gaussian Bayes Classifier, Naive Bayes, Ridge Regression, Lasso Regression, and Support Vector Machines. The TaxoNN method yielded the highest result, achieving an AUC of 92% for cirrhosis and 75% for T2D. Moreover, TaxoNN identified microbiomes at the level of three dominant phyla (Firmicutes, Proteobacteria, and Actinobacteria) for both diseases, highlighting their impact on the diseases.

Giliberti et al. (2022) investigated the influence of the relative abundance of microbial taxa on host phenotype classification using human metagenomes. They employed machine learning methods to construct species-level taxonomic profiles and accurately detected the presence of microbial taxa. In their evaluation scheme, they encompassed a total of 4,128 samples from 25 shotgun metagenomic datasets. Among the datasets used in their study, T2D dataset is same with the dataset used in this study. They also explored the effect on disease prediction using relative abundance values at three different taxonomic levels: genus, family, and order. Employing the Random Forest classification algorithm on species level dataset, they achieved the best performance for IBD dataset, across other datasets containing seven distinct disease categories (atherosclerotic cardiovascular disease, Alzheimer’s disease, Behçet’s disease, colorectal cancer, irritable bowel disease, type 1 diabetes, and type 2 diabetes). They identified statistically significant microbiomes for the diseases they identified. Among these microbiomes for these cases, the most significant result was obtained for Clostridium and this microbiome was followed by *Streptococcus* and *Ruthenibacterium*.

Pasolli et al. (2016) investigated the utility of microbiomes in disease prediction using metagenomic datasets for five different diseases: liver cirrhosis, CRC, IBD, obesity, and T2D. Among the datasets used in this study, T2D dataset is also utilized within this study. They conducted species-level prediction using microbiome profiles at the species level derived from metagenomic data. Their analysis encompassed a total of 2,424 shotgun metagenomic data samples from eight distinct studies. Employing cross-validation techniques, they compared classification outcomes using two widely employed classifiers in metagenomic data analysis, Random Forest and Support Vector Machine. In addition to these classifiers, they also evaluated the effectiveness of elastic network, neural network, and multiple regression methods. In addition to predicting diseases using microbiome data, they highlighted prominent microbiomes related to these diseases. Notably, they identified the Peptostreptococcus microbiome for colorectal cancer, the Streptococcus microbiome for T2D, and the Lachnospiraceae microbiome for IBD as influential microbiomes in disease prediction. Collectively, these papers advance our understanding for the potential role of the microbiome in these diseases using a variety of approaches and analyzes.

Identifying microbial taxa that may cause disease development and identifying microbial taxa whose impact varies depending on their abundance is one of the major goals of human microbiome studies. Uncovering the influence of taxons can help to the investigation of disease development processes and hence can contribute to the emergence of new approaches for prevention of these diseases (Zhang W. et al., 2022). Computational methods dealing with microbial relative abundances face several challenges in drawing meaningful conclusions due to their complex data structures and properties. Traditional computational methods are inadequate to assess microbiome population effects in isolation and to produce effective results without considering the diversity of the human microbiome. Recent research has used machine learning (ML) approaches to evaluate data from the human microbiome, more specifically to identify and understand the diversity of taxonomy and function within microbial communities, and to assess the impact of these factors on human health (Topçuoğlu et al., 2020). The use of ML in microbiome studies can be summarized as follows:

- ML models have been created to promote taxonomic representation and differentiation in microbiology.
- ML has been used for disease prediction by inferring host phenotypes.
- ML facilitates the characterization of disease-specific microbial signatures to classify patients based on microbial communities (Marcos-Zambrano et al., 2021).

In this paper, we present a novel approach, microBiomeGSM, to detect disease-associated taxonomic biomarkers by developing an efficient machine learning model based on the Grouping, Scoring and Modeling (G-S-M) approach. We have analyzed taxonomically transformed microbiome sequencing datasets with our proposed machine learning method. In this way, we aim to reveal the impact of the identified taxonomic biomarkers on specific diseases. To this end, our study contributes to the diagnosis and treatment of the disease under investigation. The proposed approach is applied on metagenomic datasets associated with 4 different datasets; and the taxonomic groups that have an impact on disease under study are identified. In the data preprocessing step, the MetaPhlAn tool developed by Ditzler et al. (2015) is used to extract taxonomic data from microbiome sequencing data. In the first component (grouping component) of microBiomeGSM, the species identified in a sample are grouped according to the level of taxa known to be associated with them. In the second component (scoring component) of microBiomeGSM, importance scores are assigned to taxon groups using inherent machine learning techniques. The score is a predictor of how well a sample can be classified based on the abundance values of the species included in that taxon group. In the final (modeling) component of microBiomeGSM, three different outputs are generated. The first output is the performance metrics of the developed machine learning model. The second output is the list of important taxa groups associated with the disease under study, and these taxonomic features can be considered as biomarkers. The third output is the species associated with the taxa groups. Performance evaluation of microBiomeGSM is assessed separately for each disease, and for 3 different taxonomic levels (genus, family, order). Feature selection algorithms are applied to the same dataset in order to comparatively evaluate the performance of microBiomeGSM. The biological relevance of the identified taxon groups at genus, family, order levels for different diseases is discussed with reference to existing knowledge in the literature.

## 2 Materials and methods

### 2.1 Dataset

The data used in this study are obtained from the NCBI Sequence Read Archive (SRA045646, SRA050230) provided by Qin et al. (2012) for T2D; accession number PRJNA398089 in the SRA for the Integrative Human Microbiome Project for IBDMDB (Beghini et al., 2021). IBD dataset is obtained from the MetaHit project (Marco-Ramell et al., 2018) (ERA000116). The CRC metagenomic dataset containing 1,262 samples was created by Beghini et al. (2021). Microbiome sequencing data is classified into disease states based on the metadata associated with them. To ensure data quality, we applied quality filtering to meet the standards outlined in the Human Microbiome Project Consortium SOP (2012), as referenced in Thomas et al. (2019). This procedure allowed us to categorize the raw sequencing data according to relevant disease states, enabling our subsequent analyzes. The microbiome samples were associated with the microbial species of origin (taxa) using the MetaPhlAn tool, and the relative abundance composition for each taxon was generated accordingly. These taxa and their relative abundances serve as features or variables in our machine learning approaches. MetaPhlAn first assigns reads to microbial clusters using clade-specific genes for assignment. It then presents the relative abundance of microbial taxa based on these readings. In this study, the assignment to microbial species of origin (taxa) was determined for each DNA sequence using the MetaPhlAn tool. The relative abundance value is normalized by dividing the number of reads for each taxonomic level by the total number of reads for only one sample. In this way, the taxonomic abundance values are expressed as real numbers in the range [0,1] with a sum of 1 for each sample. Samples with less than 1 million total reads were not included in our study. For each sample, we determined the diversity of disease-relevant microbiomes, where diversity represents the presence and relative abundance of microorganisms (Alatawi et al., 2022).

The four microbiome datasets used to evaluate the microBiomeGSM tool are listed in Table 1. The table presents the number of samples in each dataset and the number of samples that are labeled as positive. Positive samples refer to patients, while negative samples refer to controls. Each dataset contains the abundance values of the species, which we consider as features. We have considered 3 taxonomic levels for creating the groups, i.e., genus, family, and order. For each dataset, the number of extracted groups is listed in the corresponding column, while ‘-’ denotes missing information.

**Table 1:** The list of datasets used to test the model.

#	Dataset	# of Samples	# of positives	# of features (Species)	# of Groups (Genus)	# of Groups (Family)	# of Groups (Order)
1	CRC	1,262	600	912	261	100	49
2	IBDMDB	1,638	1,209	579	187	77	43
3	IBD	382	148	1,456	448	177	84
4	T2D	290	155	1,456	448	177	84

Number of samples who have positive class label are shown in the second column. The number of features/Species is shown in the third column. Number of groups created at Order, Family and Genus taxonomic levels are listed at the 4-6th columns, respectively.

Statistical information regarding the numbers of features in each group is given in Table 2. For each data set and for each taxonomic level (genus, family, and order), the average, maximum, and minimum numbers of features within a group are given.

**Table 2:** Statistical information about the numbers of features within a group, shown separately for each taxonomic level.

#	Dataset	Genus (avg/max/min)	Family (avg/max/min)	Order (avg/max/min)
1	CRC	3.51 /52/1	9.16 /76/1	18.71/202/ 1
2	IBDMDB	3.09 /34/1	7.50 /64/1	13.44/163/ 1
3	IBD	3.24 /61/1	8.22 /65/1	17.32/195/1
4	Type 2 diabetes	3.24 /61/1	8.22 /65/1	17.32/195/ 1

Sen is the sensitivity, Spe is the specificity, AUC is the Area Under the Curve.

Supplementary Table S1 shows the distribution of the groups based on their sizes for the IBDMDB dataset. The numbers in the table indicate the number of groups that have the specified number of species for that specific taxonomic level. There are 187, 77, and 43 groups for genus, family and order levels, respectively. About 90% of the groups at the order level, about 90% of the groups at the family level, and about 97% of the groups at the genus level contain 20 or fewer species for the IBDMDB dataset.

### 2.2 microBiomeGSM

Our proposed method, microBiomeGSM, consists of three main components: Grouping, Scoring, and Modeling (G-S-M). The G-S-M approach has been used in other studies that consider the pre-existing biological knowledge (Yousef et al., 2019, 2021a,c, 2022a; Qumsiyeh et al., 2022; Yousef and Voskergian, 2022; Ersoz et al., 2023; Jabeer et al., 2023). Additionally it was modified to integrate two-omics datasets such as the miRcorrNet and miRModuleNet tools (Yousef et al., 2021a, 2022b); and even to integrate 3 omics datasets such as 3Mint tool (Unlu Yazici et al., 2023). Interested readers can find further details about those approaches in our recent reviews (Yousef et al., 2021b; Kuzudisli et al., 2023).

**Figure 1:**
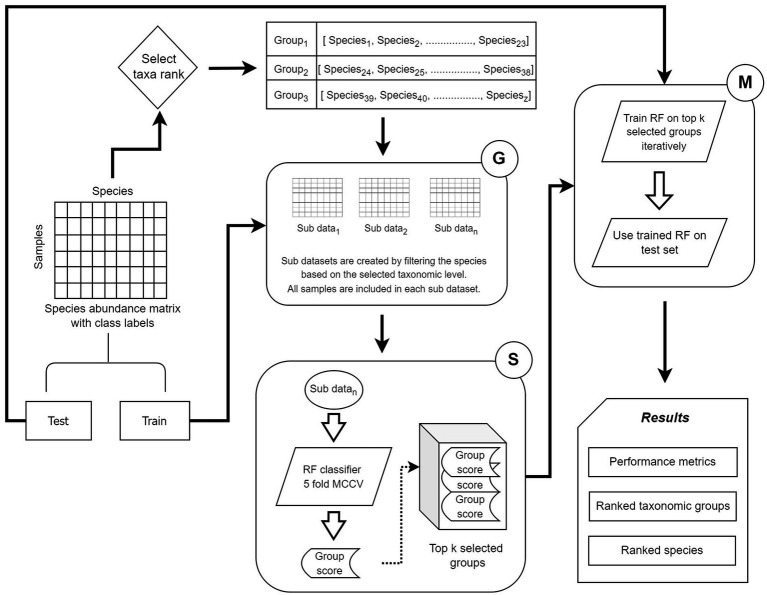
G-S-M approach in microBiomeGSM. MCCV denotes Monte Carlo Cross-Validation.

Utilizing the G-S-M approach, microBiomeGSM performs a search to identify the most important taxonomic groups in disease-associated metagenomic datasets. The relative abundance values of the species within the group can be checked for each sample; and the generated model decides whether the sample has the disease or not. By focusing on a specific taxonomic level, we can use the G component to find the most significant group for the disease under study. This approach provides the advantage of focusing on either the macroscopic or microscopic view of the most important group to distinguish between healthy samples and patient samples. An overview of the steps performed in microBiomeGSM is presented in Figure 1.

Let X be the two-class dataset consisting of the species in the columns, and samples in the rows including the class labels (1 denoting the disease state and 0 denoting the healthy state). To understand the approach in detail, let us assume that the taxonomic level is selected as “genus” for the “Select taxa rank” step in Figure 1. The input X_abd_ (abundance matrix) is first split into a training set (X_train_) and a test set (X_test_) with a ratio of 80:20 based on the class labels. Denote by S the feature space of all species in X_abd_ and by U_genus_ all unique genera for S. Grp{} denotes the selection function of each U_genus_ in S, grouping all species on the basis of similar genuses. Grp{U_genus_^i^ for S} represents each genus in S, with all the species grouped by genus. For example, if we take Alistipes as one of the genus in U_genus_, we get the following when we apply the Grp function.

Grp{U_genus_^i^}, where i = Alistipes and ∈ S.

Grp{Alistipes} = {alistipes_finegoldi, alistipes_indistinctus, alistipes_inops, alistipes_shahii}.

Similarly, this approach is applied to all genuses that are present in X_abd_, and a list of genus groups is created, as shown in Figure 1 after the select taxa rank step. This is repeated for the three taxonomic levels identified.

When Figure 1 is examined, firstly, in the grouping component G, for all the groups of genus, we partition X_train_ into sub data denoted as sub_d_x_. Following the earlier example of Alistipes, this group yields sub_d_alistipes_ which is created from X_train_. The sub_d_alistipes_ contains the labels of the samples, but the feature space is restricted only to species within the Alistipes genus. This is applied to all different genera created in the prior step, so we have multiple subsets of data with a feature space specified by genus. Secondly, in the scoring step S, the generated sub_d is trained on a Random Forest classifier with 5-fold cross-validation with randomized stratified shuffling. Each sub_d is given a score equal to the mean of the accuracy over all foldings based on the prediction of the labels. Each sub_d is scored and then sorted based on the score. The top k groups with the highest score are used for the subsequent step. The value chosen for k is 10, but other values for k have been tested. Following the example of selecting genus as the taxonomic level, the top 10 genus groups that show strong discriminative ability are used to build the classification model. Thirdly, in the modeling component, the species from the top 10 genus groups are used to train a Random Forest model with 100-fold Monte Carlo Cross-Validation (MCCV). The top ranking set of species corresponding to the top ranked group is trained on X_train_ and then tested on X_test_. Then, the second set of species corresponding to the second highest scoring group is aggregated with the top scoring set of species; and then used to train and test the model. This process is repeated until all species in the top 10 ranked genus groups are aggregated; and used to train and test the classifier. This whole process is repeated 100 times, stratifying the initial X_abd_ and randomly splitting it into X_train_ and X_test_ without replacement. The classification performance metrics are determined as the average of the metrics obtained in 100 folds. Similarly, the top ranked groups and the top ranked species are retained for each run.

### 2.3 Implementation of microBiomeGSM

The microBiomeGSM tool utilizes the pre-existing biological knowledge of the assignment of the species into different taxonomic levels, such as genus, family, and order. Experiments with the microBiomeGSM tool were conducted on the open-source KNIME platform (Berthold et al., 2009). This platform can handle a wide range of data types and operations. The user can configure the number of iterations, the rank function, and the number of iterations for MCCV. All rows with missing values are removed within the workflow.

### 2.4 Application of feature selection and classifiers using metagenomic data

In metagenomics research, it is observed that in studies using taxonomic features, the number of observations used for training data is higher than the number of observations used for testing data. This situation is undesirable if studies are to produce more effective results, and researchers are proposing various methods of resolution, particularly feature selection methods. Although the process of feature selection in disease prediction problems based on metagenome data has not been well studied, the literature suggests that this process may be as important as the choice of a classification method (LaPierre et al., 2019). The process of feature selection in metagenome-based disease prediction could help us learn more about disease development mechanisms. Therefore, further research in this direction is warranted. In metagenomics studies, in order to reduce the number of taxa, i.e., to select informative species (features), min Redundancy Max Relevance (mRMR) (Ding and Peng, 2005), Lasso (Tibshirani, 1996), Elastic Net (Zou and Hastie, 2005), and the iterative sure select algorithm (Duvallet et al., 2017) have been used extensively. Another feature selection method, called Fizzy, addresses the challenge of using classification techniques to identify important functional elements for downstream analysis (Ditzler et al., 2015). Oudah and Henschel presented an alternative taxonomy-based method for feature selection (Oudah and Henschel, 2018). Bakir-Gungor et al. (2021) applied CMIM (Fleuret and Ch, 2004), FCBF (Senliol et al., 2008), mRMR (Ding and Peng, 2005), and Select K best (SKB) (Pedregosa et al., 2011) to type 2 diabetes-associated metagenomics datasets and obtained powerful performance metrics (Bakir-Gungor et al., 2021). Jabeer et al. also proposed a robust classification method for evaluating colorectal cancer associated metagenomic datasets using a combination of feature selection methods and machine learning methods (Jabeer et al., 2022). Bakir-Gungor et al. (2022) also proposed a powerful method for IBD classification with fewer features by combining feature selection methods and machine learning methods (Bakir-Gungor et al., 2022). While these feature selection approaches have produced effective results in a variety of fields, they have only recently been applied to microbiome-based disease prediction problems.

In this study, we have comparatively evaluated microBiomeGSM with different classifiers and with different feature selection methods. As the feature selection methods, we have utilized Select K best (SKB), Fast Correlation Based Filter (FCBF), Extreme Gradient Boosting (XGBoost), Min Redundancy Max Relevance (mRMR), Information Gain (IG), and Conditional Mutual Information Maximization (CMIM). Wang and Liu (2020) compare the performance of classifiers with traditional methods and ensemble methods for disease prediction based on human microbiome data. They use Elastic Network and SVM as traditional methods and Random Forest and Extreme Gradient Boosting (XGBoost) as ensemble methods. In their study, they find that the XGBoost algorithm shows superior performance compared to other algorithms (Wang and Liu, 2020). In another study, Marcos-Zambrano et al. (2021) conducted an important review paper to reveal the links between the microbiome and diseases. In this study, which included information on the performance of machine learning methods, they found that the Support Vector Machines (SVM), Random Forest (RF), k-Nearest Neighbors (k-NN), and Logical Regression (LR) algorithms were widely used. They concluded that when selecting a machine learning algorithm, several factors should be considered such as the set of observations, the set of features, the type of data, and the quality of the data. They suggest using several different methods, comparing them, and choosing the one that provides the best performance value (Marcos-Zambrano et al., 2021).

### 2.5 microBiomeGSM model performance evaluation

Accuracy, F1 score, sensitivity, specificity, and AUC were used to evaluate the predictive performance of the proposed models. AUC score is a common measure for performance evaluation and a reliable metric for evaluating balanced datasets. Other metrics such as F1 score, sensitivity, specificity, and accuracy, were used to evaluate the performance of the created models because the dataset for this study has an uneven distribution of classes. When a balance between precision and recall is desired and there is an uneven distribution of classes, the F1 score is a good option among the performance metrics (many true negatives). Several classifiers report the probability values for their predictions, which can also be considered as confidence values for the prediction. The AUC often uses this information to figure out how often incorrect predictions occur at different confidence levels. In real life, test results from positive and negative examples overlap. AUC illustrates how the threshold or cut-off value for identifying positive examples affects the relationship between recall and precision. In this study, all of the above-mentioned metrics were calculated as the mean of 100 times MCCV. After each iteration, we obtain lists of significant taxonomic groups and species associated with these taxa groups for a given disease. To assign scores to the entities in the taxonomic groups list and in the species lists, a prioritization approach is used. For this purpose, we integrated the RobustRankAggreg algorithm (Kolde et al., 2012) and microBiomeGSM. RobustRankAggreg algorithm is available as an R package. Each entity (taxonomic group or species) in the lists is given a value of p by the RobustRankAggreg technique, indicating how highly ranked that entity. Using the RobustRankAggreg tool, microBiomeGSM outputs a list of species to which it has assigned a significance value (value of *p*) for a specific taxonomic group. Each taxa group is assigned a significance value and the species associated with that group are assigned the same value.

## 3 Results

The main objective of this study is to identify the microbial communities that are associated with specific diseases. In order to facilitate disease diagnosis, using metagenomic data we develop an efficient classification model based on taxonomic levels. In this section we present our findings for four different datasets. Here we also present comparative evaluation results against other existing methods.

### 3.1 Comparing varying group size for microBiomeGSM

One approach to evaluate model performance in the context of microBiomeGSM is to compare model performance between different values of the parameter k. k represents the number of groups (taxa) used in microBiomeGSM models. This approach can help researchers determine the optimal value of k that balances model complexity and predictive power, ultimately leading to more effective and interpretable models in microbiome-related research. It provides insight into how the inclusion or exclusion of specific taxa affects the overall performance of microBiomeGSM models.

Supplementary Table S2 shows the performance metrics obtained with 100-fold MCCV for the aggregated top 10 groups for four different datasets compared at three different taxonomic levels (genus, family, order) for grouping. For the IBDMDB dataset, microBiomeGSM achieved an AUC of 93% using the top 1 group at the family level. Performance metrics are shown for the top 2 groups via combining species from the first and second highest scoring groups. We obtained an AUC of 97% when the top 2 groups are combined at the family taxonomic level for the IBDMDB dataset. In this way, microBiomeGSM provides cumulative performance results for the top 10 highest scoring groups. For the IBDMDB dataset, the highest performance metric (an AUC of 98%) is obtained using the species from the top 10 groups at the order taxonomic level. For the IBD dataset, the highest performance metric (an AUC of 93%) is obtained using the species from the top 9 groups at the order taxonomic level. For the T2D dataset, the highest performance metric (an AUC of %74) is obtained using the species from the top 9 groups at the order taxonomic level. For the CRC dataset, the highest performance metric (an AUC of %83) is obtained using the species from the top 10 groups at the family taxonomic level. While examining other performance metrics (such as accuracy, sensitivity, specificity in Supplementary Table S2), it is noteworthy that satisfactory results are obtained with microBiomeGSM for each taxonomic level, especially for the IBDMDB dataset. The high sensitivity values that are reported for the CRC, IBDMDB, and IBD datasets display the success of the microBiomeGSM tool in terms of detecting the patient samples. In the CRC, IBDMDB, and IBD datasets, the strikingly high specificity values indicate that the microBiomeGSM tool correctly identifies the negative samples (i.e., individuals who do not have the disease). However, in the T2D dataset, the specificity rate appears to be relatively low compared to the other datasets. Nevertheless, the ability to detect negative samples remains at a reasonable level.

**Figure 2:**
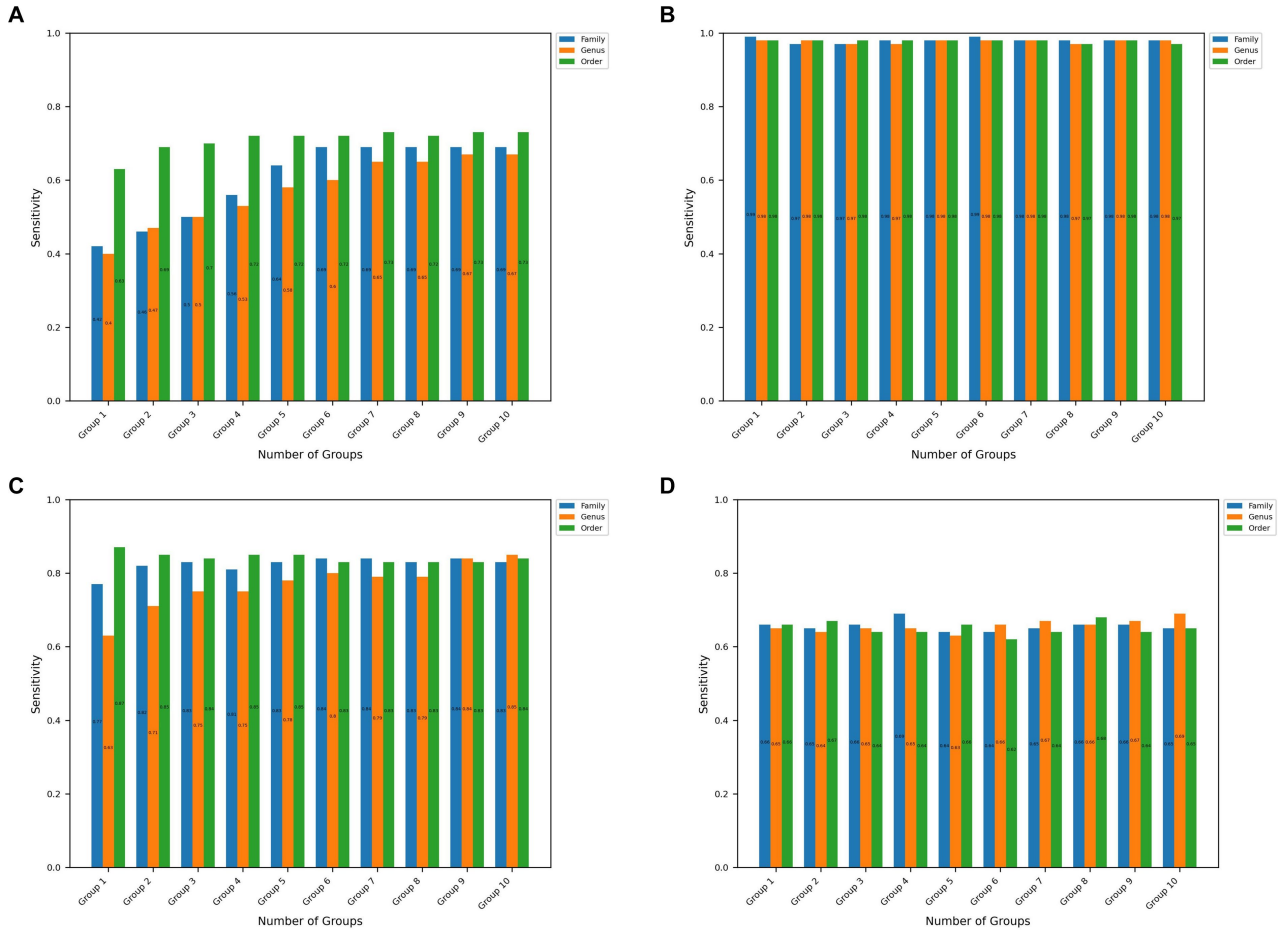
Sensitivity values obtained at the family, order, and genus taxon levels for the top 10 significant groups across all 4 datasets. **(A–D)** Represents the results obtained in CRC, IBDMDB, IBD, T2D datasets, respectively.

**Figure 3:**
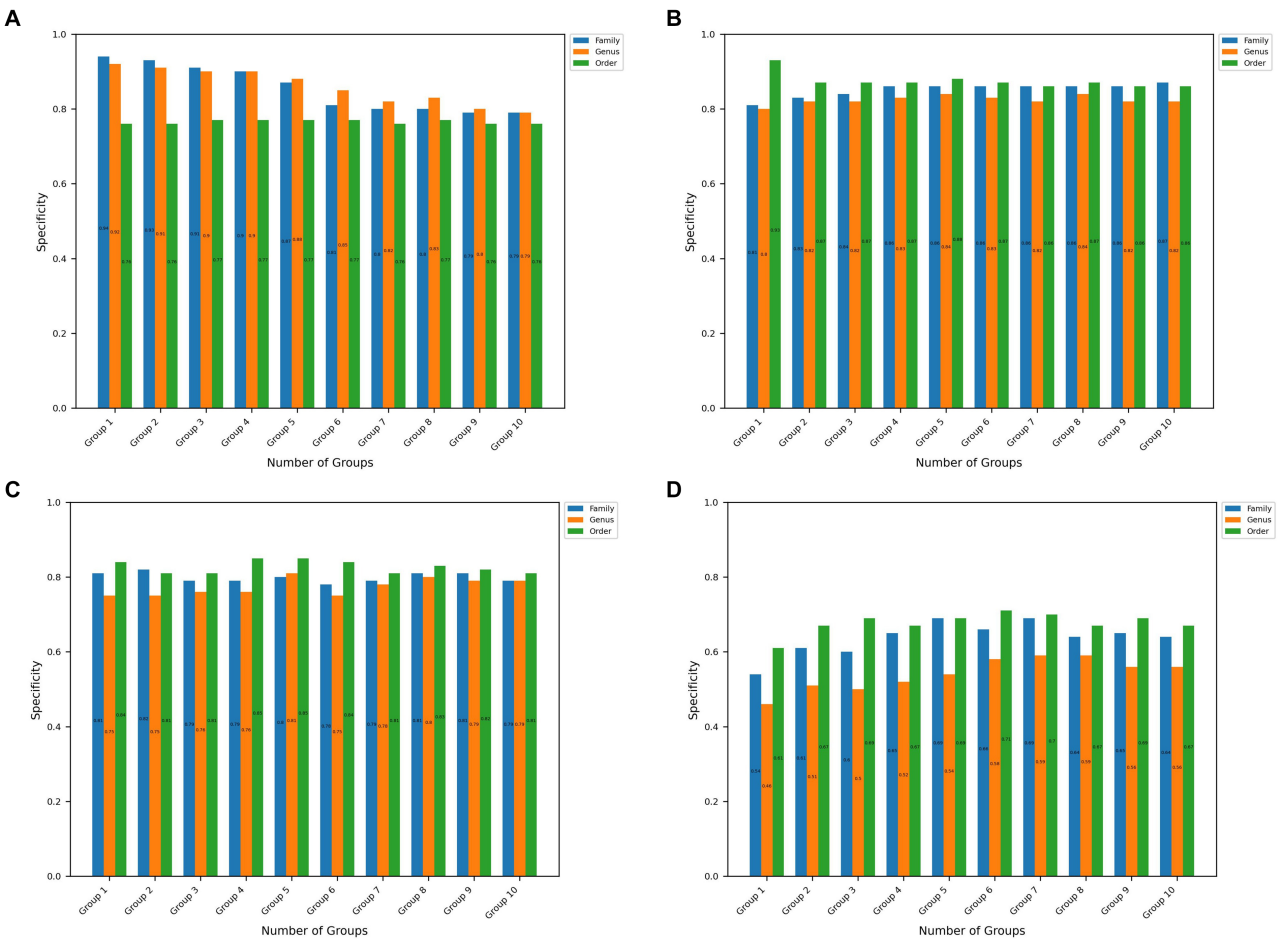
Specificity values at the order, genus, and family taxon level for the top 10 significant groups for all 4 disease datasets. **(A–D)** Represents the results obtained in CRC, IBDMDB, IBD, T2D datasets, respectively.

In addition, Figures 2, 3 show the sensitivity and specificity values obtained with the microBiomeGSM tool for all datasets. Figure 2 shows the sensitivity values obtained using the microBiomeGSM tool across all datasets. One can notice from Figure 2A that for the CRC data set the highest sensitivity value (73%) is obtained for the order taxon level using 10 cumulative groups. In particular, the sensitivity values calculated for the IBDMDB dataset were quite impressive, especially in group 1 and group 6, both at the family taxon level, reaching 99% sensitivity value, as shown in Figure 2B. Figure 2C shows another impressive set of results for the IBD data set. In Figure 2C, we observe high values for sensitivity, in particular 87% sensitivity at the taxon level in group 1. As shown in Figure 2D, the highest sensitivity value for the T2D data set is 69%. This result is obtained for the genus taxon level using 10 cumulative groups. A sensitivity value of 69% is also obtained for the family taxon level using 4 cumulative groups.

Figure 3 shows the specificity values obtained using the microBiomeGSM tool for all datasets. As shown in Figure 3A, the specificity value obtained for the CRC dataset is remarkable, reaching an impressive specificity value of 94% at the family taxon level for 1 group. Figure 3B depicts that the highest specificity value obtained for the IBDMDB dataset is 93% for 1 group at the order taxon level. As displayed in Figure 3C, the highest specificity value obtained for the IBD dataset is 85% for the 4 cumulative groups at the order taxon level. The same result is also obtained at the order taxon level for the 5 cumulative groups. One can notice in Figure 3D that the highest specificity value that is obtained for the T2D dataset is 71% for the 6 cumulative groups at the order taxon level.

The number of significant groups used to train the model could affect the performance of microBiomeGSM. Table 3 shows the influence of the number of groups and the number of species at family, genus and order levels on four datasets. Table 3 presents the performance of the top 10 cumulative groups and top 1 group for each taxonomic level on different tested datasets. For the IBDMDB dataset, for the family taxonomic level, one can observe that the AUC increases by 5% when we consider the top 10 significant groups cumulatively, while we increase the number of species from 34 to 205. On the same dataset, an increase of 8% in AUC score is observed at the Genus taxonomic level via increasing the number of species from 34 to 119. For the same dataset, a decrease of 1% is observed at the Order taxonomic level. Order taxonomic level using the top group that includes 98 species achieves the highest AUC success rate of 98% for the IBDMDB dataset. Similarly, family taxonomic level using the top 10 combined groups achieves 97% AUC on the IBDMDB dataset, but these 10 combined groups include a much higher number of species (205 species). For the IBD dataset, the highest AUC value of 91% was obtained using the microBiomeGSM tool. This value at the family taxonomic level was obtained by cumulatively combining 10 groups, using an average of 260.4 species. For the T2D dataset, the highest AUC value of 72% was obtained using the microBiomeGSM tool. This value, obtained at the order taxanomic level, was obtained by combining 10 groups cumulatively. For 1 group, an average of 138.28 species are used at the taxonomic level, while for 10 groups, an average of 596.99 species are used. For the CRC dataset, the highest AUC value of 87% was obtained using the microBiomeGSM tool. This value at the order taxanomic level was obtained by cumulatively combining 10 groups, using an average of 604 species.

**Table 3:** The effect of the number of groups that are generated at different taxonomic levels on performance metrics for all dataset.

CRC								
Taxonomic hierarchy	# of groups	Average # of species	Accuracy	Sen	Spe	F measure	AUC	Precision
Family	10	239.11	0.78	0.84	0.72	0.79	0.84	0.76
Family	1	16.17	0.67	0.91	0.43	0.73	0.69	0.62
Genus	10	102.06	0.77	0.82	0.71	0.78	0.84	0.76
Genus	1	7.16	0.66	0.88	0.44	0.72	0.71	0.63
Order	10	604	0.82	0.86	0.78	0.82	**0.87**	0.81
Order	1	154.25	0.76	0.82	0.71	0.78	0.81	0.76

IBDMDB								
Taxonomic hierarchy	# of Groups	Average # of species	Accuracy	Sen	Spe	F measure	AUC	Precision
Family	10	205.76	0.95	0.98	0.87	0.95	0.97	0.93
Family	1	34	0.93	0.98	0.81	0.94	0.93	0.9
Genus	10	119.4	0.92	0.98	0.82	0.95	0.97	0.93
Genus	1	34	0.92	0.98	0.80	0.94	0.91	0.91
Order	10	341.22	0.93	0.97	0.86	0.95	**0.98**	0.93
Order	1	98	0.96	0.98	0.93	0.97	0.98	0.95

IBD								
Taxonomic hierarchy	# of Groups	Average # of species	Accuracy	Sen	Spe	F measure	AUC	Precision
Family	10	260.24	0.82	0.85	0.79	0.82	**0.91**	0.81
Family	1	51.59	0.78	0.78	0.78	0.78	0.86	0.78
Genus	10	121.78	0.81	0.83	0.79	0.81	0.88	0.8
Genus	1	12.26	0.7	0.67	0.74	0.69	0.78	0.73
Order	10	608.27	0.82	0.82	0.81	0.82	0.9	0.82
Order	1	174.86	0.81	0.82	0.8	0.81	0.9	0.81

T2D								
Taxonomic hierarchy	# of groups	Average # of species	Accuracy	Sen	Spe	F measure	AUC	Precision
Family	10	321.16	0.65	0.68	0.63	0.66	0.71	0.65
Family	1	39.86	0.59	0.71	0.47	0.63	0.63	0.58
Genus	10	129.8	0.64	0.64	0.64	0.64	0.69	0.65
Genus	1	15.94	0.56	0.62	0.49	0.58	0.58	0.55
Order	10	596.99	0.65	0.65	0.64	0.64	**0.72**	0.65
Order	1	138.28	0.59	0.67	0.52	0.62	0.64	0.59

The results in bold in table represent the best AUC results.

microBiomeGSM reports important groups of features that are detected at different taxonomic levels for the disease under study. Table 4 lists the top 10 important groups that are identified by microBiomeGSM for three different taxonomic levels on four different datasets. The identified features are ranked by their importance scores from high to low. The feature with the highest importance value is the strongest candidate to be announced as potential taxonomic biomarker for the disease under investigation.

**Table 4:** Top 10 groups identified by microBiomeGSM for different taxonomic levels, applied on all microbiome datasets.

CRC			
#	Taxonomic levels		
Rank	Family	Order	Genus
1	PEPTOSTREPTOCOCCACEAE	CLOSTRIDIALES	PARVIMONAS
2	PEPTONIPHILACEAE	TISSIERELLALES	PEPTOSTREPTOCOCCUS
3	FUSOBACTERIACEAE	BACTEROIDALES	FUSOBACTERIUM
4	BACILLALES_UNCLASSIFIED	FUSOBACTERIALES	GEMELLA
5	VEILLONELLACEAE	BACILLALES	DIALISTER
6	LACHNOSPIRACEAE	VEILLONELLALES	LACHNOCLOSTRIDIUM
7	ERYSIPELOTRICHACEAE	ERYSIPELOTRICHALES	PREVOTELLA
8	RUMINOCOCCACEAE	LACTOBACILLALES	STREPTOCOCCUS
9	PREVOTELLACEAE	ACTINOMYCETALES	PORPHYROMONAS
10	STREPTOCOCCACEAE	DESULFOVIBRIONALES	SOLOBACTERIUM

IBDMDB			
#	Taxonomic levels		
Rank	Family	Order	Genus
1	BACTEROIDACEAE	BACTEROIDALES	BACTEROIDES
2	LACHNOSPIRACEAE	CLOSTRIDIALES	ALISTIPES
3	RUMINOCOCCACEAE	FIRMICUTES_UNCLASSIFIED	EUBACTERIUM
4	RIKENELLACEAE	VEILLONELLALES	ROSEBURIA
5	FIRMICUTES_UNCLASSIFIED	BURKHOLDERIALES	FIRMICUTES_UNCLASSIFIED
6	TANNERELLACEAE	METHANOMASSILIICOCCALES	PARABACTEROIDES
7	EUBACTERIACEAE	DESULFOVIBRIONALES	RUMINOCOCCUS
8	CLOSTRIDIACEAE	ERYSIPELOTRICHALES	COPROCOCCUS
9	VEILLONELLACEAE	BIFIDOBACTERIALES	BLAUTIA
10	ODORIBACTERACEAE	EGGERTHELLALES	CLOSTRIDIUM

IBD			
#	Taxonomic levels		
Rank	Family	Order	Genus
1	LACHNOSPIRACEAE	CLOSTRIDIALES	BLAUTIA
2	BIFIDOBACTERIACEAE	CORIOBACTERIALES	BIFIDOBACTERIUM
3	CORIOBACTERIACEAE	BIFIDOBACTERIALES	EUBACTERIUM
4	RUMINOCOCCACEAE	ERYSIPELOTRICHALES	DOREA
5	ERYSIPELOTRICHACEAE	BACTEROIDALES	COLLINSELLA
6	CLOSTRIDIALES_FAMILY_XIII_INCERTAE_SEDIS	LACTOBACILLALES	PEPTOSTREPTOCOCCUS
7	EUBACTERIACEAE	SELENOMONADALES	COPROCOCCUS
8	PEPTOSTREPTOCOCCACEAE	VERRUCOMICROBIALES	ERYSIPELOTRICHACEAE_NONAME
9	CARNOBACTERIACEAE	CANDIDATUS_SACCHARIBACTERIA_NONAME	LACHNOSPIRACEAE_NONAME
10	CLOSTRIDIACEAE	BACILLALES	BACTEROIDES

T2D			
#	Taxonomic levels		
Rank	Family	Order	Genus
1	LACHNOSPIRACEAE	CLOSTRIDIALES	EUBACTERIUM
2	BIFIDOBACTERIACEAE	BIFIDOBACTERIALES	BIFIDOBACTERIUM
3	RUMINOCOCCACEAE	CORIOBACTERIALES	BLAUTIA
4	EUBACTERIACEAE	BACTEROIDALES	DOREA
5	CORIOBACTERIACEAE	LACTOBACILLALES	LACHNOSPIRACEAE_NONAME
6	CLOSTRIDIALES_FAMILY_XIII_INCERTAE_SEDIS	ERYSIPELOTRICHALES	RUMINOCOCCUS
7	ERYSIPELOTRICHACEAE	SELENOMONADALES	COPROCOCCUS
8	PEPTOSTREPTOCOCCACEAE	VERRUCOMICROBIALES	PEPTOSTREPTOCOCCUS
9	CARNOBACTERIACEAE	METHANOBACTERIALES	ERYSIPELOTRICHACEAE_NONAME
10	BACTEROIDACEAE	BACILLALES	GRANULICATELLA

The microBiomeGSM tool lists a number of associated species for each identified group. The species included in the top 5 significant groups are listed in Supplementary Tables S3–S5 for family, order, and genus taxonomic levels, respectively for four different datasets. All species for the family, order, and genus taxonomic levels for the T2D, IBDMDB and CRC datasets can be found in Supplementary Tables S6–S14, respectively.

For the IBDMDB dataset, the changes in the AUC score when the number of groups is increased from 1 to 10 are shown in Supplementary Figure S1. For the IBDMDB dataset, a high AUC score is obtained at the order taxonomic level. When the number of groups was increased, the AUC score decreased relatively, and no significant change was observed after 5 groups. At the genus and family taxonomic levels, there is a significant increase in the AUC score until 5 groups are combined and no significant change after 5 groups.

### 3.2 Comparing against traditional machine learning methods

Our Grouping-Scoring-Modeling (G-S-M) approach emerges as a paradigm shift from traditional feature selection methods. Instead of pinpointing individual informative features, the GSM methodology groups these features. These groups are then scored, and a classification model is built using these top-ranking feature conglomerates. The versatility of the GSM method, as detailed in our prior work (Yousef et al., 2021b), lies in its adaptability. Groups can be created either by computational/statistical methods or by using domain-specific knowledge. In order to use the GSM strategy for a given dataset, a deep domain expertise is required to skillfully define these groups, which makes each application different. The modifications required to tailor the G-S-M approach to the unique needs of microbiome research highlight the adaptability of the G-S-M method and the novelty of our current study.

We have comparatively evaluated the performance of microBiomeGSM against 4 different classifiers and 6 different feature selection methods using the same datasets. All algorithms are run with default parameters. The developed approach and feature selection methods were executed multiple times, and the results were averaged and shared. Table 5 shows the performance of the different feature selection algorithms and different classifiers on the same disease associated microbiome datasets. In these experiments, the number of features was set to 100. The best result for the IBDMDB dataset is obtained by using the XGBoost feature selection algorithm in combination with the Random Forest classification algorithm with 98% AUC. For the CRC dataset, the best result is obtained by using the XGBoost feature selection algorithm in combination with the Random Forest classification algorithm with an AUC of 85%. For the IBD dataset, the best result is obtained using the Random Forest classification algorithm with 92% AUC and the SKB feature selection algorithm. For the T2D dataset, the best result is obtained by using the XGBoost feature selection algorithm in combination with the Random Forest classification algorithm with 70% AUC.

**Table 5:** Area under the curve (AUC) results obtained using 100 features for different feature selection methods and classifiers for all dataset.

CRC						
Model	SKB	IG	XGB	FCBF	MRMR	CMIM
Adaboost	0.75 ± 0.02	0.71 ± 0.05	0.78 ± 0.04	0.71 ± 0.05	0.63 ± 0.06	0.77 ± 0.04
DT	0.67 ± 0.04	0.64 ± 0.04	0.69 ± 0.04	0.63 ± 0.06	0.61 ± 0.04	0.65 ± 0.05
Logitboost	0.76 ± 0.04	0.72 ± 0.05	0.78 ± 0.06	0.70 ± 0.04	0.64 ± 0.06	0.76 ± 0.05
RF	0.82 ± 0.03	0.79 ± 0.04	**0.85 ± 0.03**	0.77 ± 0.05	0.74 ± 0.04	0.80 ± 0.03

IBDMDB						
Model	SKB	IG	XGB	FCBF	MRMR	CMIM
Adaboost	0.89 ± 0.04	0.90 ± 0.03	0.89 ± 0.06	0.49 ± 0.08	0.51 ± 0.08	0.51 ± 0.08
DT	0.83 ± 0.03	0.82 ± 0.04	0.84 ± 0.03	0.46 ± 0.07	0.50 ± 0.07	0.50 ± 0.06
Logitboost	0.89 ± 0.04	0.91 ± 0.03	0.86 ± 0.06	0.50 ± 0.06	0.51 ± 0.08	0.49 ± 0.08
RF	0.96 ± 0.01	0.96 ± 0.01	**0.98 ± 0.01**	0.46 ± 0.1	0.54 ± 0.08	0.52 ± 0.07

IBD						
Model	SKB	IG	XGB	FCBF	MRMR	CMIM
Adaboost	0,90 ± 0.07	0,89 ± 0.03	0,91 ± 0.03	0,51 ± 0.06	0,51 ± 0.03	0,66 ± 0.08
DT	0,78 ± 0.08	0,70 ± 0.08	0,73 ± 0.07	0,53 ± 0.08	0,51 ± 0.04	0,56 ± 0.09
Logitboost	0,90 ± 0.04	0,90 ± 0.05	0,92 ± 0.05	0,55 ± 0.1	0,53 ± 0.05	0,59 ± 0.1
RF	**0,92 ± 0.03**	0,88 ± 0.06	0,91 ± 0.04	0,53 ± 0.09	0,55 ± 0.07	0,63 ± 0.11

T2D						
Model	SKB	IG	XGB	FCBF	MRMR	CMIM
Adaboost	0,56 ± 0.12	0,60 ± 0.05	0,64 ± 0.07	0,50 ± 0.10	0,5 ± 0.01	0,50 ± 0.12
DT	0,52 ± 0.08	0,52 ± 0.08	0,53 ± 0.05	0,41 ± 0.10	0,51 ± 0.02	0,49 ± 0.10
Logitboost	0,55 ± 0.10	0,58 ± 0.09	0,62 ± 0.10	0,48 ± 0.08	0,50 ± 0.01	0,51 ± 0.11
RF	0,62 ± 0.11	0,62 ± 0.07	**0,70 ± 0.06**	0,49 ± 0.08	0,51 ± 0.03	0,54 ± 0.10

The results in bold in table represent the best AUC results for the respective disease (CRC, IBDMDB, IBD, T2D).

We would like to note that the primary objective of microBiomeGSM is not to compete with other feature selection methods (FS). Even if microBiomeGSM’s performance is on par with or slightly less favorable than other FS methods, its fundamental contribution lies in identifying the most informative microbiomes. These microbiomes play a pivotal role in aiding researchers in gaining a deeper understanding of the biological underpinnings of the disease under investigation. In essence, microBiomeGSM’s value lies in its ability to contribute to the advancement of biological knowledge, rather than merely outperforming other feature selection techniques.

Table 6 shows the performance metrics of microBiomeGSM for each taxonomic level for four different datasets. The # of species column shows the number of species (features/variables) used to train and test the model. Since the number of species changes in each iteration of MCCV, we also report the standard deviation. Performance metrics are reported as the average of 100 iterations with the corresponding standard deviation. For the CRC dataset, among different classifiers the RF algorithm has the highest performance for all calculated metrics including the accuracy, sensitivity, specificity, precision, and AUC metric. The AdaBoost, LogitBoost and DT models show lower performance compared to the RF model. The performance metrics of these three algorithms are similar but not as high as RF model. At the order taxonomic level, the mean values of the performance metrics are stable and the standard deviations are low. This indicates that the order level is a more appropriate choice for CRC classification. Comparing the RF model and the microBiomeGSM model, similar performance metrics are obtained for the CRC dataset, but it is worth mentioning that the number of features used in the proposed tool is lower. In other words, for the CRC dataset the microBiomeGSM model can accurately classify using fewer taxonomic features. For the IBDMDB dataset, among different classifiers the RF algorithm has the highest accuracy, sensitivity, specificity, precision, and AUC values. In particular, RF model achieved very high sensitivity and AUC values. For the IBDMDB dataset, the microBiomeGSM tool achieves an AUC of 98% for the order taxon level, the same performance metrics as obtained by the RF classification algorithm. However, the microBiomeGSM tool uses 341 features for the order taxon level, while the RF model uses 579 features. For IBD dataset, the RF algorithm generates the highest performance on several metrics, including accuracy, sensitivity, specificity, precision, and AUC. It performs particularly well on sensitivity and AUC. In our analysis, microBiomeGSM achieved an impressive AUC value of 91% at the family taxon level. Equally remarkable is the similar performance of the RF classification algorithm (an AUC of 92%) for the same task. However, it is important to highlight an important difference between these two approaches. For IBD dataset the RF classification algorithm achieved an AUC of 92% by using a much larger set of features (1,456 features) for the classification task. For the same dataset, the microBiomeGSM tool also showed remarkable performance (an AUC value of 91%). In stark contrast, microBiomeGSM achieved nearly equivalent AUC performance while using a much smaller set of features, only 260 features. This divergence in feature usage highlights the effectiveness and potential advantages of the microBiomeGSM tool in extracting meaningful information from microbiome data while optimizing computational resources. For T2D dataset, the RF classification algorithm outperforms other classification algorithms on several performance metrics including accuracy, sensitivity, specificity, precision and AUC. microBiomeGSM achieved an AUC value of 72% at the order taxon level. Interestingly, a similar level of performance is observed using the RF classification algorithm, which achieves an AUC value of 75%. However, it is important to note that the underlying mechanisms of these two methods are very different. The RF classification algorithm achieves this AUC value by incorporating a much larger set of features, 1,456 features, into its classification process. In contrast, the microBiomeGSM tool achieves comparable AUC metric by using a leaner set of 596 features. This difference in feature usage is worth highlighting as it shows that the microBiomeGSM tool is able to deliver competitive results with a lower computational load, making it an efficient and resource-efficient choice for the classification task at hand. These results highlight the nuanced trade-offs in selecting the appropriate tool or algorithm for the specific data analysis requirements.

**Table 6:** Evaluation metrics obtained with microBiomeGSM on four datasets for different taxonomic levels, compared with traditional classifiers using all features.

CRC						
Model	# of Species	Accuracy	Sensitivity	Specificity	Precision	AUC
AdaBoost	912	0.72 ± 0.06	0.79 ± 0.09	0.66 ± 0.17	0.7 ± 0.09	0.78 ± 0.04
DT	912	0.68 ± 0.09	0.75 ± 0.12	0.62 ± 0.26	0.66 ± 0.09	0.7 ± 0.04
LogitBoost	912	0.73 ± 0.06	0.78 ± 0.09	0.68 ± 0.18	0.71 ± 0.09	0.78 ± 0.04
RF	912	0.78 ± 0.05	0.82 ± 0.08	0.75 ± 0.14	0.76 ± 0.09	0.86 ± 0.03
microBiomeGSM: family	292.88 ± 16.09	0.74 ± 0.65	0.7 ± 0.39	0.77 ± 0.91	0.75 ± 0.83	0.81 ± 0.67
microBiomeGSM: genus	161.21 ± 5.17	0.74 ± 0.67	0.69 ± 0.41	0.79 ± 0.92	0.76 ± 0.84	0.8 ± 0.68
microBiomeGSM: order	607.5 ± 188.32	0.73 ± 0.69	0.72 ± 0.66	0.75 ± 0.73	0.74 ± 0.71	0.81 ± 0.77

IBDMDB						
Model	# of Species	Accuracy	Sensitivity	Specificity	Precision	AUC
AdaBoost	579	0.92 ± 0.02	0.97 ± 0.02	0.79 ± 0.1	0.93 ± 0.03	0.94 ± 0.01
DT	579	0.91 ± 0.02	0.94 ± 0.01	0.84 ± 0.05	0.94 ± 0.02	0.89 ± 0.02
LogitBoost	579	0.92 ± 0.01	0.98 ± 0.01	0.76 ± 0.07	0.92 ± 0.02	0.91 ± 0.04
RF	579	0.98 ± 0.01	1 ± 0	0.93 ± 0.06	0.98 ± 0.02	0.98 ± 0.01
microBiomeGSM: Family	205.76 ± 16.23	0.94 ± 0.02	0.98 ± 0.01	0.86 ± 0.05	0.93 ± 0.05	0.97 ± 0.02
microBiomeGSM: Genus	119.4 ± 15.87	0.93 ± 0.02	0.98 ± 0.01	0.85 ± 0.05	0.93 ± 0.05	0.97 ± 0.02
microBiomeGSM: Order	341.22 ± 15.6	0.93 ± 0.02	0.97 ± 0.02	0.86 ± 0.06	0.93 ± 0.06	0.98 ± 0.03

IBD						
Model	# of Species	Accuracy	Sensitivity	Specificity	Precision	AUC
AdaBoost	1,456	0.88 ± 0.04	0.85 ± 0.12	0.89 ± 0.05	0.84 ± 0.05	0.9 ± 0.04
DT	1,456	0.75 ± 0.05	0.72 ± 0.09	0.78 ± 0.06	0.67 ± 0.08	0.75 ± 0.06
LogitBoost	1,456	0.85 ± 0.04	0.81 ± 0.1	0.87 ± 0.07	0.8 ± 0.09	0.88 ± 0.04
RF	1,456	0.87 ± 0.05	0.91 ± 0.1	0.84 ± 0.05	0.78 ± 0.06	0.92 ± 0.05
microBiomeGSM: Family	260.24 ± 26.92	0.82 ± 0.06	0.85 ± 0.07	0.79 ± 0.1	0.81 ± 0.13	0.91 ± 0.07
microBiomeGSM: Genus	121.78 ± 27.83	0.81 ± 0.06	0.83 ± 0.06	0.79 ± 0.1	0.8 ± 0.12	0.88 ± 0.08
microBiomeGSM: Order	608.27 ± 24.22	0.82 ± 0.07	0.82 ± 0.08	0.81 ± 0.09	0.82 ± 0.15	0.9 ± 0.08

T2D						
Model	# of Species	Accuracy	Sensitivity	Specificity	Precision	AUC
AdaBoost	1,456	0.68 ± 0.08	0.91 ± 0.08	0.39 ± 0.26	0.67 ± 0.09	0.66 ± 0.1
DT	1,456	0.57 ± 0.05	0.98 ± 0.06	0.06 ± 0.19	0.57 ± 0.06	0.57 ± 0.09
LogitBoost	1,456	0.67 ± 0.08	0.93 ± 0.08	0.36 ± 0.24	0.65 ± 0.08	0.65 ± 0.1
RF	1,456	0.72 ± 0.09	0.91 ± 0.09	0.48 ± 0.29	0.71 ± 0.12	0.75 ± 0.1
microBiomeGSM: family	321.16 ± 36.31	0.65 ± 0.08	0.68 ± 0.09	0.63 ± 0.11	0.65 ± 0.15	0.71 ± 0.08
microBiomeGSM: genus	129.8 ± 35.03	0.64 ± 0.09	0.64 ± 0.1	0.64 ± 0.13	0.65 ± 0.18	0.69 ± 0.09
microBiomeGSM: order	596.99 ± 35.14	0.65 ± 0.08	0.65 ± 0.09	0.64 ± 0.12	0.65 ± 0.17	0.72 ± 0.09

As shown in Table 7, the performance of our proposed method varies depending on the taxonomic level considered. For the order taxonomic level, for all tested datasets, the proposed method outperforms other models in terms of the AUC score, except for the RF classifier. Similarly, for all datasets, at the family and genus taxonomic levels, the AUC values are also highly competitive, outperforming those of the other four machine learning algorithms used in this study, with the sole exception of the RF classifier. These results highlight the robust performance of our method across different taxonomic levels. A remarkable performance of our proposed method was observed when it is applied on the IBDMDB dataset. Here, we obtained an exceptionally high AUC value of 0.98 ± 0.03 at the order taxonomic level using a 100-fold MCCV approach. This remarkable result demonstrates the exceptional performance and the potential of the microBiomeGSM tool.

**Table 7:** Comparative performance evaluation of microBiomeGSM and other machine learning approaches for different microbiome datasets.

Dataset		AdaBoost	DT	LogitBoost	RF	microBiomeGSM: family	microBiomeGSM: genus	microBiomeGSM: order
CRC	AUC	0.78 ± 0.14	0.70 ± 0.04	0.78 ± 0.04	**0.86** ± 0.03	0.81 ± 0.67	0.80 ± 0.68	**0.81** ± 0.77
	# of Species	912	912	912	912	292.88 ± 16.09	161.21 ± 5.17	607.5 ± 188.32
IBDMDB	AUC	0.94 ± 0.01	0.89 ± 0.02	0.91 ± 0.04	**0.98** ± 0.01	0.97 ± 0.02	0.97 ± 0.03	**0.98 ± 0.03**
	# of Species	579	579	579	579	205.76 ± 16.23	119.4 ± 15.87	341.22 ± 15.6
IBD	AUC	0.9 ± 0.04	0.75 ± 0.06	0.88 ± 0.04	**0.92** ± 0.05	**0.91** ± 0.07	0.88 ± 0.08	0.9 ± 0.08
	# of Species	1,456	1,456	1,456	1,456	260.24 ± 26.92	121.78 ± 27.83	608.27 ± 24.22
T2D	AUC	0.66 ± 0.1	0.57 ± 0.09	0.65 ± 0.1	**0.75** ± 0.1	0.71 ± 0.08	0.69 ± 0.09	**0.72** ± 0.09
	# of Species	1,456	1,456	1,456	1,456	321.16 ± 36.31	129.8 ± 35.03	596.99 ± 35.14

The results in bold in table represent the best AUC result among the 4 different traditional classifiers (AdaBoost, DT, LogitBoost and RF) and the best AUC result across taxon levels using microBiomeGSM.

## 4 Discussion

The microbiome is considered as a crucial component of the human body and it is increasingly associated with numerous aspects of development and health. There is growing evidence that the microbiota is essential for understanding, diagnosing, and treating human diseases. In particular, alterations in the gut microbiome community have been linked to a variety of diseases, including CRC (Song et al., 2020), T2D (Salamon et al., 2018) and IBD (Alam et al., 2020). Several research efforts relied on sample-level feature abundance data to identify predictive microbiome biomarkers using machine learning. In this study, we proposed to perform more effective disease classification and prediction with fewer features. To this end, we developed microBiomeGSM to solve this problem compared to tools that perform predictions with a large amount of data. The success of microBiomeGSM can be explained with the following features of the G-S-M approach:

- For the grouping component of microBiomeGSM, only the features at the similar taxonomic levels are considered.
- microBiomeGSM uses efficient classifiers for the scoring component to identify the key groups for each taxonomic level;
- For the modeling component, significant taxonomic groups are considered cumulatively using effective classifiers.

Via analyzing metagenomic data, this study aims to solve the problem of disease diagnosis using existing taxonomic knowledge; and finally introduces a tool called microBiomeGSM. The proposed tool is based on the G-S-M (Grouping-Scoring-Modeling) approach and uses species-level information by grouping taxonomic features at different taxonomic levels such as genus, family, and order. The performance of microBiomeGSM on four different disease-associated metagenomic datasets was evaluated in comparison to other feature selection methods such as Fast Correlation Based Filter (FCBF), Select Best K (SKB), Extreme Gradient Boosting (XGB), Conditional Mutual Information Maximization (CMIM), Maximum Likelihood and Minimum Redundancy (MRMR), and Information Gain (IG).

The presented microBiomeGSM approach offers several advantages in the field of disease diagnosis via analyzing metagenomic datasets. One significant benefit is its ability to efficiently identify disease-associated taxonomic biomarkers through a robust machine learning model based on the Grouping, Scoring, and Modeling (G-S-M) methodology. Differently from existing approaches, microBiomeGSM identifies groups of important taxons and detects important species within that taxon for the disease under study. Hence, this innovative approach enables the extraction of valuable insights from microbiome data, shedding light on the influence of specific taxonomic biomarkers on the disease under investigation. Furthermore, the performance evaluation across different diseases, different taxonomic levels (genus, family, order); and the comparative assessment with different feature selection algorithms exhibits the reliability of microBiomeGSM. Finally, the discussions on the biological relevance of the findings of the proposed approach, via drawing evidence from the existing literature, provide valuable context for the identified taxon groups for the disease under study, making microBiomeGSM an informative tool in disease research. Our tool’s significance transcends its mere application; it holds the potential for pioneering discoveries. It is geared to discern not isolated microbial entities but entire assemblages of species, paving the way for profound biological interpretations. By spotlighting groups of bacteria and viruses in lieu of singular entities, our tool offers a holistic view, potentially identifying microbial communities implicated in specific diseases.

With this study, we would also like to motivate biologists and the microbiome community to redesign their grouping methods instead of using individual feature selection approaches. We envision that in the future, various biological datasets, including multi-omics, will be used to redefine the groupings. Such innovative grouping strategies, complemented by modeling, promise to provide profound insights into the molecular mechanisms of diseases and the role of microorganisms in disease development.

### 4.1 Biological interpretations of microBiomeGSM’s findings

This section discusses the biological relevance of the features discovered by microBiomeGSM at different taxonomic levels for all tested datasets. T2D is a metabolic disease characterized by high glucose levels in blood and caused primarily by cellular resistance to the activity of insulin (Sedighi et al., 2017). There are several studies in the literature that have demonstrated the relation of different microorganisms at the genus, family, and order levels with T2D development. For the T2D dataset, the top 10 microbiomes identified by our method at the genus, family, order levels and the relevant literature can be summarized in Supplementary Table S15. On the other hand, inflammatory bowel diseases (IBDs), which include primarily ulcerative colitis and Crohn’s disease, but also non-infectious inflammation of the bowel, have puzzled gastroenterologists and immunologists alike since their first modern descriptions around some 75–100 years ago (Ni et al., 2018; Bakir-Gungor et al., 2022). For the IBDMDB dataset, the top 10 microbiomes identified by our method at the genus, family, and order levels and the relevant literature can be summarized in Supplementary Table S15. CRC is a prevalent malignancy affecting the colon and rectum. It constitutes approximately 10% of all newly diagnosed cancer cases worldwide (Li X. et al., 2023). For the CRC dataset, the top 10 microbiomes identified by our method at the genus, family, and order levels and the relevant literature can be summarized in Supplementary Table S15.

Numerous studies have investigated the relationship between microbiomes and diseases like T2D, CRC, and IBD using similar datasets as used within this study. Upon examination of these studies, it becomes evident that while their experimental designs may vary, they consistently yield comparable results when it comes to identifying microbiomes linked to these diseases. These findings align with the important microbiomes identified by microBiomeGSM for T2D, CRC, and IBD, showcasing the tool’s effectiveness in accurately identifying relevant microbiomes associated with these diseases. These congruent findings reinforce the reliability and validity of the microbiome associations detected by the microBiomeGSM tool. It also underscores the tool’s capacity to identify microbiomes that are consistently linked to specific diseases, providing valuable insights for disease characterization and prediction. Hassouneh et al. (2021) conducted a series of experiments aimed at uncovering microbiomes associated with IBD. In their analysis using the same dataset as used by the microBiomeGSM tool, they observed differences in Clostridium microbiota among IBD patients. Additionally, another microbiome identified for IBD in their study is *Ruminococcus*. Remarkably, these microbiomes align with the important microbiomes detected for the IBD disease by the microBiomeGSM tool. This correspondence in findings highlights the capacity of microBiomeGSM in identifying relevant microbiomes linked to IBD. Zhang Y. et al. (2022) conducted a study with the goal of identifying disease-associated microbiome species for Inflammatory Bowel Disease Microbiome Database (IBDMDB), employing the same dataset (PRJNA289734) as used in microBiomeGSM. In their research, they highlighted the significance of the Bacteroides microbiome. Interestingly, the Bacteroides microbiome is also identified as one of the important microbiomes by the microBiomeGSM tool proposed in our study. This alignment in findings underscores the effectiveness of microBiomeGSM in recognizing key microbiomes associated with diseases like IBD. Bai et al. (2022) conducted a series of experiments aimed at identifying microbiomes associated with T2D. In their research, they utilized the SRA4565 data for T2D and highlighted the significance of the methanobacteriales microbiome. Notably, methanobacteriales is among the top 10 microbiomes identified by the proposed microBiomeGSM tool. This convergence of findings underscores the effectiveness and utility of the proposed tool in uncovering microbiome associations with diseases like T2D. Forslund et al. (2015) conducted experiments utilizing the same T2D dataset employed by microBiomeGSM to investigate microbiomes associated with T2D. Upon close examination of their experiments, they underscored the significance of the Clostridiales microbiome in relation to T2D disease. Interestingly, Clostridiales also emerges as one of the important microbiomes identified by microBiomeGSM. This convergence in findings highlights the relevance and effectiveness of microBiomeGSM in identifying crucial microbiomes associated with T2D. Ma et al. (2021) conducted a study that investigated the microbiomes associated with CRC using the same dataset as in our study. Among the various microbiomes they examined, the Prevotella microbiome stood out as strongly linked to CRC. This association aligns with the findings of microBiomeGSM, underscoring the significance of the Prevotella microbiome in the context of characterizing CRC. Chen et al. (2023) conducted research using the same dataset to investigate microbiomes in the context of colorectal cancer, akin to the proposed microBiomeGSM tool. Similar to the findings of microBiomeGSM, their study also identified Peptostreptococcus, Fusobacterium, and Porphyromonas microbiomes as valuable and effective biomarkers for CRC. This convergence in results underscores the potential significance of these specific microbiomes in CRC characterization and their importance as potential biomarkers for the disease.

In summary, via analyzing the raw microbiome data of specific diseases, this study aims to identify taxonomic biomarkers that may have a role in the associated diseases. Three different taxon levels (genus, family, and order) are studied and disease prediction is performed by building effective machine learning models using the G-S-M approach. Four different datasets are analyzed and the identified microorganisms at genus, family and order levels are compared with the existing literature.

### 4.2 Limitation of the study

The quality and the scope of our study have been significantly influenced by several primary limiting factors. These factors encompass the nature of the data set, the tools employed for data preprocessing, the specific taxon groups considered, and the overall volume of data under examination. First and foremost, the data set itself plays a pivotal role in shaping the outcomes and conclusions of our study. Its size, diversity, and representativeness directly impact the generalizability of our findings. Furthermore, the quality of data, its sources, and any potential biases within the dataset significantly affect the reliability of our results. Equally significant is the role of the tools employed for data preprocessing. The choices made in data cleaning, feature selection, and data transformation can introduce variability and influence the robustness of our analytical pipeline. It is paramount to acknowledge how these preprocessing steps can shape the study’s outcomes. Additionally, our study’s focus on specific taxon groups within the dataset should be considered. The selection of these taxonomic levels and the criteria used for their inclusion or exclusion has bearing on the granularity and relevance of our findings. Finally, the number of data points utilized in our analysis is another crucial factor. A larger dataset provides a broader and potentially more representative sample, which can enhance the reliability and statistical power of our results. Conversely, a smaller dataset may limit the generalizability of our conclusions. A comprehensive understanding of these limiting factors is essential for contextualizing our study’s outcomes and conclusions.

## 5 Conclusion

Over the past two decades, the number of microbiome studies has increased rapidly thanks to the advances in next generation sequencing (NGS) technologies. Lower costs and increasing computational power have enabled us to obtain enormous amounts of data on the diversity and function of a host or habitat’s microbiome. Identifying and accounting for effective taxons in microbiome and disease classification can accelerate disease diagnosis, prognosis, and treatment. Here, we use an efficient machine learning model to identify taxonomic biomarkers that can diagnose diseases. The microBiomeGSM enables researchers to explore the diversity of contributions to disease development by examining metagenomic data at different taxonomic levels. While analyzing microbiome datasets, the microBiomeGSM tool that we present in this study exploits the existing biological knowledge about the taxonomic hierarchy of the species at different levels, such as genus, family, and order. Our results showed that via analyzing different microbiome datasets associated with different diseases, microBiomeGSM builds effective machine learning models to facilitate the diagnosis of diseases. It is anticipated that this study will be a guide for future studies and will guide and improve the studies to be conducted on this topic. With this study, we hope to highlight the importance of taxonomic groups in microbiome-based disease prediction and to facilitate the diagnosis of disease using these taxonomic groups.

## Data availability statement

The original contributions presented in the study are included in the article/Supplementary material, further inquiries can be directed to the corresponding authors.

## Author contributions

BB-G: Methodology, Software, Writing – review & editing, Project administration, Supervision. MT: Methodology, Software, Writing – original draft, Writing – review & editing, Investigation, Visualization. AJ: Data curation, Formal analysis, Methodology, Software, Writing – original draft. DW: Investigation, Methodology, Supervision, Writing – original draft, Project administration, Writing – review & editing. MY: Formal analysis, Funding acquisition, Methodology, Project administration, Software, Supervision, Writing – review & editing.
