# Supplementary material for: microBiomeGSM: the identification of taxonomic biomarkers from metagenomic data using grouping, scoring and modeling (G-S-M) approach
Source: Front Microbiol. 2023 Nov 22;14:1264941. doi: 10.3389/fmicb.2023.1264941 (PMC10703168; doi:10.3389/fmicb.2023.1264941)
Supplement: Supplementary file 1 [file Data_Sheet_1.docx]

Supplementary Material

microBiomeGSM: The identification of taxonomic biomarkers from metagenomic data using Grouping, Scoring and Modeling (G-S-M) approach

Burcu Bakir-Gungor, Mustafa Temiz*, Amhar Jabeer, Di Wu, Malik Yousef*

*** Correspondence:**

Corresponding Authors:

malik.yousef@gmail.com and mustafa.temiz@agu.edu.tr

# Supplementary Figure


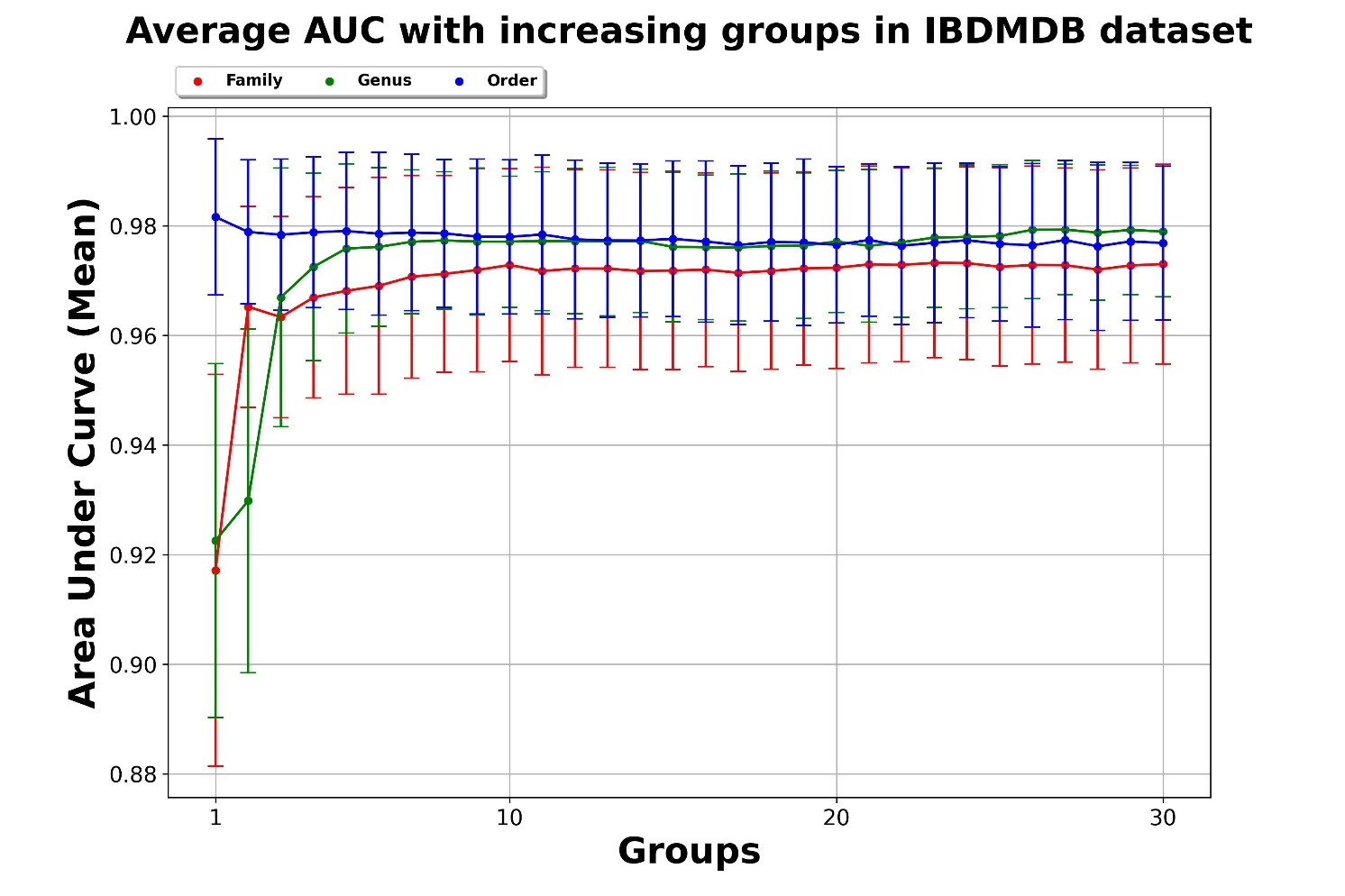


**Supplementary Figure 1. Changes in average AUC scores as the number of groups increases from 1 to 30, tested on the IBDMDB dataset across three different taxonomic levels.**

# Supplementary Tables

**Supplementary Table 1.** Distribution of group sizes for IBDMDB across 3 different taxonomic ranks.

| **Taxonomic Level Used for Grouping** | **0-20** | **20-40** | **40-60** | **60-80** | **80-100** | **100+** | **Sum** |
| --- | --- | --- | --- | --- | --- | --- | --- |
| **Genus** | 182 | 5 | 0 | 0 | 0 | 0 | 187 |
| **Family** | 69 | 7 | 0 | 1 | 0 | 0 | 77 |
| **Order** | 39 | 0 | 1 | 1 | 1 | 1 | 43 |

**Supplementary Table 2.** Performance metrics obtained for all dataset using microBiomeGSM. The effect of grouping at different taxonomic levels (i.e., Order, Family and Genus) is shown. G represents Group.

| **CRC** | | | | | | | | | | | |
| --- | --- | --- | --- | --- | --- | --- | --- | --- | --- | --- | --- |
| **Taxa Rank** | **Metric** | **10 G** | **9 G** | **8 G** | **7 G** | **6 G** | **5 G** | **4 G** | **3 G** | **2 G** | **1 G** |
| **Order** | **Accuracy** | 0,74 | 0,74 | 0,74 | 0,75 | 0,75 | 0,75 | 0,74 | 0,74 | 0,72 | 0,69 |
| **Family** |  | 0,74 | 0,74 | 0,74 | 0,74 | 0,75 | 0,75 | 0,73 | 0,71 | 0,70 | 0,68 |
| **Genus** |  | 0,73 | 0,74 | 0,74 | 0,73 | 0,73 | 0,73 | 0,72 | 0,70 | 0,69 | 0,66 |
| **Order** | **Sensitivity** | 0,73 | 0,73 | 0,72 | 0,73 | 0,72 | 0,72 | 0,72 | 0,70 | 0,69 | 0,63 |
| **Family** |  | 0,69 | 0,69 | 0,69 | 0,69 | 0,69 | 0,64 | 0,56 | 0,50 | 0,46 | 0,42 |
| **Genus** |  | 0,67 | 0,67 | 0,65 | 0,65 | 0,60 | 0,58 | 0,53 | 0,50 | 0,47 | 0,40 |
| **Order** | **Specificity** | 0,76 | 0,76 | 0,77 | 0,76 | 0,77 | 0,77 | 0,77 | 0,77 | 0,76 | 0,76 |
| **Family** |  | 0,79 | 0,79 | 0,80 | 0,80 | 0,81 | 0,87 | 0,90 | 0,91 | 0,93 | 0,94 |
| **Genus** |  | 0,79 | 0,80 | 0,83 | 0,82 | 0,85 | 0,88 | 0,90 | 0,90 | 0,91 | 0,92 |
| **Order** | **AUC** | 0,81 | 0,81 | 0,81 | 0,82 | 0,82 | 0,82 | 0,82 | 0,82 | 0,80 | 0,77 |
| **Family** |  | 0,83 | 0,82 | 0,82 | 0,82 | 0,83 | 0,79 | 0,76 | 0,74 | 0,71 | 0,69 |
| **Genus** |  | 0,78 | 0,79 | 0,79 | 0,79 | 0,78 | 0,77 | 0,76 | 0,75 | 0,73 | 0,68 |
| **IBDMDB** | | | | | | | | | | | |
| **Taxa Rank** | **Metric** | **10 G** | **9 G** | **8 G** | **7 G** | **6 G** | **5 G** | **4 G** | **3 G** | **2 G** | **1 G** |
| **Order** | **Accuracy** | 0,93 | 0,94 | 0,94 | 0,94 | 0,94 | 0,95 | 0,94 | 0,94 | 0,94 | 0,96 |
| **Family** |  | 0,95 | 0,94 | 0,94 | 0,94 | 0,94 | 0,94 | 0,94 | 0,93 | 0,93 | 0,93 |
| **Genus** |  | 0,92 | 0,93 | 0,93 | 0,93 | 0,93 | 0,93 | 0,92 | 0,92 | 0,93 | 0,92 |
| **Order** | **Sensitivity** | 0,97 | 0,98 | 0,97 | 0,98 | 0,98 | 0,98 | 0,98 | 0,98 | 0,98 | 0,98 |
| **Family** |  | 0,98 | 0,98 | 0,98 | 0,98 | 0,99 | 0,98 | 0,98 | 0,97 | 0,97 | 0,99 |
| **Genus** |  | 0,98 | 0,98 | 0,97 | 0,98 | 0,98 | 0,98 | 0,97 | 0,97 | 0,98 | 0,98 |
| **Order** | **Specificity** | 0,86 | 0,86 | 0,87 | 0,86 | 0,87 | 0,88 | 0,87 | 0,87 | 0,87 | 0,93 |
| **Family** |  | 0,87 | 0,86 | 0,86 | 0,86 | 0,86 | 0,86 | 0,86 | 0,84 | 0,83 | 0,81 |
| **Genus** |  | 0,82 | 0,82 | 0,84 | 0,82 | 0,83 | 0,84 | 0,83 | 0,82 | 0,82 | 0,8 |
| **Order** | **AUC** | 0,98 | 0,98 | 0,98 | 0,98 | 0,98 | 0,98 | 0,98 | 0,98 | 0,98 | 0,98 |
| **Family** |  | 0,97 | 0,98 | 0,98 | 0,98 | 0,98 | 0,98 | 0,98 | 0,97 | 0,97 | 0,93 |
| **Genus** |  | 0,97 | 0,97 | 0,97 | 0,97 | 0,97 | 0,97 | 0,97 | 0,96 | 0,92 | 0,91 |
| **T2D** | | | | | | | | | | | |
| **Taxa Rank** | **Metric** | **10 G** | **9 G** | **8 G** | **7 G** | **6 G** | **5 G** | **4 G** | **3 G** | **2 G** | **1 G** |
| **Order** | **Accuracy** | 0,66 | 0,66 | 0,68 | 0,67 | 0,67 | 0,68 | 0,66 | 0,67 | 0,67 | 0,64 |
| **Family** |  | 0,65 | 0,66 | 0,65 | 0,67 | 0,65 | 0,66 | 0,67 | 0,63 | 0,63 | 0,60 |
| **Genus** |  | 0,62 | 0,62 | 0,62 | 0,63 | 0,62 | 0,59 | 0,59 | 0,58 | 0,58 | 0,55 |
| **Order** | **Sensitivity** | 0,65 | 0,64 | 0,68 | 0,64 | 0,62 | 0,66 | 0,64 | 0,64 | 0,67 | 0,66 |
| **Family** |  | 0,65 | 0,66 | 0,66 | 0,65 | 0,64 | 0,64 | 0,69 | 0,66 | 0,65 | 0,66 |
| **Genus** |  | 0,69 | 0,67 | 0,66 | 0,67 | 0,66 | 0,63 | 0,65 | 0,65 | 0,64 | 0,65 |
| **Order** | **Specificity** | 0,67 | 0,69 | 0,67 | 0,70 | 0,71 | 0,69 | 0,67 | 0,69 | 0,67 | 0,61 |
| **Family** |  | 0,64 | 0,65 | 0,64 | 0,69 | 0,66 | 0,69 | 0,65 | 0,60 | 0,61 | 0,54 |
| **Genus** |  | 0,56 | 0,56 | 0,59 | 0,59 | 0,58 | 0,54 | 0,52 | 0,50 | 0,51 | 0,46 |
| **Order** | **AUC** | 0,73 | 0,74 | 0,73 | 0,74 | 0,73 | 0,74 | 0,73 | 0,74 | 0,70 | 0,66 |
| **Family** |  | 0,70 | 0,70 | 0,69 | 0,68 | 0,67 | 0,70 | 0,71 | 0,71 | 0,68 | 0,66 |
| **Genus** |  | 0,68 | 0,67 | 0,67 | 0,69 | 0,67 | 0,65 | 0,62 | 0,59 | 0,58 | 0,59 |
| **IBD** | | | | | | | | | | | |
| **Taxa Rank** | **Metric** | **10 G** | **9 G** | **8 G** | **7 G** | **6 G** | **5 G** | **4 G** | **3 G** | **2 G** | **1 G** |
| **Order** | **Accuracy** | 0,82 | 0,82 | 0,83 | 0,82 | 0,83 | 0,85 | 0,85 | 0,83 | 0,83 | 0,86 |
| **Family** |  | 0,81 | 0,83 | 0,82 | 0,81 | 0,81 | 0,82 | 0,80 | 0,81 | 0,82 | 0,79 |
| **Genus** |  | 0,82 | 0,82 | 0,80 | 0,79 | 0,78 | 0,79 | 0,76 | 0,75 | 0,73 | 0,69 |
| **Order** | **Sensitivity** | 0,84 | 0,83 | 0,83 | 0,83 | 0,83 | 0,85 | 0,85 | 0,84 | 0,85 | 0,87 |
| **Family** |  | 0,83 | 0,84 | 0,83 | 0,84 | 0,84 | 0,83 | 0,81 | 0,83 | 0,82 | 0,77 |
| **Genus** |  | 0,85 | 0,84 | 0,79 | 0,79 | 0,80 | 0,78 | 0,75 | 0,75 | 0,71 | 0,63 |
| **Order** | **Specificity** | 0,81 | 0,82 | 0,83 | 0,81 | 0,84 | 0,85 | 0,85 | 0,81 | 0,81 | 0,84 |
| **Family** |  | 0,79 | 0,81 | 0,81 | 0,79 | 0,78 | 0,80 | 0,79 | 0,79 | 0,82 | 0,81 |
| **Genus** |  | 0,79 | 0,79 | 0,80 | 0,78 | 0,75 | 0,81 | 0,76 | 0,76 | 0,75 | 0,75 |
| **Order** | **AUC** | 0,92 | 0,93 | 0,93 | 0,92 | 0,93 | 0,93 | 0,93 | 0,92 | 0,93 | 0,91 |
| **Family** |  | 0,88 | 0,88 | 0,87 | 0,88 | 0,87 | 0,89 | 0,89 | 0,88 | 0,88 | 0,86 |
| **Genus** |  | 0,89 | 0,88 | 0,88 | 0,86 | 0,85 | 0,85 | 0,83 | 0,82 | 0,80 | 0,74 |

**Supplementary Table 3. Top 5 significant groups that are identified by microBiomeGSM for family taxonomic level for all datasets.**

| **CRC** | |
| --- | --- |
| **Top 5 Family groups** | **List of species** |
| PEPTOSTREPTOCOCCACEAE | Clostridioides_difficile, Criibacterium_bergeronii, Filifactor_alocis, Intestinibacter_bartlettii ……………. |
| PEPTONIPHILACEAE | Anaerococcus_lactolyticus, Anaerococcus_tetradius, Anaerococcus_vaginalis, Finegoldia_magna ………….. |
| FUSOBACTERIACEAE | Cetobacterium_somerae, Fusobacterium_equinum, Fusobacterium_gonidiaformans, Fusobacterium_hwasookii, Fusobacterium_mortiferum ………. |
| BACILLALES_UNCLASSIFIED | Gemella_asaccharolytica, Gemella_bergeri, Gemella_morbillorum, Gemella_sanguinis |
| VEILLONELLACEAE | Allisonella_histaminiformans, Anaeroglobus_geminatus, Dialister_invisus, Dialister_micraerophilus ………………… |
| **IBDMDB** | |
| **Top 5 Family groups** | **List of species** |
| BACTEROIDACEAE | Bacteroides_caccae,Bacteroides_cellulosilyticus, Bacteroides_clarus, Bacteroides_coprocola |
| LACHNOSPIRACEAE | Anaerocolumna_aminovalerica, Anaerosporobacter_mobilis, Anaerostipes_caccae, Anaerostipes_hadrus |
| RUMINOCOCCACEAE | Agathobaculum_butyriciproducens, Anaerofilum_sp_An201, Anaeromassilibacillus_sp_An172, Anaeromassilibacillus_sp_An250 |
| RIKENELLACEAE | Alistipes_finegoldii, Alistipes_indistinctus, Alistipes_inops, Alistipes_onderdonkii |
| FIRMICUTES_UNCLASSIFIED | Firmicutes_bacterium_CAG_110, Firmicutes_bacterium_CAG_145, Firmicutes_bacterium_CAG_170, Firmicutes_bacterium_CAG_238 |
| **IBD** | |
| **Top 5 Family groups** | **List of species** |
| LACHNOSPIRACEAE | Anaerostipes_hadrus, Blautia_hydrogenotrophica, Ruminococcus_gnavus, Ruminococcus_obeum…….. |
| BIFIDOBACTERIACEAE | Bifidobacterium_adolescentis, Bifidobacterium_angulatum, Bifidobacterium_bifidum, Bifidobacterium_catenulatum…… |
| CORIOBACTERIACEAE | Adlercreutzia_equolifaciens, Atopobium_parvulum, Atopobium_rimae, Collinsella_aerofaciens …. |
| RUMINOCOCCACEAE | Anaerotruncus_colihominis, Anaerotruncus_unclassified, Faecalibacterium_prausnitzii, Ruminococcaceae_bacterium_D16….. |
| ERYSIPELOTRICHACEAE | Catenibacterium_mitsuokai, Coprobacillus_sp_29_1, Coprobacillus_sp_D6, Clostridium_innocuum…. |
| **T2D** | |
| **Top 5 Family groups** | **List of species** |
| LACHNOSPIRACEAE | Anaerostipes_hadrus, Blautia_hydrogenotrophica, Ruminococcus_gnavus, Ruminococcus_obeum ……………. |
| BIFIDOBACTERIACEAE | Bifidobacterium_adolescentis, Bifidobacterium_angulatum, Bifidobacterium_bifidum, Bifidobacterium_catenulatum ………….. |
| RUMINOCOCCACEAE | Anaerotruncus_colihominis, Anaerotruncus_unclassified, Faecalibacterium_prausnitzii, Ruminococcaceae_bacterium_D16, Ruminococcus_albus, ………. |
| EUBACTERIACEAE | Eubacterium_brachy, Eubacterium_eligens, Eubacterium_hallii, Eubacterium_ramulus, Eubacterium_rectale …………… |
| CORIOBACTERIACEAE | Adlercreutzia_equolifaciens, Atopobium_parvulu, Atopobium_rimae, Collinsella_aerofaciens ………………… |

**Supplementary Table 4. Top 5 significant groups that are identified by microBiomeGSM for order taxonomic level for all datasets.**

| **CRC** | |
| --- | --- |
| **Top 5 Order groups** | **List of species** |
| CLOSTRIDIALES | Catabacter_hongkongensis, Christensenella_minuta, Butyricicoccus_pullicaecorum, Butyribacterium_methylotrophicum, Clostridium_baratii, ……. |
| TISSIERELLALES | Anaerococcus_lactolyticus,Anaerococcus_tetradius, Anaerococcus_vaginalis, Finegoldia_magna, Parvimonas_micra… |
| BACTEROIDALES | Bacteroides_caccae, Bacteroides_caecimuris, Bacteroides_cellulosilyticus, Bacteroides_clarus, Bacteroides_coprocola ………… |
| FUSOBACTERIALES | Cetobacterium_somerae, Fusobacterium_equinum, Fusobacterium_gonidiaformans, Fusobacterium_hwasookii, Fusobacterium_mortiferum, ………… |
| BACILLALES | Bacillus_aerius, Bacillus_sp_FJAT_27916, Gemella_asaccharolytica, Gemella_bergeri, Gemella_haemolysans, ………… |
| **IBDMDB** | |
| **Top 5 Order groups** | **List of species** |
| BACTEROIDALES | Bacteroides_caccae, Bacteroides_cellulosilyticus, Bacteroides_clarus, Bacteroides_coprocola |
| CLOSTRIDIALES | Catabacter_hongkongensis, Christensenella_minuta, Butyricicoccus_pullicaecorum, Butyribacterium_methylotrophicum |
| FIRMICUTES_UNCLASSIFIED | Firmicutes_bacterium_CAG_110, Firmicutes_bacterium_CAG_145, Firmicutes_bacterium_CAG_170, Firmicutes_bacterium_CAG_238 |
| VEILLONELLALES | Allisonella_histaminiformans, Anaeroglobus_geminatus, Dialister_invisus, Dialister_micraerophilus |
| BURKHOLDERIALES | Oxalobacter_formigenes, Parasutterella_excrementihominis, Sutterella_parvirubra, Turicimonas_muris |
| **IBD** | |
| **Top 5 Order groups** | **List of species** |
| CLOSTRIDIALES | Clostridium_asparagiforme, Clostridium_bolteae, Clostridium_citroniae, Clostridium_clostridioforme, Clostridium_hathewayi, ……. |
| CORIOBACTERIALES | Adlercreutzia_equolifaciens, Atopobium_parvulum, Atopobium_rimae, Collinsella_aerofaciens, Collinsella_unclassified ………… |
| BIFIDOBACTERIALES | Bifidobacterium_adolescentis, Bifidobacterium_angulatum, Bifidobacterium_bifidum, Bifidobacterium_catenulatum, Bifidobacterium_longum………… |
| ERYSIPELOTRICHALES | Catenibacterium_mitsuokai, Coprobacillus_sp_29_1, Coprobacillus_sp_D6, Clostridium_innocuum, Erysipelotrichaceae_bacterium_2_2_44A, ………… |
| BACTEROIDALES | Bacteroides_barnesiae, Bacteroides_caccae, Bacteroides_cellulosilyticus, Bacteroides_clarus … |
| **T2D** | |
| **Top 5 Order groups** | **List of species** |
| CLOSTRIDIALES | Clostridium_asparagiforme, Clostridium_bolteae, Clostridium_citroniae, Clostridium_clostridioforme, Clostridium_hathewayi, ……. |
| BIFIDOBACTERIALES | Bifidobacterium_adolescentis, Bifidobacterium_angulatum, Bifidobacterium_bifidum, Bifidobacterium_catenulatum, |
| CORIOBACTERIALES | Adlercreutzia_equolifaciens, Atopobium_parvulum, Atopobium_rimae, Collinsella_aerofaciens, Collinsella_unclassified ………… |
| BACTEROIDALES | Bacteroides_barnesiae, Bacteroides_caccae, Bacteroides_cellulosilyticus, Bacteroides_clarus, Bacteroides_coprocola, ………… |
| LACTOBACILLALES | Granulicatella_unclassified, Enterococcus_faecium, Lactobacillus_animalis, Lactobacillus_delbrueckii, Lactobacillus_fermentum, ………… |

**Supplementary Table 5. Top 5 significant groups that are identified by microBiomeGSM for genus taxonomic level for all datasets.**

| **CRC** | |
| --- | --- |
| **Top 5 Genus groups** | **List of species** |
| PARVIMONAS | Parvimonas_micra, Parvimonas_sp_KA00067, Parvimonas_sp_oral_taxon_110, Parvimonas_sp_oral_taxon_393 …… |
| PEPTOSTREPTOCOCCUS | Peptostreptococcus_anaerobius, Peptostreptococcus_sp_MV1, Peptostreptococcus_stomatis |
| FUSOBACTERIUM | Fusobacterium_equinum, Fusobacterium_gonidiaformans, Fusobacterium_hwasookii, Fusobacterium_mortiferum, Fusobacterium_naviforme ……………… |
| GEMELLA | Gemella_asaccharolytica, Gemella_bergeri, Gemella_haemolysans, Gemella_morbillorum…… |
| DIALISTER | Dialister_invisus, Dialister_micraerophilus, Dialister_pneumosintes, Dialister_sp_CAG_357, Dialister_succinatiphilus |
| **IBDMDB** | |
| **Top 5 Genus groups** | **List of species** |
| BACTEROIDES | Bacteroides_caccae, Bacteroides_cellulosilyticus, Bacteroides_clarus, Bacteroides_coprocola |
| ALISTIPES | Alistipes_finegoldii, Alistipes_indistinctus, Alistipes_inops, Alistipes_onderdonkii |
| EUBACTERIUM | Eubacterium_coprostanoligenes, Eubacterium_dolichum_CAG_375, Eubacterium_eligens, Eubacterium_hallii |
| ROSEBURIA | Roseburia_faecis, Roseburia_hominis, Roseburia_intestinalis, Roseburia_inulinivorans |
| FIRMICUTES_UNCLASSIFIED | Firmicutes_bacterium_CAG_110, Firmicutes_bacterium_CAG_145, Firmicutes_bacterium_CAG_170, Firmicutes_bacterium_CAG_238 |
| **IBD** | |
| **Top 5 Genus groups** | **List of species** |
| BLAUTIA | Blautia_hydrogenotrophica, Ruminococcus_gnavus, Ruminococcus_obeum, Ruminococcus_torques, Blautia_hansenii …… |
| BIFIDOBACTERIUM | Bifidobacterium_adolescentis, Bifidobacterium_angulatum, Bifidobacterium_bifidum, Bifidobacterium_catenulatum ……… |
| EUBACTERIUM | Eubacterium_brachy, Eubacterium_eligens, Eubacterium_hallii, Eubacterium_ramulus, Eubacterium_rectale …… |
| DOREA | Dorea_formicigenerans, Dorea_longicatena, Dorea_unclassified… |
| COLLINSELLA | Collinsella_aerofaciens, Collinsella_unclassified, Collinsella_intestinalis, Collinsella_stercoris, Collinsella_tanakaei ……………… |
| **T2D** | |
| **Top 5 Genus groups** | **List of species** |
| EUBACTERIUM | Eubacterium_brachy, Eubacterium_eligens, Eubacterium_hallii, Eubacterium_ramulus, Eubacterium_rectale …… |
| BIFIDOBACTERIUM | Bifidobacterium_adolescentis, Bifidobacterium_angulatum, Bifidobacterium_bifidum, Bifidobacterium_catenulatum ……… |
| BLAUTIA | Blautia_hydrogenotrophica, Ruminococcus_gnavus, Ruminococcus_obeum, Ruminococcus_torques, Blautia_hansenii ……………… |
| DOREA | Dorea_formicigenerans, Dorea_longicatena, Dorea_unclassified |
| LACHNOSPIRACEAE_NONAME | lachnospiraceae_bacterium_1_1_57FAA, Lachnospiraceae_bacterium_1_4_56FAA, Lachnospiraceae_bacterium_2_1_58FAA, Lachnospiraceae_bacterium_3_1_46FAA, ……………… |

**Supplementary Table 6.** Species names identified for the top 10 family taxon groups for T2D.

| **Name of Taxa for T2D (Family)** | **Species Name** |
| --- | --- |
| LACHNOSPIRACEAE | Anaerostipes hadrus, Blautia hydrogenotrophica, Ruminococcus gnavus, Ruminococcus obeum, Ruminococcus torques, Butyrivibrio crossotus, Butyrivibrio unclassified', Coprococcus catus, Coprococcus comes, Coprococcus eutactus, Coprococcus sp ART55 1, Dorea formicigenerans, Dorea longicatena, Dorea unclassified', Lachnospiraceae bacterium 1 1 57FAA, Lachnospiraceae bacterium 1 4 56FAA, Lachnospiraceae bacterium 2 1 58FAA, Lachnospiraceae bacterium 3 1 46FAA, Lachnospiraceae bacterium 3 1 57FAA CT1, Lachnospiraceae bacterium 4 1 37FAA, Lachnospiraceae bacterium 5 1 63FAA, Lachnospiraceae bacterium 7 1 58FAA, Lachnospiraceae bacterium 8 1 57FAA, Lachnospiraceae bacterium 9 1 43BFAA, Roseburia hominis, Roseburia intestinalis, Roseburia inulinivorans, Roseburia unclassified', Anaerostipes caccae, Anaerostipes unclassified', Blautia hansenii, Blautia producta, Ruminococcus obeum, Ruminococcus obeum, Ruminococcus torques, Dorea formicigenerans, Lachnoanaerobaculum saburreum, Lachnospiraceae bacterium 5 1 57FAA, Lachnospiraceae bacterium 6 1 63FAA, Lachnospiraceae bacterium ICM7, Lachnospiraceae bacterium oral taxon 082, Marvinbryantia formatexigens, Oribacterium sinus, Roseburia intestinalis, Stomatobaculum longum, Anaerostipes sp 3 2 56FAA, Cellulosilyticum lentocellum, Lachnospiraceae oral taxon 107, Shuttleworthia satelles, Catonella morbi, Johnsonella ignava, Lachnoanaerobaculum saburreum, Lachnospiraceae bacterium 2 1 46FAA, Oribacterium sp ACB1, Oribacterium sp ACB7, Oribacterium sp ACB8, Oribacterium sp oral taxon 078, Oribacterium sp oral taxon 108, Roseburia intestinalis, Oribacterium sp oral taxon 078, Blautia sp KLE 1732, Dorea formicigenerans |
| BIFIDOBACTERIACEAE | Bifidobacterium adolescentis, Bifidobacterium angulatum, Bifidobacterium bifidum, Bifidobacterium catenulatum, Bifidobacterium longum, Bifidobacterium pseudocatenulatum, Gardnerella vaginalis, Alloscardovia omnicolens, Bifidobacterium animalis, Bifidobacterium breve, Bifidobacterium dentium, Bifidobacterium longum, Parascardovia denticolens, Scardovia unclassified', Scardovia wiggsiae, Bifidobacterium minimum, Bifidobacterium pseudolongum, Scardovia inopinata |
| RUMINOCOCCACEAE | Anaerotruncus colihominis Anaerotruncus unclassified', Faecalibacterium prausnitzii, Faecalibacterium prausnitzii, Faecalibacterium prausnitzii, Ruminococcaceae bacterium D16, Ruminococcus albus, Ruminococcus bromii, Ruminococcus callidus, Ruminococcus champanellensis, Ruminococcus flavefaciens, Ruminococcus lactaris, Ruminococcus sp 5 1 39BFAA, Subdoligranulum unclassified', Subdoligranulum variabile, Faecalibacterium prausnitzii, Ruminococcus lactaris, Ruminococcus lactaris, Ruminococcus sp JC304, Ruminococcus sp, Subdoligranulum sp 4 3 54A2FAA, Faecalibacterium prausnitzii, Faecalibacterium prausnitzii |
| EUBACTERIACEAE | Eubacterium brachy, Eubacterium eligens, Eubacterium hallii, Eubacterium ramulus, Eubacterium rectale, Eubacterium siraeum, Eubacterium ventriosum, Anaerofustis stercorihominis, Eubacterium limosum, Eubacterium rectale, Eubacterium saphenum, Eubacterium siraeum, Eubacterium sp 3 1 31, Eubacterium siraeum, Eubacteriaceae bacterium ACC19a, Eubacteriaceae bacterium CM2, Eubacteriaceae bacterium CM5, Eubacteriaceae bacterium OBRC8, Eubacteriaceae noname unclassified' Eubacterium rectale, Pseudoramibacter alactolyticus, Eubacterium rectale |
| CORIOBACTERIACEAE | Adlercreutzia equolifaciens, Atopobium parvulum, Atopobium rimae, Collinsella aerofaciens, Collinsella unclassified', Coriobacteriaceae bacterium phI, Eggerthella lenta, Eggerthella unclassified', Gordonibacter pamelaeae, Olsenella unclassified', Slackia piriformis, Atopobium vaginae, Collinsella intestinalis, Collinsella stercoris, Collinsella tanakaei, Eggerthella sp 1 3 56FAA, Enterorhabdus caecimuris, Atopobium minutum, Cryptobacterium curtum, Eggerthella sp HGA1, Olsenella uli, Slackia exigua, Slackia unclassified', Atopobium sp ICM58, Atopobium sp oral taxon 199, Atopobium vaginae, Coriobacteriaceae bacterium BV3Ac1, Collinsella sp GD3, Olsenella profusa, Olsenella sp oral taxon 809 |
| CLOSTRIDIALES_FAMILY_XIII_INCERTAE_SEDIS | Eubacterium infirmum, Mogibacterium sp CM50 |
| ERYSIPELOTRICHACEAE | Catenibacterium mitsuokai, Coprobacillus sp 29 1, Coprobacillus sp D6, Clostridium innocuum, Erysipelotrichaceae bacterium 2 2 44A, Erysipelotrichaceae bacterium 6 1 45, Eubacterium biforme, Eubacterium cylindroides, Holdemania filiformis, Solobacterium moorei, Turicibacter sanguinis, Turicibacter unclassified', Allobaculum stercoricanis, Bulleidia extructa, Coprobacillus unclassified', Eggerthia catenaformis, Clostridium ramosum, Clostridium spiroforme, Erysipelotrichaceae bacterium 21 3, Erysipelotrichaceae bacterium 3 1 53, Erysipelotrichaceae bacterium 5 2 54FAA, Eubacterium dolichum ,Holdemania sp AP2, Holdemania unclassified' |
| PEPTOSTREPTOCOCCACEAE | Clostridium bartlettii, Peptostreptococcaceae noname unclassified', Peptostreptococcus unclassified', Clostridium glycolicum, Peptostreptococcus stomatis, Clostridium difficile, Clostridium difficile, Clostridium sordellii, Peptostreptococcus anaerobius, Filifactor alocis, Clostridium hiranonis, Eubacterium yurii, Clostridium bifermentans |
| CARNOBACTERIACEAE | Granulicatella unclassified', Granulicatella adiacens, Dolosigranulum pigrum, Granulicatella elegans, Alloiococcus otitis, Carnobacterium maltaromaticum |
| BACTEROIDACEAE | Bacteroides barnesiae, Bacteroides caccae, Bacteroides cellulosilyticus, Bacteroides clarus, Bacteroides coprocola, Bacteroides coprophilus, Bacteroides dorei, Bacteroides eggerthii, Bacteroides faecis, Bacteroides finegoldii, Bacteroides fragilis, Bacteroides intestinalis, Bacteroides massiliensis, Bacteroides nordii, Bacteroides ovatus, Bacteroides plebeius, Bacteroides salyersiae, Bacteroides sp 4 3 47FAA, Bacteroides stercoris, Bacteroides thetaiotaomicron, Bacteroides uniformis, Bacteroides uniformis, Bacteroides vulgatus, Bacteroides xylanisolvens, Bacteroides cellulosilyticus, Bacteroides eggerthii, Bacteroides finegoldii, Bacteroides fluxus, Bacteroides fragilis, Bacteroides fragilis, Bacteroides gallinarum, Bacteroides oleiciplenus, Bacteroides ovatus, Bacteroides ovatus, Bacteroides salanitronis, Bacteroides salyersiae, Bacteroides sp 1 1 14, Bacteroides sp 1 1 30, Bacteroides sp 1 1 6, Bacteroides sp 2 1 22, Bacteroides sp 2 1 56FAA, Bacteroides sp 3 1 19, Bacteroides sp 3 1 23, Bacteroides sp 3 2 5, Bacteroides sp 9 1 42FAA, Bacteroides thetaiotaomicron, Bacteroides xylanisolvens, Bacteroides caccae, Bacteroides fragilis, Bacteroides ovatus, Bacteroides ovatus, Bacteroides sp 3 1 40A, Bacteroides dorei, Bacteroides vulgatus, Bacteroides cellulosilyticus, Bacteroides pyogenes, Bacteroides sp D22, Bacteroides stercoris, Bacteroides vulgatus, Bacteroides xylanisolvens, Bacteroides sp 2 2 4 |

**Supplementary Table 7.** Species names identified for the top 10 order taxon groups for T2D.

| **Name of Taxa for T2D (Order)** | **Species Name** |
| --- | --- |
| CLOSTRIDIALES | Anaerococcus hydrogenalis, Anaerococcus lactolyticus, Anaerococcus obesiensis, Anaerococcus prevotii, Anaerococcus prevotii, Anaerococcus prevotii, Anaerococcus sp PH9, Anaerococcus tetradius, Anaerococcus vaginalis, Anaerofustis stercorihominis, Anaerostipes caccae, Anaerostipes hadrus, Anaerostipes sp 3 2 56FAA, Anaerostipes unclassified, Anaerotruncus colihominis, Anaerotruncus unclassified', Bacteroides pectinophilus, Blautia hansenii, Blautia hydrogenotrophica, Blautia producta, Blautia sp KLE 1732, Butyricicoccus pullicaecorum, Butyrivibrio crossotus, Butyrivibrio unclassified, Catonella morbi, Cellulosilyticum lentocellum, Clostridiaceae bacterium JC118, Clostridiales bacterium 1 7 47FAA, Clostridiales bacterium BV3C26, Clostridiales genomosp BVAB3, Clostridium asparagiforme, Clostridium bartlettii, Clostridium beijerinckii, Clostridium bifermentans, Clostridium bolteae, Clostridium bolteae, Clostridium bolteae, Clostridium butyricum, Clostridium celatum, Clostridium citroniae, Clostridium clostridioforme, Clostridium clostridioforme, Clostridium colicanis, Clostridium difficile, Clostridium difficile, Clostridium glycolicum, Clostridium hathewayi, Clostridium hathewayi, Clostridium hathewayi, Clostridium hathewayi, Clostridium hiranonis, Clostridium hylemonae, Clostridium leptum, Clostridium methylpentosum, Clostridium nexile, Clostridium perfringens, Clostridium phytofermentans, Clostridium saccharolyticum, Clostridium scindens, Clostridium sordellii, Clostridium sp 7 2 43FAA, Clostridium sp ATCC BAA 442, Clostridium sp D5, Clostridium sp HGF2, Clostridium sp KLE 1755, Clostridium sp L2 50, Clostridium sp M62 1, Clostridium sp MSTE9, Clostridium sp SS2 1, Clostridium sporogenes, Clostridium symbiosum, Coprococcus catus, Coprococcus comes, Coprococcus eutactus, Coprococcus sp ART55 1, Desulfotomaculum ruminis, Dorea formicigenerans, Dorea formicigenerans, Dorea formicigenerans, Dorea longicatena, Dorea unclassified', Eubacteriaceae bacterium ACC19a, Eubacteriaceae bacterium CM2, Eubacteriaceae bacterium CM5, Eubacteriaceae bacterium OBRC8, Eubacteriaceae noname unclassified', Eubacterium brachy, Eubacterium eligens, Eubacterium hallii, Eubacterium infirmum, Eubacterium limosum, Eubacterium ramulus, Eubacterium rectale, Eubacterium rectale, Eubacterium rectale, Eubacterium rectale, Eubacterium saphenum, Eubacterium siraeum, Eubacterium siraeum, Eubacterium siraeum, Eubacterium sp 3 1 31, Eubacterium ventriosum, Eubacterium yurii, Faecalibacterium prausnitzii, Faecalibacterium prausnitzii, Faecalibacterium prausnitzii, Faecalibacterium prausnitzii, Faecalibacterium prausnitzii, Faecalibacterium prausnitzii, Filifactor alocis, Finegoldia magna, Finegoldia magna, Flavonifractor plautii, Helcococcus kunzii, Johnsonella ignava, Lachnoanaerobaculum saburreum, Lachnoanaerobaculum saburreum, Lachnospiraceae bacterium 1 1 57FAA, Lachnospiraceae bacterium 1 4 56FAA, Lachnospiraceae bacterium 2 1 46FAA, Lachnospiraceae bacterium 2 1 58FAA, Lachnospiraceae bacterium 3 1 46FAA, Lachnospiraceae bacterium 3 1 57FAA CT1, Lachnospiraceae bacterium 4 1 37FAA, Lachnospiraceae bacterium 5 1 57FAA, Lachnospiraceae bacterium 5 1 63FAA, Lachnospiraceae bacterium 6 1 63FAA, Lachnospiraceae bacterium 7 1 58FAA, Lachnospiraceae bacterium 8 1 57FAA, Lachnospiraceae bacterium 9 1 43BFAA, Lachnospiraceae bacterium ICM7, Lachnospiraceae bacterium oral taxon 082, Lachnospiraceae oral taxon 107, Marvinbryantia formatexigens, Mogibacterium sp CM50, Oribacterium sinus, Oribacterium sp ACB1, Oribacterium sp ACB7, Oribacterium sp ACB8, Oribacterium sp oral taxon 078, Oribacterium sp oral taxon 078, Oribacterium sp oral taxon 108, Oscillibacter sp KLE 1728, Oscillibacter sp KLE 1745, Oscillibacter unclassified, Oscillibacter valericigenes, Parvimonas micra, Parvimonas sp oral taxon 110, Parvimonas unclassified', Peptoniphilus duerdenii, Peptoniphilus harei, Peptoniphilus lacrimalis, Peptoniphilus lacrimalis, Peptoniphilus rhinitidis, Peptoniphilus sp BV3AC2, Peptoniphilus sp JC140, Peptoniphilus sp oral taxon 375, Peptoniphilus sp oral taxon 836, Peptoniphilus timonensis, Peptostreptococcaceae noname unclassified, Peptostreptococcus anaerobius, Peptostreptococcus stomatis, Peptostreptococcus unclassified, Pseudoflavonifractor capillosus, Pseudoramibacter alactolyticus, Roseburia hominis, Roseburia intestinalis, Roseburia intestinalis, Roseburia intestinalis, Roseburia inulinivorans, Roseburia unclassified, Ruminococcaceae bacterium D16, Ruminococcus albus, Ruminococcus bromii, Ruminococcus callidus, Ruminococcus champanellensis, Ruminococcus flavefaciens, Ruminococcus gnavus, Ruminococcus lactaris, Ruminococcus lactaris, Ruminococcus lactaris, Ruminococcus obeum, Ruminococcus obeum, Ruminococcus obeum, Ruminococcus sp, Ruminococcus sp 5 1 39BFAA, Ruminococcus sp JC304, Ruminococcus torques, Ruminococcus torques, Shuttleworthia satelles, Stomatobaculum longum, Subdoligranulum sp 4 3 54A2FAA, Subdoligranulum unclassified, Subdoligranulum variabile |
| BIFIDOBACTERIALES | Alloscardovia omnicolens, Bifidobacterium adolescentis, Bifidobacterium angulatum, Bifidobacterium animalis, Bifidobacterium bifidum, Bifidobacterium breve, Bifidobacterium catenulatum, Bifidobacterium dentium, Bifidobacterium longum, Bifidobacterium longum, Bifidobacterium minimum, Bifidobacterium pseudocatenulatum, Bifidobacterium pseudolongum, Gardnerella vaginalis, Parascardovia denticolens, Scardovia inopinata, Scardovia unclassified, Scardovia wiggsiae |
| CORIOBACTERIALES | Slackia unclassified, Slackia piriformis, Slackia exigua, Olsenella unclassified, Olsenella uli, Olsenella sp oral taxon 809, Olsenella profusa, Gordonibacter pamelaeae, Enterorhabdus caecimuris, Eggerthella unclassified, Eggerthella sp HGA1, Eggerthella sp 1 3 56FAA, Eggerthella lenta, Cryptobacterium curtum, Coriobacteriaceae bacterium phI, Coriobacteriaceae bacterium BV3Ac1, Collinsella unclassified, Collinsella tanakaei, Collinsella stercoris, Collinsella sp GD3, Collinsella intestinalis, Collinsella aerofaciens, Atopobium vaginae, Atopobium vaginae, Atopobium sp oral taxon 199, Atopobium sp ICM58, Atopobium rimae, Atopobium parvulum, Atopobium minutum, Adlercreutzia equolifaciens |
| BACTEROIDALES | Alistipes finegoldii, Alistipes indistinctus, Alistipes onderdonkii, Alistipes putredinis, Alistipes senegalensis, Alistipes shahii, Alistipes sp AP11, Alistipes sp HGB5, Alistipes sp JC136, Alistipes unclassified, Alloprevotella rava, Alloprevotella tannerae, Alloprevotella unclassified, Bacteroidales bacterium ph8, Bacteroides barnesiae, Bacteroides caccae, Bacteroides caccae, Bacteroides cellulosilyticus, Bacteroides cellulosilyticus, Bacteroides cellulosilyticus, Bacteroides clarus, Bacteroides coprocola, Bacteroides coprophilus, Bacteroides dorei, Bacteroides dorei, Bacteroides eggerthii, Bacteroides eggerthii, Bacteroides faecis, Bacteroides finegoldii, Bacteroides finegoldii, Bacteroides fluxus, Bacteroides fragilis, Bacteroides fragilis, Bacteroides fragilis, Bacteroides fragilis, Bacteroides gallinarum, Bacteroides intestinalis, Bacteroides massiliensis, Bacteroides nordii, Bacteroides oleiciplenus, Bacteroides ovatus, Bacteroides ovatus, Bacteroides ovatus, Bacteroides ovatus, Bacteroides ovatus, Bacteroides plebeius, Bacteroides pyogenes, Bacteroides salanitronis, Bacteroides salyersiae, Bacteroides salyersiae, Bacteroides sp 1 1 14, Bacteroides sp 1 1 30, Bacteroides sp 1 1 6, Bacteroides sp 2 1 22, Bacteroides sp 2 1 56FAA, Bacteroides sp 2 2 4, Bacteroides sp 3 1 19, Bacteroides sp 3 1 23, Bacteroides sp 3 1 40A, Bacteroides sp 3 2 5, Bacteroides sp 4 3 47FAA, Bacteroides sp 9 1 42FAA, Bacteroides sp D22, Bacteroides stercoris, Bacteroides stercoris, Bacteroides thetaiotaomicron, Bacteroides thetaiotaomicron, Bacteroides uniformis, Bacteroides uniformis, Bacteroides vulgatus, Bacteroides vulgatus, Bacteroides vulgatus, Bacteroides xylanisolvens, Bacteroides xylanisolvens, Bacteroides xylanisolvens, Barnesiella intestinihominis, Butyricimonas synergistica, Candidatus Prevotella conceptionensis, Coprobacter fastidiosus, Dysgonomonas gadei, Dysgonomonas mossii, Dysgonomonas unclassified, Odoribacter laneus, Odoribacter splanchnicus, Odoribacter unclassified', Parabacteroides distasonis, Parabacteroides goldsteinii, Parabacteroides johnsonii, Parabacteroides johnsonii, Parabacteroides merdae, Parabacteroides merdae, Parabacteroides merdae, Parabacteroides sp 20 3, Parabacteroides sp D13, Parabacteroides unclassified, Paraprevotella clara, Paraprevotella unclassified, Paraprevotella xylaniphila, Porphyromonas asaccharolytica, Porphyromonas bennonis, Porphyromonas catoniae, Porphyromonas crevioricanis, Porphyromonas endodontalis, Porphyromonas gingivalis, Porphyromonas gulae, Porphyromonas macacae, Porphyromonas somerae, Porphyromonas sp oral taxon 278, Porphyromonas sp oral taxon 279, Porphyromonas uenonis, Prevotella amnii, Prevotella amnii, Prevotella baroniae, Prevotella bergensis, Prevotella bivia, Prevotella buccae, Prevotella buccalis, Prevotella copri, Prevotella dentalis, Prevotella denticola, Prevotella disiens, Prevotella disiens, Prevotella histicola, Prevotella intermedia, Prevotella loescheii, Prevotella maculosa, Prevotella maculosa, Prevotella maculosa, Prevotella marshii, Prevotella melaninogenica, Prevotella melaninogenica, Prevotella melaninogenica, Prevotella micans, Prevotella multiformis, Prevotella multisaccharivorax, Prevotella nanceiensis, Prevotella nigrescens, Prevotella oralis, Prevotella oris, Prevotella oulorum, Prevotella pallens, Prevotella paludivivens, Prevotella pleuritidis, Prevotella saccharolytica, Prevotella salivae, Prevotella sp BV3P1, Prevotella sp C561, Prevotella sp oral taxon 299, Prevotella sp oral taxon 306, Prevotella sp oral taxon 317, Prevotella sp oral taxon 473, Prevotella stercorea, Prevotella timonensis, Prevotella veroralis, Prevotella veroralis, Tannerella forsythia |
| LACTOBACILLALES | Abiotrophia defectiva, Aerococcus urinae, Aerococcus viridans, Aerococcus viridans, Alloiococcus otitis, Bavariicoccus seileri, Carnobacterium maltaromaticum, Catellicoccus marimammalium, Dolosigranulum pigrum, Enterococcus asini, Enterococcus avium, Enterococcus casseliflavus, Enterococcus cecorum, Enterococcus dispar, Enterococcus durans, Enterococcus faecalis, Enterococcus faecium, Enterococcus faecium, Enterococcus gallinarum, Enterococcus gilvus, Enterococcus hirae, Enterococcus italicus, Enterococcus malodoratus, Enterococcus mundtii, Enterococcus pallens, Enterococcus raffinosus, Enterococcus saccharolyticus, Enterococcus sp 7L76, Eremococcus coleocola, Facklamia hominis, Facklamia ignava, Facklamia languida, Facklamia unclassified, Granulicatella adiacens, Granulicatella elegans, Granulicatella unclassified, Lactobacillus acidophilus, Lactobacillus amylovorus, Lactobacillus animalis, Lactobacillus antri, Lactobacillus brevis, Lactobacillus buchneri, Lactobacillus casei paracasei, Lactobacillus coleohominis, Lactobacillus coryniformis, Lactobacillus crispatus, Lactobacillus crispatus, Lactobacillus curvatus, Lactobacillus delbrueckii, Lactobacillus equicursoris, Lactobacillus farciminis, Lactobacillus fermentum, Lactobacillus fermentum, Lactobacillus fructivorans, Lactobacillus gasseri, Lactobacillus gastricus, Lactobacillus helveticus, Lactobacillus iners, Lactobacillus iners, Lactobacillus jensenii, Lactobacillus jensenii, Lactobacillus johnsonii, Lactobacillus mucosae, Lactobacillus oris, Lactobacillus oris, Lactobacillus otakiensis, Lactobacillus pentosus, Lactobacillus plantarum, Lactobacillus reuteri, Lactobacillus rhamnosus, Lactobacillus ruminis, Lactobacillus saerimneri, Lactobacillus sakei, Lactobacillus salivarius, Lactobacillus sanfranciscensis, Lactobacillus sp 7 1 47FAA, Lactobacillus ultunensis, Lactobacillus vaginalis, Lactobacillus versmoldensis, Lactococcus garvieae, Lactococcus lactis, Lactococcus raffinolactis, Leuconostoc carnosum, Leuconostoc citreum, Leuconostoc fallax, Leuconostoc gasicomitatum, Leuconostoc gelidum, Leuconostoc kimchii, Leuconostoc lactis, Leuconostoc mesenteroides, Leuconostoc pseudomesenteroides, Leuconostoc unclassified', Oenococcus oeni, Pediococcus acidilactici, Pediococcus lolii, Pediococcus pentosaceus, Pediococcus unclassified, Streptococcus agalactiae, Streptococcus anginosus, Streptococcus australis, Streptococcus canis, Streptococcus constellatus, Streptococcus cristatus, Streptococcus downei, Streptococcus dysgalactiae, Streptococcus equi, Streptococcus gallolyticus, Streptococcus gordonii, Streptococcus infantarius, Streptococcus infantis, Streptococcus infantis, Streptococcus intermedius, Streptococcus lutetiensis, Streptococcus macedonicus, Streptococcus massiliensis, Streptococcus minor, Streptococcus mitis oralis pneumoniae, Streptococcus mutans, Streptococcus oligofermentans, Streptococcus parasanguinis, Streptococcus parasanguinis, Streptococcus parasanguinis, Streptococcus parauberis, Streptococcus pasteurianus, Streptococcus peroris, Streptococcus pseudopneumoniae, Streptococcus pyogenes, Streptococcus salivarius, Streptococcus sanguinis, Streptococcus sobrinus, Streptococcus sp 2 1 36FAA, Streptococcus sp AS14, Streptococcus sp BS35b, Streptococcus sp C150, Streptococcus sp C300, Streptococcus sp F0441, Streptococcus sp F0442, Streptococcus sp GMD4S, Streptococcus sp GMD5S, Streptococcus sp HPH0090, Streptococcus sp I G2, Streptococcus sp I P16, Streptococcus sp M143, Streptococcus sp M334, Streptococcus sp oral taxon 056, Streptococcus sp SK140, Streptococcus sp SK643, Streptococcus thermophilus, Streptococcus tigurinus, Streptococcus vestibularis, Tetragenococcus halophilus, Vagococcus lutrae, Weissella cibaria, Weissella confusa, Weissella paramesenteroides, Weissella unclassified |
| ERYSIPELOTRICHALES | Allobaculum stercoricanis, Bulleidia extructa, Catenibacterium mitsuokai, Clostridium innocuum, Clostridium ramosum, Clostridium spiroforme, Coprobacillus sp 29 1, Coprobacillus sp D6, Coprobacillus unclassified, Eggerthia catenaformis, Erysipelotrichaceae bacterium 2 2 44A, Erysipelotrichaceae bacterium 21 3, Erysipelotrichaceae bacterium 3 1 53, Erysipelotrichaceae bacterium 5 2 54FAA, Erysipelotrichaceae bacterium 6 1 45, Eubacterium biforme, Eubacterium cylindroides, Eubacterium dolichum, Holdemania filiformis, Holdemania sp AP2, Holdemania unclassified, Solobacterium moorei, Turicibacter sanguinis, Turicibacter unclassified |
| SELENOMONADALES | Acidaminococcus fermentans, Acidaminococcus intestini, Acidaminococcus sp BV3L6, Acidaminococcus sp D21, Acidaminococcus sp HPA0509, Acidaminococcus unclassified, Anaeroglobus geminatus, Centipeda periodontii, Dialister invisus, Dialister micraerophilus, Dialister micraerophilus, Dialister succinatiphilus, Megamonas funiformis, Megamonas hypermegale, Megamonas rupellensis, Megamonas unclassified, Megasphaera elsdenii, Megasphaera genomosp type 1, Megasphaera micronuciformis, Megasphaera sp BV3C16 1, Megasphaera sp UPII 135 E, Megasphaera sp UPII 199 6, Megasphaera unclassified, Mitsuokella multacida, Mitsuokella unclassified, Phascolarctobacterium succinatutens, Selenomonas artemidis, Selenomonas bovis, Selenomonas flueggei, Selenomonas infelix, Selenomonas noxia, Selenomonas noxia, Selenomonas sp CM52, Selenomonas sp F0473, Selenomonas sp FOBRC6, Selenomonas sp FOBRC9, Selenomonas sp oral taxon 137, Selenomonas sp oral taxon 138, Selenomonas sp oral taxon 149, Selenomonas sp oral taxon 892, Selenomonas sputigena, Veillonella atypica, Veillonella atypica, Veillonella dispar, Veillonella parvula, Veillonella parvula, Veillonella parvula, Veillonella ratti, Veillonella sp 3 1 44, Veillonella sp 6 1 27, Veillonella sp ACP1, Veillonella sp HPA0037, Veillonella sp oral taxon 158, Veillonella sp oral taxon 780, Veillonella unclassified |
| VERRUCOMICROBIALES | Akkermansia muciniphila |
| METHANOBACTERIALES | Methanocaldococcus unclassified |
| BACILLALES | Staphylococcus xylosus, Staphylococcus warneri, Staphylococcus vitulinus, Staphylococcus sp HGB0015, Staphylococcus sp E463, Staphylococcus sp AL1, Staphylococcus simulans, Staphylococcus saprophyticus, Staphylococcus pseudintermedius, Staphylococcus pettenkoferi, Staphylococcus pasteuri, Staphylococcus massiliensis, Staphylococcus lugdunensis, Staphylococcus lentus, Staphylococcus hominis, Staphylococcus haemolyticus, Staphylococcus equorum, Staphylococcus epidermidis, Staphylococcus carnosus, Staphylococcus caprae capitis, Staphylococcus caprae capitis, Staphylococcus aureus, Staphylococcus arlettae, Salinicoccus unclassified, Paenibacillus lactis, Paenibacillus barengoltzii, Marinococcus halotolerans, Macrococcus caseolyticus, Lysinibacillus unclassified', Lysinibacillus sphaericus, Lysinibacillus fusiformis, Lysinibacillus boronitolerans, Listeria monocytogenes, Gracilibacillus unclassified, Geobacillus unclassified, Gemella unclassified, Gemella sanguinis, Gemella morbillorum, Gemella haemolysans, Gemella haemolysans, Gemella haemolysans, Gemella bergeri, Exiguobacterium unclassified, Exiguobacterium sibiricum, Exiguobacterium pavilionensis, Brevibacillus brevis, Brevibacillus borstelensis, Bacillus subtilis, Bacillus smithii, Bacillus pumilus, Bacillus megaterium, Bacillus licheniformis, Bacillus cereus thuringiensis, Bacillus amyloliquefaciens, Anoxybacillus unclassified', Anoxybacillus flavithermus, Alicyclobacillus unclassified, Alicyclobacillus pohliae |

**Supplementary Table 8.** Species names identified for the top 10 genus taxon groups for T2D.

| **Name of Taxa for T2D (Genus)** | **Species Name** |
| --- | --- |
| EUBACTERIUM | Eubacterium ventriosum, Eubacterium sp 3 1 31, Eubacterium siraeum, Eubacterium siraeum, Eubacterium siraeum, Eubacterium saphenum, Eubacterium rectale, Eubacterium rectale, Eubacterium rectale, Eubacterium rectale, Eubacterium ramulus, Eubacterium limosum, Eubacterium hallii, Eubacterium eligens, Eubacterium brachy |
| BIFIDOBACTERIUM | Bifidobacterium pseudolongum, Bifidobacterium pseudocatenulatum, Bifidobacterium minimum, Bifidobacterium longum, Bifidobacterium longum, Bifidobacterium dentium, Bifidobacterium catenulatum, Bifidobacterium breve, Bifidobacterium bifidum, Bifidobacterium animalis, Bifidobacterium angulatum, Bifidobacterium adolescentis |
| BLAUTIA | Ruminococcus torques, Ruminococcus torques, Ruminococcus obeum, Ruminococcus obeum, Ruminococcus obeum, Ruminococcus gnavus, Blautia sp KLE 1732, Blautia producta, Blautia hydrogenotrophica, Blautia hansenii |
| DOREA | Dorea unclassified, Dorea longicatena, Dorea formicigenerans, Dorea formicigenerans, Dorea formicigenerans |
| LACHNOSPIRACEAE NONAME | Lachnospiraceae oral taxon 107, Lachnospiraceae bacterium oral taxon 082, Lachnospiraceae bacterium ICM7, Lachnospiraceae bacterium 9 1 43BFAA, Lachnospiraceae bacterium 8 1 57FAA, Lachnospiraceae bacterium 7 1 58FAA, Lachnospiraceae bacterium 6 1 63FAA, Lachnospiraceae bacterium 5 1 63FAA, Lachnospiraceae bacterium 5 1 57FAA, Lachnospiraceae bacterium 4 1 37FAA, Lachnospiraceae bacterium 3 1 57FAA CT1, Lachnospiraceae bacterium 3 1 46FAA, Lachnospiraceae bacterium 2 1 58FAA, Lachnospiraceae bacterium 2 1 46FAA, Lachnospiraceae bacterium 1 4 56FAA, Lachnospiraceae bacterium 1 1 57FAA |
| RUMINOCOCCUS | Ruminococcus sp JC304, Ruminococcus sp 5 1 39BFAA, Ruminococcus sp, Ruminococcus lactaris, Ruminococcus lactaris, Ruminococcus lactaris, Ruminococcus flavefaciens, Ruminococcus champanellensis, Ruminococcus callidus, Ruminococcus bromii, Ruminococcus albus |
| COPROCOCCUS | Coprococcus sp ART55 1, Coprococcus eutactus, Coprococcus comes, Coprococcus catus |
| PEPTOSTREPTOCOCCUS | Peptostreptococcaceae noname unclassified, Eubacterium yurii, Clostridium sordellii, Clostridium hiranonis, Clostridium glycolicum, Clostridium difficile, Clostridium difficile, Clostridium bifermentans, Clostridium bartlettii |
| ERYSIPELOTRICHACEAE NONAME | Eubacterium dolichum, Eubacterium cylindroides, Eubacterium biforme, Erysipelotrichaceae bacterium 6 1 45, Erysipelotrichaceae bacterium 5 2 54FAA, Erysipelotrichaceae bacterium 3 1 53, Erysipelotrichaceae bacterium 21 3, Erysipelotrichaceae bacterium 2 2 44A, Clostridium spiroforme, Clostridium ramosum, Clostridium innocuum |
| GRANULICATELLA | Granulicatella unclassified, Granulicatella adiacens, Granulicatella elegans |

**Supplementary Table 9.** Species names identified for the top 10 family taxon groups for IBDMDB.

| **Name of Taxa for IBDMDB (Family)** | **Species Name** |
| --- | --- |
| BACTEROIDACEAE | Bacteroides xylanisolvens, Bacteroides vulgatus,, Bacteroides uniformis,, Bacteroides thetaiotaomicron,, Bacteroides stercoris,, Bacteroides stercorirosoris,, Bacteroides sp OM08 11,, Bacteroides sp D2, Bacteroides sp CAG 661, Bacteroides sp CAG 633, Bacteroides sp CAG 598, Bacteroides sp CAG 530, Bacteroides sp CAG 144, Bacteroides sp 43 108, Bacteroides sartorii, Bacteroides salyersiae, Bacteroides plebeius, Bacteroides ovatus, Bacteroides oleiciplenus, Bacteroides nordii, Bacteroides massiliensis, Bacteroides intestinalis, Bacteroides galacturonicus, Bacteroides fragilis, Bacteroides fluxus, Bacteroides finegoldii, Bacteroides faecis CAG 32, Bacteroides faecis, Bacteroides eggerthii, Bacteroides dorei, Bacteroides coprocola, Bacteroides clarus, Bacteroides cellulosilyticus, Bacteroides caccae |
| LACHNOSPIRACEAE | Tyzzerella nexilis, Shuttleworthia satelles, Sellimonas intestinalis, Ruminococcus torques, Ruminococcus gnavus, Roseburia sp CAG 471, Roseburia sp CAG 309, Roseburia sp CAG 303, Roseburia sp CAG 182, Roseburia inulinivorans, Roseburia intestinalis, Roseburia hominis, Roseburia faecis, Robinsoniella sp RHS, Murimonas intestini, Lachnospiraceae bacterium oral taxon 096, Lachnospiraceae bacterium 2 1 46FAA, Lachnospira pectinoschiza, Lachnoclostridium sp An298, Lachnoclostridium sp An181, Lachnoclostridium sp An14, Lachnoclostridium sp An138, Lachnoclostridium sp An131, Lachnoclostridium sp An118, Fusicatenibacter saccharivorans, Faecalicatena orotica, Eubacterium rectale, Eisenbergiella tayi, Eisenbergiella massiliensis, Dorea sp CAG 317, Dorea longicatena, Dorea formicigenerans, Coprococcus eutactus, Coprococcus comes, Coprococcus catus, Clostridium symbiosum, Clostridium scindens, Clostridium saccharolyticum, Clostridium lavalense, Clostridium hylemonae, Clostridium clostridioforme, Clostridium citroniae, Clostridium celerecrescens, Clostridium bolteae, Clostridium asparagiforme, Clostridium aldenense, Cellulosilyticum lentocellum, Butyrivibrio sp CAG 318, Butyrivibrio crossotus, Blautia wexlerae, Blautia sp N6H1 15, Blautia sp CAG 257, Blautia producta, Blautia obeum, Blautia hydrogenotrophica, Blautia hansenii, Blautia coccoides, Anaerotignum lactatifermentans, Anaerostipes sp 992a, Anaerostipes sp 494a, Anaerostipes hadrus, Anaerostipes caccae, Anaerosporobacter mobilis, Anaerocolumna aminovalerica |
| RUMINOCOCCACEAE | Ruthenibacterium lactatiformans, Ruminococcus sp CAG 563, Ruminococcus sp CAG 488, Ruminococcus sp CAG 403, Ruminococcus sp CAG 330, Ruminococcus obeum CAG 39, Ruminococcus lactaris, Ruminococcus champanellensis, Ruminococcus callidus, Ruminococcus bromii, Ruminococcus bicirculans, Ruminococcaceae bacterium D5, Ruminococcaceae bacterium D16, Pseudoflavonifractor sp An85, Pseudoflavonifractor sp An184, Pseudoflavonifractor capillosus, Harryflintia acetispora, Gemmiger sp An87, Gemmiger sp An50, Gemmiger formicilis, Flavonifractor sp An306, Flavonifractor sp An100, Flavonifractor sp An10, Flavonifractor plautii, Faecalibacterium prausnitzii, Eubacterium siraeum, Clostridium methylpentosum, Clostridium leptum, Anaerotruncus sp CAG 528, Anaerotruncus colihominis, Anaeromassilibacillus sp An250, Anaeromassilibacillus sp An172, Anaerofilum sp An201, Agathobaculum butyriciproducens |
| RIKENELLACEAE | Rikenella microfusus, Alistipes timonensis, Alistipes shahii, Alistipes putredinis, Alistipes onderdonkii, Alistipes inops, Alistipes indistinctus, Alistipes finegoldii |
| FIRMICUTES_UNCLASSIFIED | Firmicutes bacterium CAG 95, Firmicutes bacterium CAG 94, Firmicutes bacterium CAG 83, Firmicutes bacterium CAG 646, Firmicutes bacterium CAG 534, Firmicutes bacterium CAG 424, Firmicutes bacterium CAG 238, Firmicutes bacterium CAG 170, Firmicutes bacterium CAG 145, Firmicutes bacterium CAG 110 |
| TANNERELLACEAE | Parabacteroides sp CAG 409, Parabacteroides merdae, Parabacteroides johnsonii, Parabacteroides goldsteinii, Parabacteroides distasonis |
| EUBACTERIACEAE | Eubacterium ventriosum, Eubacterium sp OM08 24, Eubacterium sp CAG 38, Eubacterium sp CAG 274, Eubacterium sp CAG 251, Eubacterium sp CAG 180, Eubacterium sp An11, Eubacterium ramulus, Eubacterium limosum, Eubacterium hallii, Eubacterium eligens, Eubacterium dolichum CAG 375, Eubacterium coprostanoligenes, Eubacteriaceae bacterium CHKCI005, Anaerofustis stercorihominis |
| CLOSTRIDIACEAE | Lactonifactor longoviformis, Hungatella hathewayi, Clostridium ventriculi, Clostridium sporogenes, Clostridium sp MSTE9, Clostridium sp D5, Clostridium sp chh4 2, Clostridium sp CAG 964, Clostridium sp CAG 678, Clostridium sp CAG 590, Clostridium sp CAG 58, Clostridium sp CAG 413, Clostridium sp CAG 411, Clostridium sp CAG 299, Clostridium sp CAG 253, Clostridium sp CAG 242, Clostridium sp CAG 167, Clostridium sp 7 2 43FAA, Clostridium perfringens, Clostridium paraputrificum, Clostridium neonatale, Clostridium disporicum, Clostridium celatum, Clostridium cadaveris, Clostridium butyricum, Clostridium botulinum, Clostridium bolteae CAG 59, Clostridium baratii, Butyricicoccus pullicaecorum, Butyribacterium methylotrophicum |
| VEILLONELLACEAE | Veillonella tobetsuensis, Veillonella sp T11011 6, Veillonella sp CAG 933, Veillonella seminalis, Veillonella rogosae, Veillonella rodentium, Veillonella parvula, Veillonella infantium, Veillonella dispar, Veillonella atypica, Megasphaera sp MJR8396C, Megasphaera sp DISK 18, Megasphaera micronuciformis, Megasphaera elsdenii, Dialister sp CAG 357, Dialister pneumosintes, Dialister micraerophilus, Dialister invisus, Anaeroglobus geminatus, Allisonella histaminiformans |
| ODORIBACTERACEAE | Odoribacter splanchnicus, Odoribacter laneus, Butyricimonas virosa, Butyricimonas synergistica |

**Supplementary Table 10.** Species name identified for the top 10 order taxon groups for IBDMDB.

| **Name of Taxa for IBDMDB (Order)** | **Species Name** |
| --- | --- |
| BACTEROIDALES | Sanguibacteroides justesenii, Rikenella microfusus, Prevotella timonensis, Prevotella stercorea, Prevotella sp S7 1 8, Prevotella sp CAG 891, Prevotella sp CAG 520, Prevotella sp CAG 279, Prevotella sp CAG 1185, Prevotella sp CAG 1092, Prevotella sp AM42 24, Prevotella sp 885, Prevotella salivae, Prevotella pallens, Prevotella oris, Prevotella oralis, Prevotella nigrescens, Prevotella melaninogenica, Prevotella jejuni, Prevotella intermedia, Prevotella histicola, Prevotella disiens, Prevotella denticola, Prevotella dentalis, Prevotella corporis, Prevotella copri, Prevotella colorans, Prevotella buccalis, Prevotella buccae, Prevotella bivia, Prevotella bergensis, Prevotella amnii, Porphyromonas uenonis, Porphyromonas sp HMSC065F10, Porphyromonas somerae, Porphyromonas endodontalis, Porphyromonas asaccharolytica, Paraprevotella xylaniphila, Paraprevotella clara, Parabacteroides sp CAG 409, Parabacteroides merdae, Parabacteroides johnsonii, Parabacteroides goldsteinii, Parabacteroides distasonis, Odoribacter splanchnicus, Odoribacter laneus, Dysgonomonas sp 37 18, Dysgonomonas mossii, Dysgonomonas gadei, Coprobacter sp, Coprobacter secundus, Coprobacter fastidiosus, Butyricimonas virosa, Butyricimonas synergistica, Barnesiella intestinihominis, Bacteroides xylanisolvens, Bacteroides vulgatus, Bacteroides uniformis, Bacteroides thetaiotaomicron, Bacteroides stercoris, Bacteroides stercorirosoris, Bacteroides sp OM08 11, Bacteroides sp D2, Bacteroides sp CAG 661, Bacteroides sp CAG 633, Bacteroides sp CAG 598, Bacteroides sp CAG 530, Bacteroides sp CAG 144, Bacteroides sp 43 108, Bacteroides sartorii, Bacteroides salyersiae, Bacteroides plebeius, Bacteroides ovatus, Bacteroides oleiciplenus, Bacteroides nordii, Bacteroides massiliensis, Bacteroides intestinalis, Bacteroides galacturonicus, Bacteroides fragilis, Bacteroides fluxus, Bacteroides finegoldii, Bacteroides faecis CAG 32, Bacteroides faecis, Bacteroides eggerthii, Bacteroides dorei, Bacteroides coprocola, Bacteroides clarus, Bacteroides cellulosilyticus, Bacteroides caccae, Bacteroidales bacterium KA00251, Alloprevotella tannerae, Alistipes timonensis, Alistipes shahii, Alistipes putredinis, Alistipes onderdonkii, Alistipes inops, Alistipes indistinctus, Alistipes finegoldii |
| CLOSTRIDIALES | Tyzzerella nexilis, Terrisporobacter othiniensis, Shuttleworthia satelles, Sellimonas intestinalis, Ruthenibacterium lactatiformans, Ruminococcus torques, Ruminococcus sp CAG 563, Ruminococcus sp CAG 488, Ruminococcus sp CAG 403, Ruminococcus sp CAG 330, Ruminococcus obeum CAG 39, Ruminococcus lactaris, Ruminococcus gnavus, Ruminococcus champanellensis, Ruminococcus callidus, Ruminococcus bromii, Ruminococcus bicirculans, Ruminococcaceae bacterium D5, Ruminococcaceae bacterium D16, Roseburia sp CAG 471, Roseburia sp CAG 309, Roseburia sp CAG 303, Roseburia sp CAG 182, Roseburia inulinivorans, Roseburia intestinalis, Roseburia hominis, Roseburia faecis, Romboutsia ilealis, Robinsoniella sp RHS, Pseudoflavonifractor sp An85, Pseudoflavonifractor sp An184, Pseudoflavonifractor capillosus, Peptostreptococcus stomatis, Peptostreptococcus anaerobius, Peptococcus niger, Oscillibacter sp PC13, Oscillibacter sp CAG 241, Oscillibacter sp 57 20, Murimonas intestini, Monoglobus pectinilyticus, Lawsonibacter asaccharolyticus, Lactonifactor longoviformis, Lachnospiraceae bacterium oral taxon 096, Lachnospiraceae bacterium 2 1 46FAA, Lachnospira pectinoschiza, Lachnoclostridium sp An298, Lachnoclostridium sp An181, Lachnoclostridium sp An14, Lachnoclostridium sp An138, Lachnoclostridium sp An131, Lachnoclostridium sp An118, Intestinimonas butyriciproducens, Intestinibacter bartlettii, Hungatella hathewayi, Harryflintia acetispora, Gemmiger sp An87, Gemmiger sp An50, Gemmiger formicilis, Fusicatenibacter saccharivorans, Flavonifractor sp An306, Flavonifractor sp An100, Flavonifractor sp An10, Flavonifractor plautii, Faecalicatena orotica, Faecalibacterium prausnitzii, Eubacterium ventriosum, Eubacterium sp OM08 24, Eubacterium sp CAG 38, Eubacterium sp CAG 274, Eubacterium sp CAG 251, Eubacterium sp CAG 180, Eubacterium sp An11, Eubacterium siraeum, Eubacterium rectale, Eubacterium ramulus, Eubacterium limosum, Eubacterium infirmum, Eubacterium hallii, Eubacterium eligens, Eubacterium dolichum CAG 375, Eubacterium coprostanoligenes, Eubacterium brachy, Eubacteriaceae bacterium CHKCI005, Eisenbergiella tayi, Eisenbergiella massiliensis, Dorea sp CAG 317, Dorea longicatena, Dorea formicigenerans, Coprococcus eutactus, Coprococcus comes, Coprococcus catus, Clostridium ventriculi, Clostridium symbiosum, Clostridium sporogenes, Clostridium sp MSTE9, Clostridium sp D5, Clostridium sp chh4 2, Clostridium sp CAG 964, Clostridium sp CAG 678, Clostridium sp CAG 590, Clostridium sp CAG 58, Clostridium sp CAG 413, Clostridium sp CAG 411, Clostridium sp CAG 299, Clostridium sp CAG 253, Clostridium sp CAG 242, Clostridium sp CAG 167, Clostridium sp 7 2 43FAA, Clostridium scindens, Clostridium saccharolyticum, Clostridium perfringens, Clostridium paraputrificum, Clostridium neonatale, Clostridium methylpentosum, Clostridium leptum, Clostridium lavalense, Clostridium hylemonae, Clostridium disporicum, Clostridium clostridioforme, Clostridium citroniae, Clostridium celerecrescens, Clostridium celatum, Clostridium cadaveris, Clostridium butyricum, Clostridium botulinum, Clostridium bolteae CAG 59, Clostridium bolteae, Clostridium baratii, Clostridium asparagiforme, Clostridium aldenense, Clostridioides difficile, Clostridiales bacterium CHKCI006, Clostridiales bacterium 1 7 47FAA, Christensenella minuta, Cellulosilyticum lentocellum, Catabacter hongkongensis, Butyrivibrio sp CAG 318, Butyrivibrio crossotus, Butyricicoccus pullicaecorum, Butyribacterium methylotrophicum, Blautia wexlerae, Blautia sp N6H1 15, Blautia sp CAG 257, Blautia producta, Blautia obeum, Blautia hydrogenotrophica, Blautia hansenii, Blautia coccoides, Bacteroides pectinophilus, Anaerotruncus sp CAG 528, Anaerotruncus colihominis, Anaerotignum lactatifermentans, Anaerostipes sp 992a, Anaerostipes sp 494a, Anaerostipes hadrus, Anaerostipes caccae, Anaerosporobacter mobilis, Anaeromassilibacillus sp An250, Anaeromassilibacillus sp An172, Anaerofustis stercorihominis, Anaerofilum sp An201, Anaerocolumna aminovalerica, Agathobaculum butyriciproducens |
| FIRMICUTES_UNCLASSIFIED | Firmicutes bacterium CAG 95, Firmicutes bacterium CAG 94, Firmicutes bacterium CAG 83, Firmicutes bacterium CAG 646, Firmicutes bacterium CAG 534, Firmicutes bacterium CAG 424, Firmicutes bacterium CAG 238, Firmicutes bacterium CAG 170, Firmicutes bacterium CAG 145, Firmicutes bacterium CAG 110 |
| VEILLONELLALES | Veillonella tobetsuensis, Veillonella sp T11011 6, Veillonella sp CAG 933, Veillonella seminalis, Veillonella rogosae, Veillonella rodentium, Veillonella parvula, Veillonella infantium, Veillonella dispar, Veillonella atypica, Megasphaera sp MJR8396C, Megasphaera sp DISK 18, Megasphaera micronuciformis, Megasphaera elsdenii, Dialister sp CAG 357, Dialister pneumosintes, Dialister micraerophilus, Dialister invisus, Anaeroglobus geminatus, Allisonella histaminiformans |
| BURKHOLDERIALES | Turicimonas muris, Sutterella parvirubra, Parasutterella excrementihominis, Oxalobacter formigenes |
| METHANOMASSILIICOCCALES | Candidatus Methanomassiliicoccus intestinalis |
| DESULFOVIBRIONALES | Desulfovibrionaceae bacterium, Desulfovibrio piger, Desulfovibrio fairfieldensis, Bilophila wadsworthia |
| ERYSIPELOTRICHALES | Turicibacter sanguinis, Solobacterium moorei, Massiliomicrobiota timonensis, Holdemania filiformis, Holdemanella biformis, Faecalitalea cylindroides, Faecalicoccus pleomorphus, Erysipelothrix larvae, Erysipelatoclostridium ramosum, Dielma fastidiosa, Coprobacillus cateniformis, Clostridium spiroforme, Clostridium innocuum, Catenibacterium mitsuokai, Candidatus Stoquefichus sp KLE1796, Bulleidia extructa, Absiella dolichum |
| BIFIDOBACTERIALES | Scardovia wiggsiae, Bifidobacterium saeculare, Bifidobacterium pullorum, Bifidobacterium pseudolongum, Bifidobacterium pseudocatenulatum, Bifidobacterium longum, Bifidobacterium gallinarum, Bifidobacterium dentium, Bifidobacterium catenulatum, Bifidobacterium breve, Bifidobacterium bifidum, Bifidobacterium asteroides, Bifidobacterium animalis, Bifidobacterium angulatum, Bifidobacterium adolescentis, Alloscardovia omnicolens, Aeriscardovia aeriphila |
| EGGERTHELLALES | Slackia isoflavoniconvertens, Gordonibacter pamelaeae, Enterorhabdus caecimuris, Eggerthella lenta, Asaccharobacter celatus, Adlercreutzia equolifaciens |

**Supplementary Table 11.** Species name identified for the top 10 genus taxon groups for IBDMDB.

| **Name of Taxa for IBDMDB (Genus)** | **Species Name** |
| --- | --- |
| BACTEROIDES | Bacteroides xylanisolvens, Bacteroides vulgatus, Bacteroides uniformis, Bacteroides thetaiotaomicron, Bacteroides stercoris, Bacteroides stercorirosoris, Bacteroides sp OM08 11, Bacteroides sp D2, Bacteroides sp CAG 661, Bacteroides sp CAG 633, Bacteroides sp CAG 598, Bacteroides sp CAG 530, Bacteroides sp CAG 144, Bacteroides sp 43 108, Bacteroides sartorii, Bacteroides salyersiae, Bacteroides plebeius, Bacteroides ovatus, Bacteroides oleiciplenus, Bacteroides nordii, Bacteroides massiliensis, Bacteroides intestinalis, Bacteroides galacturonicus, Bacteroides fragilis, Bacteroides fluxus, Bacteroides finegoldii, Bacteroides faecis CAG 32, Bacteroides faecis, Bacteroides eggerthii, Bacteroides dorei, Bacteroides coprocola, Bacteroides clarus, Bacteroides cellulosilyticus, Bacteroides caccae |
| ALISTIPES | Alistipes timonensis, Alistipes shahii, Alistipes putredinis, Alistipes onderdonkii, Alistipes inops, Alistipes indistinctus, Alistipes finegoldii |
| EUBACTERIUM | Eubacterium ventriosum, Eubacterium sp OM08 24, Eubacterium sp CAG 38, Eubacterium sp CAG 274, Eubacterium sp CAG 251, Eubacterium sp CAG 180, Eubacterium sp An11, Eubacterium ramulus, Eubacterium limosum, Eubacterium hallii, Eubacterium eligens, Eubacterium dolichum CAG 375, Eubacterium coprostanoligenes |
| ROSEBURIA | Roseburia sp CAG 471, Roseburia sp CAG 309, Roseburia sp CAG 303, Roseburia sp CAG 182, Roseburia inulinivorans, Roseburia intestinalis, Roseburia hominis, Roseburia faecis |
| FIRMICUTES_UNCLASSIFIED | Firmicutes bacterium CAG 95, Firmicutes bacterium CAG 94, Firmicutes bacterium CAG 83, Firmicutes bacterium CAG 646, Firmicutes bacterium CAG 534, Firmicutes bacterium CAG 424, Firmicutes bacterium CAG 238, Firmicutes bacterium CAG 170, Firmicutes bacterium CAG 145, Firmicutes bacterium CAG 110 |
| PARABACTEROIDES | Parabacteroides sp CAG 409, Parabacteroides merdae, Parabacteroides johnsonii, Parabacteroides goldsteinii, Parabacteroides distasonis |
| RUMINOCOCCUS | Ruminococcus sp CAG 563, Ruminococcus sp CAG 488, Ruminococcus sp CAG 403, Ruminococcus sp CAG 330, Ruminococcus obeum CAG 39, Ruminococcus lactaris, Ruminococcus champanellensis, Ruminococcus callidus, Ruminococcus bromii, Ruminococcus bicirculans |
| COPROCOCCUS | Coprococcus catus, Coprococcus comes, Coprococcus eutactus |
| BLAUTIA | Ruminococcus torques, Ruminococcus gnavus, Blautia wexlerae, Blautia sp N6H1 15, Blautia sp CAG 257, Blautia producta, Blautia obeum, Blautia hydrogenotrophica, Blautia hansenii, Blautia coccoides |
| CLOSTRIDIUM | Clostridium ventriculi, Clostridium sporogenes, Clostridium sp MSTE9, Clostridium sp D5, Clostridium sp chh4 2, Clostridium sp CAG 964, Clostridium sp CAG 678, Clostridium sp CAG 590, Clostridium sp CAG 58, Clostridium sp CAG 413, Clostridium sp CAG 411, Clostridium sp CAG 299, Clostridium sp CAG 253, Clostridium sp CAG 242, Clostridium sp CAG 167, Clostridium sp 7 2 43FAA, Clostridium perfringens, Clostridium paraputrificum, Clostridium neonatale, Clostridium disporicum, Clostridium celatum, Clostridium cadaveris, Clostridium butyricum, Clostridium botulinum, Clostridium bolteae CAG 59, Clostridium baratii, Butyribacterium methylotrophicum |

**Supplementary Table 12.** Species name identified for the top 10 family taxon groups for CRC.

| **Name of Taxa for CRC (Family)** | **Species Name** |
| --- | --- |
| PEPTOSTREPTOCOCCACEAE | Clostridioides_difficile, Criibacterium_bergeronii, Filifactor_alocis, Intestinibacter_bartlettii, Paeniclostridium_sordellii, Paraclostridium_bifermentans, Eubacterium_yurii, Peptoanaerobacter_stomatis, Clostridium_hiranonis, Peptostreptococcus_anaerobius, Peptostreptococcus_sp_MV1, Peptostreptococcus_stomatis, Romboutsia_ilealis, Terrisporobacter_othiniensis |
| PEPTONIPHILACEAE | Anaerococcus_lactolyticus, Anaerococcus_tetradius, Anaerococcus_vaginalis, Finegoldia_magna, Parvimonas_micra, Parvimonas_sp_KA00067, Parvimonas_sp_oral_taxon_110, Parvimonas_sp_oral_taxon_393, Peptoniphilus_coxii, Peptoniphilus_duerdenii, Peptoniphilus_grossensis, Peptoniphilus_harei, Peptoniphilus_lacrimalis, Peptoniphilus_sp_BV3C26, Peptoniphilus_sp_HMSC062D09 |
| FUSOBACTERIACEAE | Cetobacterium_somerae, Fusobacterium_equinum, Fusobacterium_gonidiaformans, Fusobacterium_hwasookii, Fusobacterium_mortiferum, Fusobacterium_naviforme, Fusobacterium_necrophorum, Fusobacterium_nucleatum, Fusobacterium_periodonticum, Fusobacterium_sp_CAG_439, Fusobacterium_sp_oral_taxon_370, Fusobacterium_ulcerans, Fusobacterium_varium |
| BACILLALES_UNCLASSIFIED | Gemella_asaccharolytica,Gemella_bergeri,Gemella_haemolysans,Gemella_morbillorum,Gemella_sanguinis |
| VEILLONELLACEAE | Allisonella_histaminiformans, Anaeroglobus_geminatus, Dialister_invisus, Dialister_micraerophilus, Dialister_pneumosintes, Dialister_sp_CAG_357, Dialister_succinatiphilus, Megasphaera_cerevisiae, Megasphaera_elsdenii, Megasphaera_hexanoica, Megasphaera_micronuciformis, Megasphaera_sp_BV3C16_1, Megasphaera_sp_DISK_18, Megasphaera_sp_MJR8396C, Megasphaera_stantonii, Negativicoccus_succinicivorans, Veillonella_atypica, Veillonella_denticariosi, Veillonella_dispar, Veillonella_infantium, Veillonella_parvula, Veillonella_rodentium, Veillonella_rogosae, Veillonella_sp_CAG_933, Veillonella_sp_T11011_6, Veillonella_tobetsuensis |
| LACHNOSPIRACEAE | Anaerocolumna_aminovalerica, Anaerosporobacter_mobilis, Anaerostipes_caccae, Anaerostipes_hadrus, Anaerostipes_sp_494a, Anaerotignum_lactatifermentans, Blautia_coccoides, Blautia_hansenii, Blautia_hydrogenotrophica, Blautia_obeum, Blautia_producta, Blautia_sp_An249, Blautia_sp_CAG_257, Blautia_sp_N6H1_15, Blautia_wexlerae, Ruminococcus_gnavus, Ruminococcus_torques, Butyrivibrio_crossotus, Butyrivibrio_sp_CAG_318, Catonella_morbi, Cellulosilyticum_lentocellum, Coprococcus_catus, Coprococcus_comes, Coprococcus_eutactus, Dorea_formicigenerans, Dorea_longicatena, Dorea_sp_CAG_317, Dorea_sp_D27, Eisenbergiella_massiliensis, Eisenbergiella_tayi, Faecalicatena_orotica, Fusicatenibacter_saccharivorans, Lachnoanaerobaculum_saburreum, Lachnoanaerobaculum_umeaense, Clostridium_aldenense, Clostridium_asparagiforme, Clostridium_bolteae, Clostridium_citroniae, Clostridium_clostridioforme, Clostridium_hylemonae, Clostridium_lavalense, Clostridium_saccharolyticum, Clostridium_scindens, Clostridium_symbiosum, Lachnoclostridium_sp_An118, Lachnoclostridium_sp_An131, Lachnoclostridium_sp_An138, Lachnoclostridium_sp_An14, Lachnoclostridium_sp_An169, Lachnoclostridium_sp_An196, Lachnoclostridium_sp_An298, Lachnospira_pectinoschiza, Lachnospiraceae_bacterium_2_1_46FAA, Lachnospiraceae_bacterium_oral_taxon_096, Marvinbryantia_formatexigens, Murimonas_intestini, Oribacterium_asaccharolyticum, Oribacterium_parvum, Oribacterium_sinus, Oribacterium_sp_oral_taxon_078, Robinsoniella_sp_RHS, Roseburia_faecis, Roseburia_hominis, Roseburia_intestinalis, Roseburia_inulinivorans, Roseburia_sp_831b, Roseburia_sp_CAG_182, Roseburia_sp_CAG_303, Roseburia_sp_CAG_309, Roseburia_sp_CAG_471, Sellimonas_intestinalis, Shuttleworthia_satelles, Stomatobaculum_longum, Tyzzerella_nexilis, Tyzzerella_sp_An114 |
| ERYSIPELOTRICHACEAE | Absiella_dolichum, Bulleidia_extructa, Candidatus_Stoquefichus_sp_KLE1796, Catenibacterium_mitsuokai, Coprobacillus_cateniformis, Dielma_fastidiosa, Eggerthia_catenaformis, Clostridium_innocuum, Clostridium_spiroforme, Erysipelatoclostridium_ramosum, Erysipelothrix_larvae, Faecalicoccus_pleomorphus, Faecalitalea_cylindroides, Holdemanella_biformis, Holdemania_filiformis, Massiliomicrobiota_timonensis, Solobacterium_moorei, Turicibacter_sanguinis |
| RUMINOCOCCACEAE | Acetivibrio_ethanolgignens, Agathobaculum_butyriciproducens, Anaerofilum_sp_An201, Anaeromassilibacillus_sp_An172, Anaeromassilibacillus_sp_An250, Anaerotruncus_colihominis, Anaerotruncus_sp_CAG_528, Faecalibacterium_prausnitzii, Flavonifractor_plautii, Flavonifractor_sp_An10, Flavonifractor_sp_An100, Flavonifractor_sp_An306, Flavonifractor_sp_An82, Gemmiger_formicilis, Gemmiger_sp_An120, Gemmiger_sp_An194, Gemmiger_sp_An50, Gemmiger_sp_An87, Harryflintia_acetispora, Pseudoflavonifractor_capillosus, Pseudoflavonifractor_sp_An184, Clostridium_leptum (Ruminococcaceae_unclassified), Clostridium_methylpentosum (Ruminococcaceae_unclassified), Eubacterium_siraeum (Ruminococcaceae_unclassified), Ruminococcaceae_bacterium_D16 (Ruminococcaceae_unclassified), Ruminococcus_bicirculans, Ruminococcus_bromii, Ruminococcus_callidus, Ruminococcus_champanellensis, Ruminococcus_lactaris, Ruminococcus_obeum_CAG_39, Ruminococcus_sp_CAG_330, Ruminococcus_sp_CAG_403, Ruminococcus_sp_CAG_488, Ruminococcus_sp_CAG_563, Ruminococcus_sp_CAG_579, Ruminococcus_sp_CAG_624, Ruthenibacterium_lactatiformans, Subdoligranulum_variabile |
| PREVOTELLACEAE | Alloprevotella_rava, Alloprevotella_tannerae, Prevotella_sp_oral_taxon_473, Paraprevotella_clara, Paraprevotella_xylaniphila, Prevotella_amnii, Prevotella_baroniae, Prevotella_bergensis, Prevotella_bivia, Prevotella_buccae, Prevotella_buccalis, Prevotella_colorans, Prevotella_copri, Prevotella_corporis, Prevotella_dentalis, Prevotella_denticola, Prevotella_disiens, Prevotella_histicola, Prevotella_intermedia, Prevotella_jejuni, Prevotella_melaninogenica, Prevotella_multiformis, Prevotella_multisaccharivorax, Prevotella_nigrescens, Prevotella_oralis, Prevotella_oris, Prevotella_oulorum, Prevotella_pallens, Prevotella_salivae, Prevotella_scopos, Prevotella_sp_885, Prevotella_sp_AM42_24, Prevotella_sp_CAG_1031, Prevotella_sp_CAG_1058, Prevotella_sp_CAG_1092, Prevotella_sp_CAG_1124, Prevotella_sp_CAG_1185, Prevotella_sp_CAG_1320, Prevotella_sp_CAG_279, Prevotella_sp_CAG_485, Prevotella_sp_CAG_520, Prevotella_sp_CAG_5226, Prevotella_sp_CAG_617, Prevotella_sp_CAG_755, Prevotella_sp_CAG_873, Prevotella_sp_CAG_891, Prevotella_sp_CAG_924, Prevotella_sp_F0091, Prevotella_sp_oral_taxon_306, Prevotella_sp_oral_taxon_376, Prevotella_sp_P3_122, Prevotella_sp_S7_1_8, Prevotella_stercorea, Prevotella_timonensis, Prevotella_veroralis |
| STREPTOCOCCACEAE | Lactococcus_garvieae, Lactococcus_lactis, Lactococcus_petauri, Lactococcus_piscium, Lactococcus_plantarum, Lactococcus_raffinolactis, Streptococcus_agalactiae, Streptococcus_anginosus_group, Streptococcus_australis, Streptococcus_cristatus, Streptococcus_downei, Streptococcus_equinus, Streptococcus_gallolyticus, Streptococcus_gordonii, Streptococcus_infantarius, Streptococcus_infantis, Streptococcus_lutetiensis, Streptococcus_macedonicus, Streptococcus_massiliensis, Streptococcus_milleri, Streptococcus_mitis, Streptococcus_mutans, Streptococcus_oralis, Streptococcus_parasanguinis, Streptococcus_parauberis, Streptococcus_pasteurianus, Streptococcus_peroris, Streptococcus_pneumoniae, Streptococcus_pseudopneumoniae, Streptococcus_salivarius, Streptococcus_salivarius_CAG_79, Streptococcus_sanguinis, Streptococcus_sinensis, Streptococcus_sobrinus, Streptococcus_sp_A12, Streptococcus_sp_F0442, Streptococcus_sp_HMSC034E03, Streptococcus_sp_HMSC067H01, Streptococcus_sp_HMSC070B10, Streptococcus_sp_HMSC071D03, Streptococcus_sp_HPH0090, Streptococcus_sp_M334, Streptococcus_sp_NLAE_zl_C503, Streptococcus_sp_oral_taxon_056, Streptococcus_sp_oral_taxon_058, Streptococcus_sp_SK643, Streptococcus_thermophilus, Streptococcus_vestibularis, Streptococcus_viridans |

**Supplementary Table 13.** Species name identified for the top 10 order taxon groups for CRC.

| **Name of Taxa for CRC (Order)** | **Species Name** |
| --- | --- |
| CLOSTRIDIALES | Catabacter_hongkongensis, Christensenella_minuta, Butyricicoccus_pullicaecorum, Butyribacterium_methylotrophicum, Clostridium_baratii, Clostridium_bolteae_CAG_59, Clostridium_butyricum, Clostridium_cadaveris, Clostridium_celatum, Clostridium_disporicum, Clostridium_neonatale, Clostridium_paraputrificum, Clostridium_perfringens, Clostridium_sp_7_2_43FAA, Clostridium_sp_CAG_167, Clostridium_sp_CAG_242, Clostridium_sp_CAG_253, Clostridium_sp_CAG_299, Clostridium_sp_CAG_411, Clostridium_sp_CAG_413, Clostridium_sp_CAG_510, Clostridium_sp_CAG_58, Clostridium_sp_CAG_590, Clostridium_sp_CAG_632, Clostridium_sp_CAG_678, Clostridium_sp_CAG_964, Clostridium_sp_chh4_2, Clostridium_sp_D5, Clostridium_sp_MSTE9, Clostridium_sp_SY8519, Clostridium_ventriculi, Hungatella_effluvii, Hungatella_hathewayi, Lactonifactor_longoviformis, Eubacterium_brachy, Eubacterium_infirmum, Eubacterium_nodatum, Eubacterium_saphenum, Eubacterium_sulci, Mogibacterium_diversum, Mogibacterium_pumilum, Mogibacterium_timidum, Bacteroides_pectinophilus, Clostridiales_bacterium_1_7_47FAA, Clostridiales_bacterium_CHKCI006, Intestinimonas_butyriciproducens, Lawsonibacter_asaccharolyticus, Monoglobus_pectinilyticus, Anaerofustis_stercorihominis, Eubacteriaceae_bacterium_CHKCI005, Eubacterium_callanderi, Eubacterium_coprostanoligenes, Eubacterium_dolichum_CAG_375, Eubacterium_eligens, Eubacterium_hallii, Eubacterium_limosum, Eubacterium_ramulus, Eubacterium_sp_An11, Eubacterium_sp_CAG_180, Eubacterium_sp_CAG_251, Eubacterium_sp_CAG_274, Eubacterium_sp_CAG_38, Eubacterium_sp_CAG_786, Eubacterium_sp_OM08_24, Eubacterium_ventriosum, Pseudoramibacter_alactolyticus, Mageeibacillus_indolicus, Anaerocolumna_aminovalerica, Anaerosporobacter_mobilis, Anaerostipes_caccae, Anaerostipes_hadrus, Anaerostipes_sp_494a, Anaerotignum_lactatifermentans, Blautia_coccoides, Blautia_hansenii, Blautia_hydrogenotrophica, Blautia_obeum, Blautia_producta, Blautia_sp_An249, Blautia_sp_CAG_257, Blautia_sp_N6H1_15, Blautia_wexlerae, Ruminococcus_gnavus, Ruminococcus_torques, Butyrivibrio_crossotus, Butyrivibrio_sp_CAG_318, Catonella_morbi, Cellulosilyticum_lentocellum, Coprococcus_catus, Coproc |
| TISSIERELLALES | Anaerococcus_lactolyticus, Anaerococcus_tetradius, Anaerococcus_vaginalis, Finegoldia_magna, Parvimonas_micra, Parvimonas_sp_KA00067, Parvimonas_sp_oral_taxon_110, Parvimonas_sp_oral_taxon_393, Peptoniphilus_coxii, Peptoniphilus_duerdenii, Peptoniphilus_grossensis, Peptoniphilus_harei, Peptoniphilus_lacrimalis, Peptoniphilus_sp_BV3C26, Peptoniphilus_sp_HMSC062D09 |
| BACTEROIDALES | Bacteroides caccae, Bacteroides caecimuris, Bacteroides cellulosilyticus, Bacteroides clarus, Bacteroides coprocola, Bacteroides coprophilus, Bacteroides dorei, Bacteroides eggerthii, Bacteroides faecichinchillae, Bacteroides faecis, Bacteroides faecis CAG 32, Bacteroides finegoldii, Bacteroides fluxus, Bacteroides fragilis, Bacteroides galacturonicus, Bacteroides heparinolyticus, Bacteroides intestinalis, Bacteroides massiliensis, Bacteroides nordii, Bacteroides oleiciplenus, Bacteroides ovatus, Bacteroides plebeius, Bacteroides pyogenes, Bacteroides salanitronis, Bacteroides salyersiae, Bacteroides sartorii, Bacteroides sp 43 108, Bacteroides sp CAG 144, Bacteroides sp CAG 443, Bacteroides sp CAG 462, Bacteroides sp CAG 530, Bacteroides sp CAG 598, Bacteroides sp CAG 633, Bacteroides sp CAG 661, Bacteroides sp CAG 927, Bacteroides sp D2, Bacteroides sp OM05 12, Bacteroides sp OM08 11, Bacteroides stercorirosoris, Bacteroides stercoris, Bacteroides thetaiotaomicron, Bacteroides uniformis, Bacteroides vulgatus, Bacteroides xylanisolvens, Bacteroidales bacterium KA00251, Barnesiella intestinihominis, Barnesiella sp An22, Barnesiella viscericola, Coprobacter fastidiosus, Coprobacter secundus, Coprobacter sp, Dysgonomonas gadei, Dysgonomonas sp 37 18, Muribaculaceae bacterium DSM 103720, Muribaculum intestinale, Butyricimonas synergistica, Butyricimonas virosa, Odoribacter laneus, Odoribacter splanchnicus, Porphyromonas asaccharolytica, Porphyromonas canoris, Porphyromonas endodontalis, Porphyromonas gingivalis, Porphyromonas somerae, Porphyromonas sp HMSC065F10, Porphyromonas uenonis, Sanguibacteroides justesenii, Alloprevotella rava, Alloprevotella tannerae, Prevotella sp oral taxon 473, Paraprevotella clara, Paraprevotella xylaniphila, Prevotella amnii, Prevotella baroniae, Prevotella bergensis, Prevotella bivia, Prevotella buccae, Prevotella buccalis, Prevotella colorans, Prevotella copri, Prevotella corporis, Prevotella dentalis, Prevotella denticola, Prevotella disiens, Prevotella histicola, Prevotella intermedia, Prevotella jejuni, Prevotella melaninogenica, Prevotella multisaccharivorax, Prevotella nigrescens, Prevotella oralis, Prevotella oris, Prevotella oulorum, Prevotella pallens, Prevotella salivae, Prevotella scopos, Prevotella sp 885, Prevotella sp AM42 24, Prevotella sp CAG |
| FUSOBACTERIALES | Cetobacterium somerae, Fusobacterium equinum, Fusobacterium gonidiaformans, Fusobacterium hwasookii, Fusobacterium mortiferum, Fusobacterium naviforme, Fusobacterium necrophorum, Fusobacterium nucleatum, Fusobacterium periodonticum, Fusobacterium sp CAG 439, Fusobacterium sp oral taxon 370, Fusobacterium ulcerans, Fusobacterium varium, Leptotrichia hofstadii, Leptotrichia sp oral taxon 212, Leptotrichia sp oral taxon 215, Leptotrichia sp oral taxon 225, Leptotrichia wadei, Sneathia amnii |
| BACILLALES | Bacillus aerius, Bacillus sp FJAT 27916, Gemella asaccharolytica, Gemella bergeri, Gemella haemolysans, Gemella morbillorum, Gemella sanguinis, Macrococcus caseolyticus, Staphylococcus aureus, Staphylococcus carnosus, Staphylococcus epidermidis, Staphylococcus equorum, Staphylococcus haemolyticus, Staphylococcus saprophyticus, Staphylococcus warneri, Staphylococcus xylosus |
| VEILLONELLALES | Allisonella histaminiformans, Anaeroglobus geminatus, Dialister invisus, Dialister micraerophilus, Dialister pneumosintes, Dialister sp CAG 357, Dialister succinatiphilus, Megasphaera cerevisiae, Megasphaera elsdenii, Megasphaera hexanoica, Megasphaera micronuciformis, Megasphaera sp BV3C16 1, Megasphaera sp DISK 18, Megasphaera sp MJR8396C, Megasphaera stantonii, Negativicoccus succinicivorans, Veillonella atypica, Veillonella denticariosi, Veillonella dispar, Veillonella infantium, Veillonella parvula, Veillonella rodentium, Veillonella rogosae, Veillonella sp CAG 933, Veillonella sp T11011 6, Veillonella tobetsuensis |
| ERYSIPELOTRICHALES | Absiella dolichum, Bulleidia extructa, Candidatus_Stoquefichus_sp_KLE1796, Catenibacterium mitsuokai, Coprobacillus_cateniformis, Dielma fastidiosa, Eggerthia catenaformis, Clostridium_innocuum, Clostridium_spiroforme, Erysipelatoclostridium_ramosum, Erysipelothrix_larvae, Faecalicoccus_pleomorphus, Faecalitalea_cylindroides, Holdemanella_biformis, Holdemania_filiformis, Massiliomicrobiota_timonensis, Solobacterium_moorei, Turicibacter_sanguinis |
| LACTOBACILLALES | Abiotrophia_defectiva, Abiotrophia_sp_HMSC24B09, Eremococcus_coleocola, Facklamia_hominis, Carnobacterium_divergens, Carnobacterium_maltaromaticum, Dolosigranulum_pigrum, Granulicatella_adiacens, Granulicatella_elegans, Enterococcus_asini, Enterococcus_avium, Enterococcus_canis, Enterococcus_casseliflavus, Enterococcus_devriesei, Enterococcus_dispar, Enterococcus_durans, Enterococcus_faecalis, Enterococcus_faecium, Enterococcus_gallinarum, Enterococcus_gilvus, Enterococcus_hirae, Enterococcus_italicus, Enterococcus_malodoratus, Enterococcus_mundtii, Enterococcus_pseudoavium, Enterococcus_raffinosus, Vagococcus_teuberi, Lactobacillus_acidipiscis, Lactobacillus_acidophilus, Lactobacillus_agilis, Lactobacillus_algidus, Lactobacillus_amylovorus, Lactobacillus_animalis, Lactobacillus_antri, Lactobacillus_apis, Lactobacillus_brevis, Lactobacillus_buchneri, Lactobacillus_crispatus, Lactobacillus_crustorum, Lactobacillus_curvatus, Lactobacillus_delbrueckii, Lactobacillus_equicursoris, Lactobacillus_farciminis, Lactobacillus_fermentum, Lactobacillus_fuchuensis, Lactobacillus_gasseri, Lactobacillus_gastricus, Lactobacillus_helveticus, Lactobacillus_iners, Lactobacillus_intestinalis, Lactobacillus_johnsonii, Lactobacillus_kalixensis, Lactobacillus_kefiranofaciens, Lactobacillus_kefiri, Lactobacillus_kimbladii, Lactobacillus_kullabergensis, Lactobacillus_mindensis, Lactobacillus_mucosae, Lactobacillus_murinus, Lactobacillus_oris, Lactobacillus_otakiensis, Lactobacillus_panis, Lactobacillus_parabuchneri, Lactobacillus_paragasseri, Lactobacillus_paraplantarum, Lactobacillus_pentosus, Lactobacillus_plantarum, Lactobacillus_pontis, Lactobacillus_reuteri, Lactobacillus_rhamnosus, Lactobacillus_rogosae, Lactobacillus_ruminis, Lactobacillus_sakei, Lactobacillus_salivarius, Lactobacillus_sanfranciscensis, Lactobacillus_senmaizukei, Lactobacillus_ultunensis, Lactobacillus_vaginalis, Lactobacillus_versmoldensis, Pediococcus_acidilactici, Pediococcus_parvulus, Pediococcus_pentosaceus, Pediococcus_sp, Sharpea_azabuensis, Leuconostoc_carnosum, Leuconostoc_citreum, Leuconostoc_garlicum, Leuconostoc_gelidum, Leuconostoc_lactis, Leuconostoc_mesenteroides, Leuconostoc_pseudomesenteroides, Leucon |
| ACTINOMYCETALES | Actinobaculum_massiliense, Actinobaculum_sp_oral_taxon_183, Actinomyces_cardiffensis, Actinomyces_europaeus, Actinomyces_georgiae, Actinomyces_graevenitzii, Actinomyces_hongkongensis, Actinomyces_johnsonii, Actinomyces_massiliensis, Actinomyces_meyeri, Actinomyces_naeslundii, Actinomyces_odontolyticus, Actinomyces_oris, Actinomyces_radicidentis, Actinomyces_sp_HMSC035G02, Actinomyces_sp_HPA0247, Actinomyces_sp_ICM47, Actinomyces_sp_oral_taxon_170, Actinomyces_sp_oral_taxon_180, Actinomyces_sp_oral_taxon_181, Actinomyces_sp_oral_taxon_414, Actinomyces_sp_oral_taxon_448, Actinomyces_sp_oral_taxon_897, Actinomyces_sp_S6_Spd3, Actinomyces_turicensis, Actinomyces_viscosus, Actinotignum_timonense, Varibaculum_cambriense |
| DESULFOVIBRIONALES | Desulfomicrobium_orale, Bilophila_wadsworthia, Desulfovibrio_fairfieldensis, Desulfovibrio_legallii, Desulfovibrio_piger, Desulfovibrio_sp_An276, Desulfovibrio_sp_MES5, Desulfovibrio_vulgaris, Desulfovibrionaceae_bacterium |

**Supplementary Table 14.** Species name identified for the top 10 genus taxon groups for CRC.

| **Name of Taxa for CRC (Genus)** | **Species Name** |
| --- | --- |
| PARVIMONAS | Parvimonas_micra, Parvimonas_sp_KA00067, Parvimonas_sp_oral_taxon_110, Parvimonas_sp_oral_taxon_393 |
| PEPTOSTREPTOCOCCUS | Peptostreptococcus_anaerobius, Peptostreptococcus_sp_MV1, Peptostreptococcus_stomatis |
| FUSOBACTERIUM | Fusobacterium_equinum, Fusobacterium_gonidiaformans, Fusobacterium_hwasookii, Fusobacterium_mortiferum, Fusobacterium_naviforme, Fusobacterium_necrophorum, Fusobacterium_nucleatum, Fusobacterium_periodonticum, Fusobacterium_sp_CAG_439, Fusobacterium_sp_oral_taxon_370, Fusobacterium_ulcerans, Fusobacterium_varium |
| GEMELLA | Gemella_asaccharolytica, Gemella_bergeri, Gemella_haemolysans, Gemella_morbillorum, Gemella_sanguinis |
| DIALISTER | Dialister_invisus, Dialister_micraerophilus, Dialister_pneumosintes, Dialister_sp_CAG_357, Dialister_succinatiphilus |
| LACHNOCLOSTRIDIUM | Clostridium_aldenense, Clostridium_asparagiforme, Clostridium_bolteae, Clostridium_citroniae, Clostridium_clostridioforme, Clostridium_hylemonae, Clostridium_lavalense, Clostridium_saccharolyticum, Clostridium_scindens, Clostridium_symbiosum, Lachnoclostridium_sp_An118, Lachnoclostridium_sp_An131, Lachnoclostridium_sp_An138, Lachnoclostridium_sp_An14, Lachnoclostridium_sp_An169, Lachnoclostridium_sp_An196, Lachnoclostridium_sp_An298 |
| PREVOTELLA | Paraprevotella_clara, Paraprevotella_xylaniphila |
| STREPTOCOCCUS | Streptococcus_agalactiae, Streptococcus_anginosus_group, Streptococcus_australis, Streptococcus_cristatus, Streptococcus_downei, Streptococcus_equinus, Streptococcus_gallolyticus, Streptococcus_gordonii, Streptococcus_infantarius, Streptococcus_infantis, Streptococcus_lutetiensis, Streptococcus_macedonicus, Streptococcus_massiliensis, Streptococcus_milleri, Streptococcus_mitis, Streptococcus_mutans, Streptococcus_oralis, Streptococcus_parasanguinis, Streptococcus_parauberis, Streptococcus_pasteurianus, Streptococcus_peroris, Streptococcus_pneumoniae, Streptococcus_pseudopneumoniae, Streptococcus_salivarius, Streptococcus_salivarius_CAG_79, Streptococcus_sanguinis, Streptococcus_sinensis, Streptococcus_sobrinus, Streptococcus_sp_A12, Streptococcus_sp_F0442, Streptococcus_sp_HMSC034E03, Streptococcus_sp_HMSC067H01, Streptococcus_sp_HMSC070B10, Streptococcus_sp_HMSC071D03, Streptococcus_sp_HPH0090, Streptococcus_sp_M334, Streptococcus_sp_NLAE_zl_C503, Streptococcus_sp_oral_taxon_056, Streptococcus_sp_oral_taxon_058, Streptococcus_sp_SK643, Streptococcus_thermophilus, Streptococcus_vestibularis, Streptococcus_viridans |
| PORPHYROMONAS | Porphyromonas_asaccharolytica, Porphyromonas_canoris, Porphyromonas_endodontalis, Porphyromonas_gingivalis, Porphyromonas_somerae, Porphyromonas_sp_HMSC065F10, Porphyromonas_uenonis |
| SOLOBACTERIUM | Solobacterium_moorei |

**Supplementary Table 15. List of biomarkers derived by microBiomeGSM for all dataset.**

| **T2D** | | |
| --- | --- | --- |
| **Rank** | **Name of Microbiome (Family taxonomic level)** | **Reference** |
| **1** | LACHNOSPIRACEAE | (Wu and Park, 2022) |
| **2** | BIFIDOBACTERIACEAE | (Afolayan *et al.*, 2020) |
| **3** | RUMINOCOCCACEAE | (Therdtatha *et al.*, 2021) |
| **4** | EUBACTERIACEAE | (Wu and Park, 2022) |
| **5** | CORIOBACTERIACEAE | (Wu and Park, 2022) |
| **6** | CLOSTRIDIALES FAMILY XIII INCERTAE SEDIS | **–** |
| **7** | ERYSIPELOTRICHACEAE | (Wu and Park, 2022) |
| **8** | PEPTOSTREPTOCOCCACEAE | (Wu and Park, 2022) |
| **9** | CARNOBACTERIACEAE | **–** |
| **10** | BACTEROIDACEAE | (Therdtatha *et al.*, 2021) |
| **Rank** | **Name of Microbiome (Order taxonomic level)** | **Reference** |
| **1** | CLOSTRIDIALES | (Metwaly, Reitmeier and Haller, 2022) |
| **2** | BIFIDOBACTERIALES | **–** |
| **3** | CORIOBACTERIALES | (Khudhair *et al.*, 2022) |
| **4** | BACTEROIDALES | (Zhao *et al.*, 2022) |
| **5** | LACTOBACILLALES | **–** |
| **6** | ERYSIPELOTRICHALES | (Wang *et al.*, 2022) |
| **7** | SELENOMONADALES | (Du *et al.*, 2021) |
| **8** | VERRUCOMICROBIALES | (Geisler *et al.*, 2023) |
| **9** | METHANOBACTERIALES | **–** |
| **10** | BACILLALES | **–** |
| **Rank** | **Name of Microbiome (Genus taxonomic level)** | **Reference** |
| **1** | EUBACTERIUM | (Gradisteanu Pircalabioru *et al.*, 2022) |
| **2** | BIFIDOBACTERIUM | (Lê *et al.*, 2013) |
| **3** | BLAUTIA | (Hosomi *et al.*, 2022) |
| **4** | DOREA | (Li *et al.*, 2020) |
| **5** | LACHNOSPIRACEAE_NONAME | **–** |
| **6** | RUMINOCOCCUS | (Nam *et al.*, 2022) |
| **7** | COPROCOCCUS | (Lin *et al.*, 2022) |
| **8** | PEPTOSTREPTOCOCCUS | (Doumatey *et al.*, 2020) |
| **9** | ERYSIPELOTRICHACEAE_NONAME | **–** |
| **10** | GRANULICATELLA | (Neri-Rosario *et al.*, 2023) |
| **IBDMDB** | | |
| **Rank** | **Name of Microbiome (Family taxonomic level)** | **Reference** |
| **1** | BACTEROIDACEAE | (Liu *et al.*, 2023) |
| **2** | LACHNOSPIRACEAE | (Neurath, 2020) |
| **3** | RUMINOCOCCACEAE | (Carstens *et al.*, 2019) |
| **4** | RIKENELLACEAE | (Teofani *et al.*, 2022) |
| **5** | FIRMICUTES_UNCLASSIFIED | (Stojanov, Berlec and Štrukelj, 2020) |
| **6** | TANNERELLACEAE | (Teofani *et al.*, 2022) |
| **7** | EUBACTERIACEAE | (Alatawi *et al.*, 2022) |
| **8** | CLOSTRIDIACEAE | (Muñiz Pedrogo *et al.*, 2019) |
| **9** | VEILLONELLACEAE | (Alam *et al.*, 2020) |
| **10** | ODORIBACTERACEAE | (Colquhoun, Duncan and Grant, 2020) |
| **Rank** | **Name of Microbiome (Order taxonomic level)** | **Reference** |
| **1** | BACTEROIDALES | (Zitomersky *et al.*, 2013) |
| **2** | CLOSTRIDIALES | (Tye *et al.*, 2018) |
| **3** | FIRMICUTES_UNCLASSIFIED | (Stojanov, Berlec and Štrukelj, 2020) |
| **4** | VEILLONELLALES | (Rands, Brüssow and Zdobnov, 2019) |
| **5** | BURKHOLDERIALES | (Ananthakrishnan *et al.*, 2017) |
| **6** | METHANOMASSILIICOCCALES | – |
| **7** | DESULFOVIBRIONALES | – |
| **8** | ERYSIPELOTRICHALES | – |
| **9** | BIFIDOBACTERIALES | (Shobar *et al.*, 2016) |
| **10** | EGGERTHELLALES | – |
| **Rank** | **Name of Microbiome (Genus taxonomic level)** | **Reference** |
| **1** | BACTEROIDES | (Bloom *et al.*, 2011) |
| **2** | ALISTIPES | (Zhang *et al.*, 2021) |
| **3** | EUBACTERIUM | (Zhang *et al.*, 2013) |
| **4** | ROSEBURIA | (Faden, 2022) |
| **5** | FIRMICUTES_UNCLASSIFIED | (Stojanov, Berlec and Štrukelj, 2020) |
| **6** | PARABACTEROIDES | (Kverka *et al.*, 2011) |
| **7** | RUMINOCOCCUS | (Schirmer *et al.*, 2019) |
| **8** | COPROCOCCUS | (Shaw *et al.*, 2016) |
| **9** | BLAUTIA | (Nishino *et al.*, 2018) |
| **10** | CLOSTRIDIUM | (Issa, Ananthakrishnan and Binion, 2008) |
| **CRC** | | |
| **Rank** | **Name of Microbiome (Family taxonomic level)** | **Reference** |
| **1** | PEPTOSTREPTOCOCCACEAE | -- |
| **2** | PEPTONIPHILACEAE | -- |
| **3** | FUSOBACTERIACEAE | -- |
| **4** | BACILLALES_UNCLASSIFIED | (Sui *et al.*, 2020) |
| **5** | VEILLONELLACEAE | (Han *et al.*, 2019) |
| **6** | LACHNOSPIRACEAE | (Alhhazmi *et al.*, 2023) |
| **7** | ERYSIPELOTRICHACEAE | (Li *et al.*, 2023) |
| **8** | RUMINOCOCCACEAE | (Alhhazmi *et al.*, 2023) |
| **9** | PREVOTELLACEAE | (Yu *et al.*, 2023) |
| **10** | STREPTOCOCCACEAE | (Yang *et al.*, 2019) |
| **Rank** | **Name of Microbiome (Order taxonomic level)** | **Reference** |
| **1** | CLOSTRIDIALES | (Phipps *et al.*, 2021) |
| **2** | TISSIERELLALES | -- |
| **3** | BACTEROIDALES | (Hatcher *et al.*, 2023) |
| **4** | FUSOBACTERIALES | -- |
| **5** | BACILLALES | (Sui *et al.*, 2020) |
| **6** | VEILLONELLALES | -- |
| **7** | ERYSIPELOTRICHALES | -- |
| **8** | LACTOBACILLALES | (Sun *et al.*, 2022) |
| **9** | ACTINOMYCETALES | -- |
| **10** | DESULFOVIBRIONALES | -- |
| **Rank** | **Name of Microbiome (Genus taxonomic level)** | **Reference** |
| **1** | PARVIMONAS | (Alhhazmi *et al.*, 2023) |
| **2** | PEPTOSTREPTOCOCCUS | (H. Zhang *et al.*, 2022) |
| **3** | FUSOBACTERIUM | (Dougherty and Jobin, 2023) |
| **4** | GEMELLA | (J. Zhang *et al.*, 2022) |
| **5** | DIALISTER | (Rezasoltani *et al.*, 2023) |
| **6** | LACHNOCLOSTRIDIUM | (Alhhazmi *et al.*, 2023) |
| **7** | PREVOTELLA | (Elkholy *et al.*, 2023) |
| **8** | STREPTOCOCCUS | (Gutierrez-Angulo *et al.*, 2023) |
| **9** | PORPHYROMONAS | (Alhhazmi *et al.*, 2023) |
| **10** | SOLOBACTERIUM | (Avuthu and Guda, 2022) |
| **IBD** | | |
| **Rank** | **Name of Microbiome (Family taxonomic level)** | **Reference** |
| **1** | LACHNOSPIRACEAE | (Bourgonje *et al.*, 2022) |
| **2** | BIFIDOBACTERIACEAE | (Volkova and Ruggles, 2021) |
| **3** | CORIOBACTERIACEAE | -- |
| **4** | RUMINOCOCCACEAE | (Park *et al.*, 2022) |
| **5** | ERYSIPELOTRICHACEAE | (Cheng *et al.*, 2021) |
| **6** | CLOSTRIDIALES_FAMILY_XIII_INCERTAE_SEDIS | -- |
| **7** | EUBACTERIACEAE | (Alatawi *et al.*, 2022) |
| **8** | PEPTOSTREPTOCOCCACEAE | -- |
| **9** | CARNOBACTERIACEAE | -- |
| **10** | CLOSTRIDIACEAE | -- |
| **Rank** | **Name of Microbiome (Order taxonomic level)** | **Reference** |
| **1** | CLOSTRIDIALES | (Zhou *et al.*, 2018) |
| **2** | CORIOBACTERIALES | -- |
| **3** | BIFIDOBACTERIALES | -- |
| **4** | ERYSIPELOTRICHALES | -- |
| **5** | BACTEROIDALES | (Bosch *et al.*, 2023) |
| **6** | LACTOBACILLALES | -- |
| **7** | SELENOMONADALES | -- |
| **8** | VERRUCOMICROBIALES | -- |
| **9** | CANDIDATUS_SACCHARIBACTERIA_NONAME | -- |
| **10** | BACILLALES | -- |
| **Rank** | **Name of Microbiome (Genus taxonomic level)** | **Reference** |
| **1** | BLAUTIA | (Bourgonje *et al.*, 2022) |
| **2** | BIFIDOBACTERIUM | (Chen *et al.*, 2023) |
| **3** | EUBACTERIUM | (Zhang, Guo and Duan, 2022) |
| **4** | DOREA | (Bourgonje *et al.*, 2022) |
| **5** | COLLINSELLA | -- |
| **6** | PEPTOSTREPTOCOCCUS | -- |
| **7** | COPROCOCCUS | (Shaw *et al.*, 2016) |
| **8** | ERYSIPELOTRICHACEAE_NONAME | -- |
| **9** | LACHNOSPIRACEAE_NONAME | (Ravichandar *et al.*, 2022) |
| **10** | BACTEROIDES | (Kulecka *et al.*, 2023) |

**References**

Afolayan, A.O. *et al.* (2020) ‘Insights into the gut microbiota of Nigerian elderly with type 2 diabetes and non-diabetic elderly persons’, *Heliyon*, 6(5), p. e03971. Available at: https://doi.org/10.1016/j.heliyon.2020.e03971.

Alam, M.T. *et al.* (2020) ‘Microbial imbalance in inflammatory bowel disease patients at different taxonomic levels’, *Gut Pathogens*, 12(1), p. 1. Available at: https://doi.org/10.1186/s13099-019-0341-6.

Alatawi, H. *et al.* (2022) ‘Attributes of intestinal microbiota composition and their correlation with clinical primary non-response to anti-TNF-α agents in inflammatory bowel disease patients’, *Biomolecules and Biomedicine*, 22(3), pp. 412–426. Available at: https://doi.org/10.17305/bjbms.2021.6436.

Alhhazmi, A.A. *et al.* (2023) ‘Gut Microbial and Associated Metabolite Markers for Colorectal Cancer Diagnosis’, *Microorganisms*, 11(8), p. 2037. Available at: https://doi.org/10.3390/microorganisms11082037.

Ananthakrishnan, A.N. *et al.* (2017) ‘Gut Microbiome Function Predicts Response to Anti-integrin Biologic Therapy in Inflammatory Bowel Diseases’, *Cell Host & Microbe*, 21(5), pp. 603-610.e3. Available at: https://doi.org/10.1016/j.chom.2017.04.010.

Avuthu, N. and Guda, C. (2022) ‘Meta-Analysis of Altered Gut Microbiota Reveals Microbial and Metabolic Biomarkers for Colorectal Cancer’, *Microbiology Spectrum*, 10(4), pp. e00013-22. Available at: https://doi.org/10.1128/spectrum.00013-22.

Bloom, S.M. *et al.* (2011) ‘Commensal Bacteroides Species Induce Colitis in Host-Genotype-Specific Fashion in a Mouse Model of Inflammatory Bowel Disease’, *Cell Host & Microbe*, 9(5), pp. 390–403. Available at: https://doi.org/10.1016/j.chom.2011.04.009.

Bosch, D.E. *et al.* (2023) ‘Structural disruption of Ntox15 nuclease effector domains by immunity proteins protects against type VI secretion system intoxication in Bacteroidales’, *mBio*, 14(4), pp. e01039-23. Available at: https://doi.org/10.1128/mbio.01039-23.

Bourgonje, A.R. *et al.* (2022) ‘Patients With Inflammatory Bowel Disease Show IgG Immune Responses Towards Specific Intestinal Bacterial Genera’, *Frontiers in Immunology*, 13. Available at: https://www.frontiersin.org/articles/10.3389/fimmu.2022.842911 (Accessed: 19 September 2023).

Carstens, A. *et al.* (2019) ‘The Gut Microbiota in Collagenous Colitis Shares Characteristics With Inflammatory Bowel Disease-Associated Dysbiosis’, *Clinical and Translational Gastroenterology*, 10(7), p. e00065. Available at: https://doi.org/10.14309/ctg.0000000000000065.

Chen, Y. *et al.* (2023) ‘Exploiting lactic acid bacteria for inflammatory bowel disease: A recent update’, *Trends in Food Science & Technology*, 138, pp. 126–140. Available at: https://doi.org/10.1016/j.tifs.2023.06.007.

Cheng, S. *et al.* (2021) ‘Altered gut microbiome in FUT2 loss-of-function mutants in support of personalized medicine for inflammatory bowel diseases’, *Journal of Genetics and Genomics*, 48(9), pp. 771–780. Available at: https://doi.org/10.1016/j.jgg.2021.08.003.

Colquhoun, C., Duncan, M. and Grant, G. (2020) ‘Inflammatory Bowel Diseases: Host-Microbial-Environmental Interactions in Dysbiosis’, *Diseases*, 8(2), p. 13. Available at: https://doi.org/10.3390/diseases8020013.

Dougherty, M.W. and Jobin, C. (2023) ‘Intestinal bacteria and colorectal cancer: etiology and treatment’, *Gut Microbes*, 15(1), p. 2185028. Available at: https://doi.org/10.1080/19490976.2023.2185028.

Doumatey, A.P. *et al.* (2020) ‘Gut Microbiome Profiles Are Associated With Type 2 Diabetes in Urban Africans’, *Frontiers in Cellular and Infection Microbiology*, 10. Available at: https://www.frontiersin.org/articles/10.3389/fcimb.2020.00063 (Accessed: 4 July 2023).

Du, X. *et al.* (2021) ‘Alteration of gut microbial profile in patients with diabetic nephropathy’, *Endocrine*, 73(1), pp. 71–84. Available at: https://doi.org/10.1007/s12020-021-02721-1.

Elkholy, A. *et al.* (2023) ‘Microbiome diversity in African American, European American, and Egyptian colorectal cancer patients’, *Heliyon*, 9(7), p. e18035. Available at: https://doi.org/10.1016/j.heliyon.2023.e18035.

Faden, H. (2022) ‘The Role of Faecalibacterium, Roseburia, and Butyrate in Inflammatory Bowel Disease’, *Digestive Diseases*, 40(6), pp. 793–795. Available at: https://doi.org/10.1159/000522247.

Geisler, C. *et al.* (2023) ‘Gut microbiome alterations are differentially associated with hand grip strength and type 2 diabetes mellitus.’, in *Diabetologie und Stoffwechsel*. *Diabetes Kongress 2023 - 57. Jahrestagung der DDG*, Georg Thieme Verlag, p. P 143. Available at: https://doi.org/10.1055/s-0043-1768005.

Gradisteanu Pircalabioru, G. *et al.* (2022) ‘Snapshot into the Type-2-Diabetes-Associated Microbiome of a Romanian Cohort’, *International Journal of Molecular Sciences*, 23(23), p. 15023. Available at: https://doi.org/10.3390/ijms232315023.

Gutierrez-Angulo, M. *et al.* (2023) ‘Microbiota composition and its impact on DNA methylation in colorectal cancer’, *Frontiers in Genetics*, 14. Available at: https://www.frontiersin.org/articles/10.3389/fgene.2023.1037406 (Accessed: 20 September 2023).

Han, S. *et al.* (2019) ‘Intestinal microorganisms involved in colorectal cancer complicated with dyslipidosis’, *Cancer Biology & Therapy*, 20(1), pp. 81–89. Available at: https://doi.org/10.1080/15384047.2018.1507255.

Hatcher, C. *et al.* (2023) ‘Application of Mendelian randomization to explore the causal role of the human gut microbiome in colorectal cancer’, *Scientific Reports*, 13(1), p. 5968. Available at: https://doi.org/10.1038/s41598-023-31840-0.

Hosomi, K. *et al.* (2022) ‘Oral administration of Blautia wexlerae ameliorates obesity and type 2 diabetes via metabolic remodeling of the gut microbiota’, *Nature Communications*, 13(1), p. 4477. Available at: https://doi.org/10.1038/s41467-022-32015-7.

Issa, M., Ananthakrishnan, A.N. and Binion, D.G. (2008) ‘Clostridium difficile and inflammatory bowel disease’, *Inflammatory Bowel Diseases*, 14(10), pp. 1432–1442. Available at: https://doi.org/10.1002/ibd.20500.

Khudhair, Z. *et al.* (2022) ‘Administration of Hookworm Excretory/Secretory Proteins Improves Glucose Tolerance in a Mouse Model of Type 2 Diabetes’, *Biomolecules*, 12(5), p. 637. Available at: https://doi.org/10.3390/biom12050637.

Kulecka, M. *et al.* (2023) ‘Diarrheal-associated gut dysbiosis in cancer and inflammatory bowel disease patients is exacerbated by Clostridioides difficile infection’, *Frontiers in Cellular and Infection Microbiology*, 13. Available at: https://www.frontiersin.org/articles/10.3389/fcimb.2023.1190910 (Accessed: 20 September 2023).

Kverka, M. *et al.* (2011) ‘Oral administration of Parabacteroides distasonis antigens attenuates experimental murine colitis through modulation of immunity and microbiota composition’, *Clinical and Experimental Immunology*, 163(2), pp. 250–259. Available at: https://doi.org/10.1111/j.1365-2249.2010.04286.x.

Lê, K.-A. *et al.* (2013) ‘Alterations in fecal Lactobacillus and Bifidobacterium species in type 2 diabetic patients in Southern China population’, *Frontiers in Physiology*, 3. Available at: https://www.frontiersin.org/articles/10.3389/fphys.2012.00496 (Accessed: 4 July 2023).

Li, H. *et al.* (2023) ‘Identifying and ranking causal microbial biomarkers for colorectal cancer at different cancer subsites and stages: a Mendelian randomization study’, *Frontiers in Oncology*, 13. Available at: https://www.frontiersin.org/articles/10.3389/fonc.2023.1224705 (Accessed: 20 September 2023).

Li, Q. *et al.* (2020) ‘Implication of the gut microbiome composition of type 2 diabetic patients from northern China’, *Scientific Reports*, 10(1), p. 5450. Available at: https://doi.org/10.1038/s41598-020-62224-3.

Lin, R. *et al.* (2022) ‘Gut Microbiota Mediate Melatonin Signaling in Association With Type 2 Diabetes’, *Current Developments in Nutrition*, 6, p. 1019. Available at: https://doi.org/10.1093/cdn/nzac069.024.

Liu, M. *et al.* (2023) ‘Oxymatrine ameliorated experimental colitis via mechanisms involving inflammatory DCs, gut microbiota and TLR/NF-κB pathway’, *International Immunopharmacology*, 115, p. 109612. Available at: https://doi.org/10.1016/j.intimp.2022.109612.

Metwaly, A., Reitmeier, S. and Haller, D. (2022) ‘Microbiome risk profiles as biomarkers for inflammatory and metabolic disorders’, *Nature Reviews Gastroenterology & Hepatology*, 19(6), pp. 383–397. Available at: https://doi.org/10.1038/s41575-022-00581-2.

Muñiz Pedrogo, D.A. *et al.* (2019) ‘An Increased Abundance of Clostridiaceae Characterizes Arthritis in Inflammatory Bowel Disease and Rheumatoid Arthritis: A Cross-sectional Study’, *Inflammatory Bowel Diseases*, 25(5), pp. 902–913. Available at: https://doi.org/10.1093/ibd/izy318.

Nam, Y. *et al.* (2022) ‘Heat-Killed Lactiplantibacillus plantarum LRCC5314 Mitigates the Effects of Stress-Related Type 2 Diabetes in Mice via Gut Microbiome Modulation’, 32(3), pp. 324–332. Available at: https://doi.org/10.4014/jmb.2111.11008.

Neri-Rosario, D. *et al.* (2023) ‘Dysbiosis signatures of gut microbiota and the progression of type 2 diabetes: a machine learning approach in a Mexican cohort’, *Frontiers in Endocrinology*, 14. Available at: https://doi.org/10.3389/fendo.2023.1170459.

Neurath, M.F. (2020) ‘Host–microbiota interactions in inflammatory bowel disease’, *Nature Reviews Gastroenterology & Hepatology*, 17(2), pp. 76–77. Available at: https://doi.org/10.1038/s41575-019-0248-1.

Nishino, K. *et al.* (2018) ‘Analysis of endoscopic brush samples identified mucosa-associated dysbiosis in inflammatory bowel disease’, *Journal of Gastroenterology*, 53(1), pp. 95–106. Available at: https://doi.org/10.1007/s00535-017-1384-4.

Park, Y.E. *et al.* (2022) ‘Microbial changes in stool, saliva, serum, and urine before and after anti-TNF-α therapy in patients with inflammatory bowel diseases’, *Scientific Reports*, 12(1), p. 6359. Available at: https://doi.org/10.1038/s41598-022-10450-2.

Phipps, O. *et al.* (2021) ‘Oral and Intravenous Iron Therapy Differentially Alter the On- and Off-Tumor Microbiota in Anemic Colorectal Cancer Patients’, *Cancers*, 13(6), p. 1341. Available at: https://doi.org/10.3390/cancers13061341.

Rands, C.M., Brüssow, H. and Zdobnov, E.M. (2019) ‘Comparative genomics groups phages of Negativicutes and classical Firmicutes despite different Gram-staining properties’, *Environmental Microbiology*, 21(11), pp. 3989–4001. Available at: https://doi.org/10.1111/1462-2920.14746.

Ravichandar, J.D. *et al.* (2022) ‘Strain level and comprehensive microbiome analysis in inflammatory bowel disease via multi-technology meta-analysis identifies key bacterial influencers of disease’, *Frontiers in Microbiology*, 13. Available at: https://www.frontiersin.org/articles/10.3389/fmicb.2022.961020 (Accessed: 20 September 2023).

Rezasoltani, S. *et al.* (2023) ‘Oral Microbiota as Novel Biomarkers for Colorectal Cancer Screening’, *Cancers*, 15(1), p. 192. Available at: https://doi.org/10.3390/cancers15010192.

Schirmer, M. *et al.* (2019) ‘Microbial genes and pathways in inflammatory bowel disease’, *Nature Reviews Microbiology*, 17(8), pp. 497–511. Available at: https://doi.org/10.1038/s41579-019-0213-6.

Shaw, K.A. *et al.* (2016) ‘Dysbiosis, inflammation, and response to treatment: a longitudinal study of pediatric subjects with newly diagnosed inflammatory bowel disease’, *Genome Medicine*, 8(1), p. 75. Available at: https://doi.org/10.1186/s13073-016-0331-y.

Shobar, R.M. *et al.* (2016) ‘The Effects of Bowel Preparation on Microbiota-Related Metrics Differ in Health and in Inflammatory Bowel Disease and for the Mucosal and Luminal Microbiota Compartments’, *Clinical and Translational Gastroenterology*, 7(2), p. e143. Available at: https://doi.org/10.1038/ctg.2015.54.

Stojanov, S., Berlec, A. and Štrukelj, B. (2020) ‘The Influence of Probiotics on the Firmicutes/Bacteroidetes Ratio in the Treatment of Obesity and Inflammatory Bowel disease’, *Microorganisms*, 8(11), p. 1715. Available at: https://doi.org/10.3390/microorganisms8111715.

Sui, X. *et al.* (2020) ‘The relationship between KRAS gene mutation and intestinal flora in tumor tissues of colorectal cancer patients’, *Annals of Translational Medicine*, 8(17), pp. 1085–1085. Available at: https://doi.org/10.21037/atm-20-5622.

Sun, L. *et al.* (2022) ‘The difference of human gut microbiome in colorectal cancer with and without metastases’, *Frontiers in Oncology*, 12. Available at: https://www.frontiersin.org/articles/10.3389/fonc.2022.982744 (Accessed: 20 September 2023).

Teofani, A. *et al.* (2022) ‘Intestinal Taxa Abundance and Diversity in Inflammatory Bowel Disease Patients: An Analysis including Covariates and Confounders’, *Nutrients*, 14(2), p. 260. Available at: https://doi.org/10.3390/nu14020260.

Therdtatha, P. *et al.* (2021) ‘Gut Microbiome of Indonesian Adults Associated with Obesity and Type 2 Diabetes: A Cross-Sectional Study in an Asian City, Yogyakarta’, *Microorganisms*, 9(5), p. 897. Available at: https://doi.org/10.3390/microorganisms9050897.

Tye, H. *et al.* (2018) ‘NLRP1 restricts butyrate producing commensals to exacerbate inflammatory bowel disease’, *Nature Communications*, 9(1), p. 3728. Available at: https://doi.org/10.1038/s41467-018-06125-0.

Volkova, A. and Ruggles, K.V. (2021) ‘Predictive Metagenomic Analysis of Autoimmune Disease Identifies Robust Autoimmunity and Disease Specific Microbial Signatures’, *Frontiers in Microbiology*, 12. Available at: https://www.frontiersin.org/articles/10.3389/fmicb.2021.621310 (Accessed: 20 September 2023).

Wang, C. *et al.* (2022) ‘Uygur type 2 diabetes patient fecal microbiota transplantation disrupts blood glucose and bile acid levels by changing the ability of the intestinal flora to metabolize bile acids in C57BL/6 mice’, *BMC Endocrine Disorders*, 22(1), p. 236. Available at: https://doi.org/10.1186/s12902-022-01155-8.

Wu, X. and Park, S. (2022) ‘Fecal Bacterial Community and Metagenome Function in Asians with Type 2 Diabetes, According to Enterotypes’, *Biomedicines*, 10(11), p. 2998. Available at: https://doi.org/10.3390/biomedicines10112998.

Yang, Y. *et al.* (2019) ‘Prospective study of oral microbiome and colorectal cancer risk in low-income and African American populations’, *International Journal of Cancer*, 144(10), pp. 2381–2389. Available at: https://doi.org/10.1002/ijc.31941.

Yu, H. *et al.* (2023) ‘Fecal microbiota transplantation inhibits colorectal cancer progression: Reversing intestinal microbial dysbiosis to enhance anti-cancer immune responses’, *Frontiers in Microbiology*, 14. Available at: https://www.frontiersin.org/articles/10.3389/fmicb.2023.1126808 (Accessed: 20 September 2023).

Zhang, H. *et al.* (2022) ‘Microbiome analysis reveals universal diagnostic biomarkers for colorectal cancer across populations and technologies’, *Frontiers in Microbiology*, 13. Available at: https://www.frontiersin.org/articles/10.3389/fmicb.2022.1005201 (Accessed: 20 September 2023).

Zhang, J. *et al.* (2022) ‘Expansion of Colorectal Cancer Biomarkers Based on Gut Bacteria and Viruses’, *Cancers*, 14(19), p. 4662. Available at: https://doi.org/10.3390/cancers14194662.

Zhang, J., Guo, Y. and Duan, L. (2022) ‘Features of Gut Microbiome Associated With Responses to Fecal Microbiota Transplantation for Inflammatory Bowel Disease: A Systematic Review’, *Frontiers in Medicine*, 9. Available at: https://www.frontiersin.org/articles/10.3389/fmed.2022.773105 (Accessed: 19 September 2023).

Zhang, T. *et al.* (2013) ‘[Changes of fecal flora and its correlation with inflammatory indicators in patients with inflammatory bowel disease]’, *Nan fang yi ke da xue xue bao = Journal of Southern Medical University*, 33(10), pp. 1474–1477.

Zhang, X. *et al.* (2021) ‘Prevention and Alleviation of Dextran Sulfate Sodium Salt-Induced Inflammatory Bowel Disease in Mice With Bacillus subtilis-Fermented Milk via Inhibition of the Inflammatory Responses and Regulation of the Intestinal Flora’, *Frontiers in Microbiology*, 11. Available at: https://www.frontiersin.org/articles/10.3389/fmicb.2020.622354 (Accessed: 6 January 2023).

Zhao, Z. *et al.* (2022) ‘Myricetin relieves the symptoms of type 2 diabetes mice and regulates intestinal microflora’, *Biomedicine & Pharmacotherapy*, 153, p. 113530. Available at: https://doi.org/10.1016/j.biopha.2022.113530.

Zhou, Y. *et al.* (2018) ‘Gut Microbiota Offers Universal Biomarkers across Ethnicity in Inflammatory Bowel Disease Diagnosis and Infliximab Response Prediction’, *mSystems*, 3(1), p. 10.1128/msystems.00188-17. Available at: https://doi.org/10.1128/msystems.00188-17.

Zitomersky, N.L. *et al.* (2013) ‘Characterization of Adherent Bacteroidales from Intestinal Biopsies of Children and Young Adults with Inflammatory Bowel Disease’, *PLOS ONE*, 8(6), p. e63686. Available at: https://doi.org/10.1371/journal.pone.0063686.
